# Molecular mechanisms of native ligand selectivity in catecholamine G protein-coupled receptors

**DOI:** 10.1038/s41467-026-71361-8

**Published:** 2026-04-23

**Authors:** Nour Aldin Kahlous, Maiju K. Rinne, Xin Zhang, Yanying Li, Yue Chen, Aikaterini Motso, Kaixuan Gao, Christina Bergqvist, Hongda Sheng, Yi Wang, Israel Cabeza de Vaca, Alejandro Díaz-Holguín, Philip Ullmann, Tore Bengtsson, Volker M. Lauschke, Jyrki P. Kukkonen, Lucie Delemotte, Shane C. Wright, Xiangyu Liu, Dan Larhammar, Jens Carlsson

**Affiliations:** 1 Science for Life Laboratory, Department of Cell and Molecular Biology, Uppsala University, Uppsala, Sweden; 2 Department of Medical Cell Biology, Uppsala University, Uppsala, Sweden; 3 Department of Pharmacology, Medicum, University of Helsinki, Helsinki, Finland; 4 State Key Laboratory of Membrane Biology, Tsinghua-Peking Center for Life Sciences, Tsinghua University, Beijing, China; 5 Beijing Frontier Research Center for Biological Structure, School of Pharmaceutical Sciences, Tsinghua University, Beijing, China; 6 Science for Life Laboratory, Department of Applied Physics, KTH Royal Institute of Technology, Stockholm, Sweden; 7 Department of Physiology and Pharmacology, Karolinska Institutet, Stockholm, Sweden; 8 Department of Molecular Biosciences, The Wenner-Gren Institute, Stockholm University, Stockholm, Sweden; 9 College of Pharmaceutical Sciences, Zhejiang University, Hangzhou, China; 10 Innovation Institute for Artificial Intelligence in Medicine of Zhejiang University, Hangzhou, China; 11 National Key Laboratory of Chinese Medicine Modernization, Innovation Center of Yangtze River Delta, Zhejiang University, Jiaxing, China; 12 Center for Molecular Medicine, Karolinska Institutet and University Hospital, Stockholm, Sweden; 13 Dr Margarete Fischer-Bosch Institute of Clinical Pharmacology, Stuttgart, Germany; 14 University of Tübingen, Tübingen, Germany; 15 Department of Pharmacy, the Second Xiangya Hospital, Central South University, Changsha, China

**Keywords:** Computational biology and bioinformatics, Computational biophysics, Cryoelectron microscopy, Pharmacology, G protein-coupled receptors

## Abstract

Activation of G protein-coupled receptors (GPCRs) by extracellular ligands is crucial for cellular communication and modulates numerous physiological processes. Despite sharing highly similar orthosteric binding sites, catecholamine GPCRs exhibit exquisite selectivity for their native agonists, even among nearly identical chemical messengers. However, the molecular basis and evolution of receptor selectivity remain poorly understood. To elucidate the structural mechanisms of GPCR selectivity, we focus on the prototypical human β_2_-adrenergic and D_1_ dopaminergic receptors, which are important drug targets and respond to the catecholamines adrenaline/noradrenaline and dopamine, respectively. Guided by structural and sequence data, we identify a small set of residues responsible for ligand selectivity. By exchanging residues at four positions in the β-adrenergic receptors and seven in the D_1_-like dopaminergic receptors, we swap the pharmacological profiles of the two subfamilies. Unexpectedly, the switch in selectivity not only involves residues interacting with the ligand, but is also controlled by regions outside the orthosteric binding site. Cryo-electron microscopy structures and computational models of the mutant receptors identify distinct molecular mechanisms contributing to selectivity in a concerted manner. Our findings provide insights into GPCR evolution and highlight strategies for protein engineering and drug design.

## Abstract (web summary)

The molecular basis and evolution of GPCR selectivity for chemical messengers are poorly understood. Here, authors use an interdisciplinary approach to identify mechanisms of ligand selectivity in adrenergic and dopamine receptors.

## Introduction

Many functions in animals rely on a complex interplay between chemical messengers and cell-surface receptors triggering biochemical processes within cells. The large repertoire of G protein-coupled receptors (GPCRs) responds to a diverse array of endogenous and exogenous compounds by activating intracellular signal transducers such as heterotrimeric G proteins^1–5^. The large expansion and diversification of this superfamily is likely to have enabled the evolution of complex multi-cellular life forms^6,7^. The >800 GPCRs in the human genome share a common architecture with seven transmembrane helices connected by intra- and extracellular loops, and their orthosteric binding sites exhibit exquisite ligand selectivity^8,9^. This signaling machinery allows precise regulation of intracellular processes in response to extracellular stimuli. Due to their important roles in many physiological processes, GPCRs are also attractive therapeutic targets, and >30% of all approved drugs modulate members of this superfamily^10^.

Adrenaline, noradrenaline, and dopamine are essential chemical messengers that are recognized by 14 human catecholamine GPCRs^11,12^. At the time of their discovery, the receptors were classified into families based on pharmacological criteria, with each subtype being selective either for dopamine or adrenaline/noradrenaline. The nine adrenergic receptors were divided into α_1_, α_2_ and β subtypes^11^, and the five dopaminergic receptors were categorized into two subfamilies (D_1_- and D_2_-like receptors)^12^. The adrenergic and dopaminergic receptors belong to a large group of closely related aminergic GPCRs, which are activated by biogenic amines (e.g., acetylcholine, histamine, and serotonin). This monoaminergic system likely evolved in stem-bilaterians more than 650 million years ago^7^. Intriguingly, classification of the aminergic receptors based on phylogenetic analysis does not lead to the same grouping as the pharmacological profiles. The group of D_1_-like dopaminergic receptors is more closely related to the β-adrenergic receptors than to the D_2_-like subtypes. Conversely, the D_2_-like receptors cluster with α_2_-adrenergic receptors in the phylogenetic tree, indicating that they share a more recent common ancestor^13–15^. Receptors with the same ligand preference are therefore not necessarily the most closely related evolutionarily. Instead, receptors that couple to the same G protein tend to cluster together in the phylogenetic tree. These relationships illustrate the complexity of receptor evolution and suggest that selectivity for different catecholamines has emerged independently on multiple occasions during the diversification of the GPCR family. A central question for understanding the evolution of novel receptor functions is how changes in amino acid sequence alter discrimination between neurotransmitters and hormones.

**Fig. 1:**
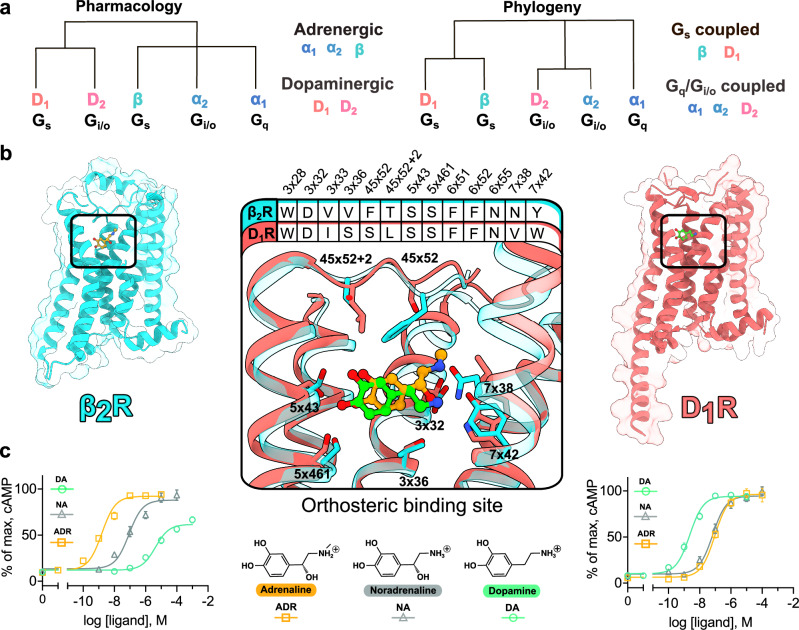
Selective receptor recognition of catecholamines. **a** Analysis of pharmacological properties and sequence-based phylogeny led to different classification of the catecholamine GPCRs^14^. **b** Comparison of the structures of β_2_R (cyan cartoon, PDB accession code: 4LDO) and D_1_R (light red cartoon, PDB accession code: 7LJD) bound to adrenaline (orange ball and sticks) and dopamine (green ball and sticks), respectively. The orthosteric binding sites of the β_2_R and D_1_R (defined as the residues within 4 Å of the native ligands) are highly similar. Side chains are shown as sticks and labeled with generic residue numbers. **c** Chemical structures of adrenaline (ADR), noradrenaline (NA), and dopamine (DA) together with concentration-response curves from cAMP assays for β_2_R (left panel) and D_1_R (right panel). The data are presented as mean ± SEM from at least three independent experiments. Source data are provided as a Source Data file.

In this work, we study the molecular mechanisms underlying native ligand selectivity of the prototypical β_2_-adrenergic and D_1_ dopaminergic receptors (β_2_R and D_1_R). These closely related GPCRs show strong selectivity for adrenaline/noradrenaline and dopamine, respectively, despite the high chemical similarity between these native agonists (Fig. 1). High-resolution structures of adrenergic and dopaminergic GPCRs are now available, providing detailed insights into ligand recognition and receptor activation^16–18^. However, the structures confirm that the catecholamine-binding sites of aminergic receptors are highly similar, and the molecular mechanisms governing selectivity remain poorly understood^19^. We investigate several key questions to understand the molecular mechanisms of receptor evolution. Can the receptor selectivity profiles be swapped by exchanging a small number of residues between the two receptors, and how many substitutions are required? Are the residues responsible for selectivity located within the orthosteric site or do residues outside the binding pocket also contribute? Is the selectivity mechanism conserved among adrenergic and dopaminergic receptor subtypes? We use evolutionary comparisons of receptor sequences and structural data to predict residue positions involved in ligand selectivity, followed by pharmacological characterization of receptor mutants. By exchanging residues within and outside the orthosteric binding site, the selectivity profiles of the receptors are swapped. These results, combined with experimentally determined structures of receptor mutants and molecular modeling, provide insights into the mechanisms and evolution of selective ligand recognition.

## Results

### Selective receptor recognition of catecholamines

The human β_2_R and D_1_R were selected as model systems to study the molecular mechanisms of receptor selectivity. These GPCRs are more closely related to each other evolutionarily than to α_1_- and α_2_-adrenergic and D_2_-like dopaminergic receptor subtypes, respectively (Fig. 1a). The receptors share 61% sequence identity in the transmembrane region and signal primarily via the Gα_s_ pathway. Cryo-electron microscopy (cryo-EM) and crystal structures of the receptors in complex with the native agonists adrenaline and dopamine (PDB accession codes: 4LDO and 7LJD, respectively) have revealed that the catecholamine binding sites are highly similar (Fig. 1b)^16,18^. Seven out of 13 residues in contact with the ligands are conserved, and the residues forming the key polar interactions with the catechol (S^5x43^, S^5x461^, and N^6x55^) and amine group (D^3x32^) are identical in all subtypes (superscripts represent generic residue numbering^20^ based on the Ballesteros–Weinstein system^21^). The close relationship between the β-adrenergic and D_1_-like dopaminergic receptors was also evident from sequence clustering obtained using Uniform Manifold Approximation and Projection (UMAP) analysis. Both the full sequence and binding site analysis yielded similar patterns, in which the β_2_R and D_1_R cluster closer to each other than to the other catecholamine receptors (Supplementary Fig. 1).

The selectivity profiles of the wild-type (WT) receptors were analyzed based on potency values (pEC_50_) obtained from functional assays measuring the elevation of the secondary messenger cyclic AMP (cAMP) and Gα_s_ recruitment upon receptor activation. The pEC_50_ values of adrenaline and noradrenaline at the β_2_R were 8.69 ± 0.10 and 7.09 ± 0.13, respectively, with selectivity of 2500- and 63-fold over dopamine (pEC_50_ = 5.29 ± 0.09) in the cAMP assay. At the D_1_R, the potency of dopamine was 8.48 ± 0.08 (pEC_50_) with a selectivity of 30- and 19-fold over adrenaline (pEC_50_ = 7.00 ± 0.10) and noradrenaline (pEC_50_ = 7.22 ± 0.09), respectively (Fig. 1c). In the G protein recruitment assays, comparable selectivity profiles were obtained for the β_2_R and D_1_R. In the case of the β_2_R, pEC_50_ values of 6.90 ± 0.37, 5.69 ± 0.05, and 4.52 ± 0.20 were obtained for adrenaline, noradrenaline, and dopamine, respectively (240- and 15-fold selectivity for adrenaline and noradrenaline over dopamine). For the D_1_R, pEC_50_ values of 6.73 ± 0.10, 5.32 ± 0.09, and 5.34 ± 0.01 were obtained for dopamine, adrenaline, and noradrenaline, respectively (26- and 25-fold selectivity for dopamine over adrenaline and noradrenaline) (Supplementary Fig. 2).

### Identification of receptor selectivity hotspot regions

**Fig. 2:**
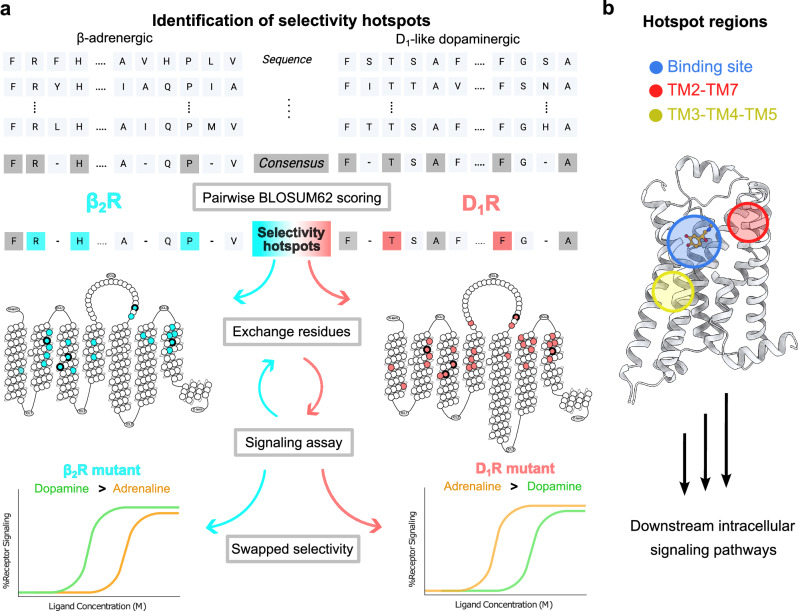
Identification of receptor selectivity hotspots. **a** Selectivity hotspots were identified from alignments of β-adrenergic and D_1_-like dopaminergic receptor sequences. Consensus sequences were pairwise scored using the BLOSUM62 matrix to identify selectivity hotspot regions. Based on analysis of structural and sequence data, mutagenesis was performed by exchanging residues between the receptors, followed by the evaluation of the mutants using cAMP assays. The mutated positions for each receptor are highlighted in cyan and light red for the β_2_R and D_1_R, respectively, in the snake plots. The five prioritized hotspot positions are marked with bold circles. **b** The three hotspot regions mapped onto the 3D structure of β_2_R (PDB accession code: 4LDO): The orthosteric site (blue), TM3-TM4-TM5 (yellow) and TM2-TM7 (red) interfaces.

In order to map receptor regions involved in determining the ligand preference (selectivity hotspots), multi-species sequence alignments of the β-adrenergic and D_1_-like dopaminergic receptors were first generated (Fig. 2a). Based on the hypothesis that the mechanism of selectivity would be conserved within each subfamily, we identified positions in the transmembrane helices (TMs) and binding site region with **≥**80% conservation that were substantially different according to the BLOSUM62 substitution matrix (substitution score <0)^22^. The resulting 11 hotspot positions were inspected in the β_2_R and D_1_R structures (PDB accession codes: 4LDO^16^ and 7LJD^18^, respectively). Based on this analysis, we then deprioritized six positions that were either surface-exposed and therefore unlikely to affect ligand binding, or that were close to the G protein binding site, where mutations could interfere with the signaling assays (Supplementary Table 1). The remaining five prioritized selectivity hotspots were either interacting directly with the ligands (residue positions 3x36, 45x52, and 7x38) or located in two other distinct interhelical regions outside the binding sites (residue positions 2x60 and 3x41) (Fig. 2b).

The sequence and structural analysis led to the identification of three selectivity hotspot regions. As anticipated, the orthosteric binding site was one of the regions identified as important for receptor selectivity. These selectivity hotspot residues (3x36, 7x38, and 45x52) were located in the amine binding pocket (Figs. 1b and 2b). In the β_2_R (PDB accession code: 4LDO^16^), the β-carbon hydroxyl group of adrenaline forms a hydrogen bond with N312^7x38^ and its amine group forms a π-cation interaction with F193^45x52^ in the second extracellular loop (EL2). In the D_1_R (PDB accession code: 7LJD^18^), dopamine binds deeper in the site and does not interact with S188^45x52^ in EL2. The amine of dopamine instead interacts with hotspot residue S107^3x36^ and W312^7x42^ (V117^3x36^ and Y316^7x42^ in the β_2_R, respectively) (Fig. 1b). The two other hotspot regions were located in the TM3-TM4-TM5 (position 3×41) and TM2-TM7 (position 2x60) interfaces. Unexpectedly, neither of these residues interacted with ligands in the experimental structures and their potential roles in receptor selectivity were unclear (Fig. 2b).

Our mapping of selectivity hotspots, combined with extensive analysis of receptor structures, guided the design of β_2_R and D_1_R mutants in which residues were exchanged between the receptors. We focused primarily on residues in the hotspot and binding site regions, with the goal to swap selectivity profiles while maintaining a physiologically relevant potency (Supplementary Table 2). A physiologically relevant potency is essential for evolutionary change of receptor functions, because otherwise the receptor gene would be likely to degenerate into a pseudogene^23^. Residue exchanges between the receptors were first introduced within the binding site, followed by combinations of mutations within and outside the binding site (Fig. 2b). A majority of the mutated positions were located in the upper half of the receptors. In total, we evaluated 120 receptor mutants, which involved 21 and 28 unique positions in the β_2_R (M1–35) and D_1_R (M1–85) (Fig. 2a, Supplementary Fig. 3, and Supplementary datafile 1).

Each mutant was first assessed by evaluating the ability of adrenaline, noradrenaline, and dopamine to stimulate cAMP accumulation (Supplementary datafile 1). The cAMP-derived potencies were then used to determine selectivity profiles. As these three compounds did not lead to any response in non-transfected cells and do not permeate the plasma membrane, response in transfected cells was considered as evidence of cell surface expression. We also conducted immunostaining analysis for key mutants, which confirmed cell surface expression (>40% of wild-type, Supplementary Fig. 4). None of the key mutants showed significant difference between ligands in the maximal cAMP response (Supplementary Data 1), indicating that the selectivity profiles are not affected by expression levels^24^. Mutants with switched selectivity profiles were further characterized in Gα_s_ recruitment assays and by evaluating panels of chemical messengers.

### Binding-site mutations reduce selectivity at the expense of efficacy and potency

**Fig. 3:**
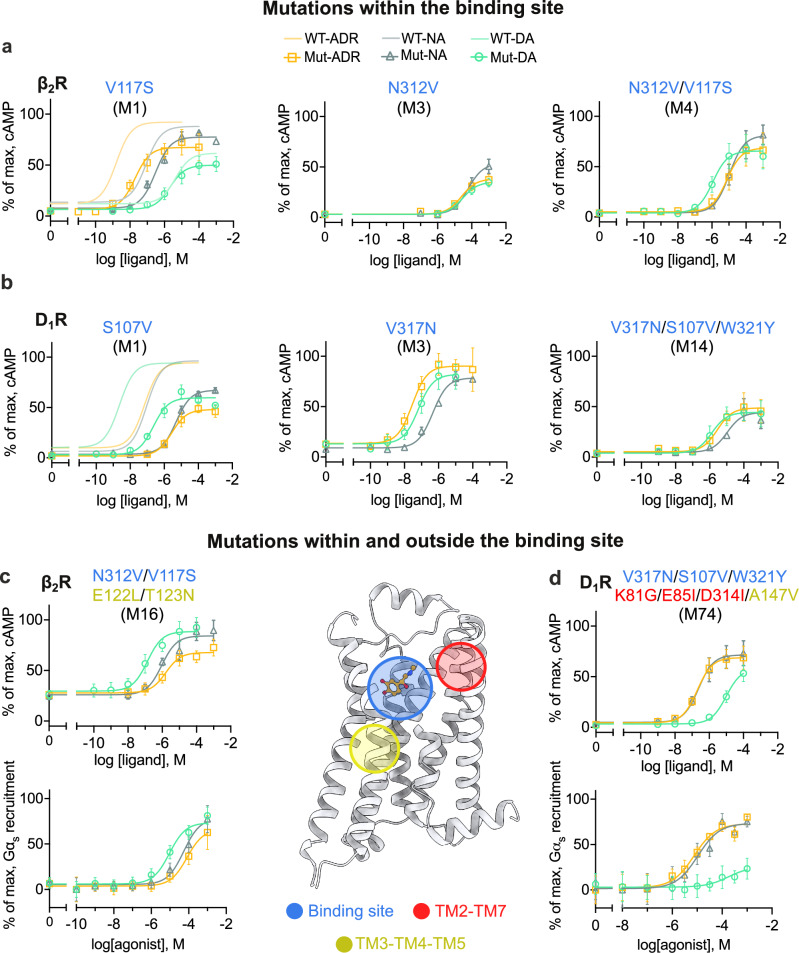
Receptor selectivity is determined by residues within and outside the orthosteric binding site. **a**, **b** cAMP concentration-response curves for the catecholamines adrenaline (ADR, orange curves), noradrenaline (NA, gray curves), and dopamine (DA, green curves) at binding site mutants of the β_2_R (M1, M3, and M4) and D_1_R (M1, M3, and M14). **c**, **d** cAMP and G protein recruitment concentration-response curves for the catecholamines at mutants of β_2_R (M16) and D_1_R (M74) in which mutations within and outside the binding site were combined. The E122L/T123N mutation led to increased basal activity, as shown by cAMP measurements. Data were normalized to the maximum response of the corresponding WT receptor, and are presented as mean ± SEM from at least three independent experiments. All receptor mutants are colored according to the hotspot regions they are located in (blue for the binding site, red for the TM2-TM7 interface, and yellow for the TM3-TM4-TM5 interface). Source data are provided as a Source Data file.

We evaluated sets of eight and twelve binding-site mutants of the β_2_R and D_1_R, respectively, in which between one and four residues were exchanged (Supplementary datafile 1). Adrenaline, noradrenaline, and dopamine showed reduced potency and efficacy compared to the wild-type at the β_2_R mutants with a single substitution in the three binding site hotspots for selectivity (V117S, F193S, and N312V at positions 3x36, 45x52, and 7x38, respectively, M1–3). The mutant receptors either remained selective for adrenaline and noradrenaline (V117S and F193S) or displayed a substantial loss of response and selectivity (N312V, Fig. 3a). The combination of the N312V and V117S mutations (M4) resulted in 10- and 9-fold selectivity for dopamine over adrenaline and noradrenaline, respectively. In this case, adrenaline (pEC_50_ = 4.88 ± 0.09) and noradrenaline (pEC_50_ = 4.91 ± 0.13) showed a 6500- and 150-fold loss of potency, respectively, whereas the potency of dopamine (pEC_50_ = 5.86 ± 0.09) was comparable to that obtained at the wild-type β_2_R (Fig. 3a). It should be noted that this mutant exhibited reduced expression compared with the wild-type β_2_R, which may contribute to the lower potencies observed in the cAMP experiments (Supplementary Fig. 4). Increasing the number of binding site mutations to three or four residues further decreased potency and efficacy, but did not increase selectivity for dopamine (Supplementary datafile 1).

The results for the D_1_R mutants were similar to those of the β_2_R (Fig. 3b and Supplementary datafile 1). The single mutant with residues exchanged in position 7x38 led to a large reduction in selectivity (V317N, M3). The potency of adrenaline at the V317N mutant (pEC_50_ = 7.40 ± 0.10) was comparable to that of the wild-type D_1_R, but the potency of dopamine was reduced by 22-fold (pEC_50_ = 7.13 ± 0.13), resulting in a two-fold selectivity for adrenaline. However, noradrenaline (pEC_50_ = 6.28 ± 0.08) remained less potent than dopamine at this mutant (Fig. 3b). The double, triple, and quadruple mutants involving 7x38 generally resulted in a large loss of potency compared to the wild-type D_1_R and several of these receptors responded equally well to the compounds, *e.g*. the triple mutant S107V/V317N/W321Y (M14, Fig. 3b).

Mutations in positions 3x36 and 7x38 resulted in a reduction of selectivity for both the β_2_R and D_1_R, indicating that these hotspots play a role in determining the pharmacological profiles. However, the altered selectivity profiles were accompanied by a loss of agonist potencies, and the selectivity profile of the D_1_R mutant was not fully switched because dopamine remained more potent than noradrenaline. To identify the molecular basis of these changes in selectivity, we performed molecular dynamics (MD) simulations of the wild-type and mutated receptors. We calculated the difference in affinity between noradrenaline and dopamine using free-energy perturbation (FEP) calculations^24^. The MD/FEP calculations reproduced the selectivity profiles of the wild-type receptors, with free energy differences of 1.9 ± 0.3 (β_2_R) and 2.3 ± 0.3 (D_1_R) kcal/mol in favor of noradrenaline and dopamine, respectively (Supplementary Fig. 5). Based on the free energy calculations, the single and double mutations were predicted to reduce selectivity, and the largest changes were obtained for the β_2_R (Supplementary Fig. 5b). These MD simulation results were in qualitative agreement with the experimental data. For example, the N312V/V117S β_2_R mutant showed a 9-fold selectivity for dopamine over noradrenaline, whereas the FEP calculations predicted only a slight dopamine selectivity (2-fold). In the case of the β_2_R, the N312V mutation (7x38 position) led to a loss of hydrogen bonding for the β-carbon hydroxyl group of noradrenaline, which was a plausible cause of the potency loss (Supplementary Fig. 5b). The V117S mutation (3x36 position) also led to a substantial loss of noradrenaline potency. This effect was likely due to electrostatic repulsion between the hydroxyl groups of the serine and β-carbon, both of which formed strong hydrogen bonds with D113^3x32^ (Supplementary Fig. 5b). In contrast, dopamine was less affected by these two mutations because this compound lacks the β-carbon hydroxyl group.

### Mutations outside the binding site can enhance potency and selectivity

To investigate the potential roles of the selectivity hotspots identified in the TM3-TM4-TM5 (position 3x41) and TM2-TM7 (position 2x60) interfaces (Fig. 3a–d), combinations of mutations within and outside the binding site were evaluated (Supplementary datafile 1).

We identified positions in the TM3-TM4-TM5 interface of the β_2_R that when mutated improved agonist potencies, but did not influence the selectivity. The addition of two mutations in this region (E122L/T123N at the conserved positions 3x41 and 3x42) to one of the β_2_R binding site mutants (V117S/N312V, M4) led to an increase in adrenaline, noradrenaline, and dopamine potencies. Compared to the wild-type β_2_R, the potency of dopamine increased by 41-fold, reaching a more physiologically relevant range (pEC_50_ = 6.90 ± 0.12) for this mutant (V117S/E122L/T123N/N312V, M16). The potencies of adrenaline and noradrenaline were 820- and 9-fold lower than at the wild-type β_2_R, resulting in a dopamine-selective receptor with 13- and 6-fold selectivity, respectively (Fig. 3c). To further assess the role of E122L/T123N, we also introduced these two mutations in the wild-type β_2_R. The E122L/T123N mutations alone (M24) substantially enhanced agonist potencies and increased basal activity, but did not alter the selectivity profile or surface expression. The mutant receptor retained the strong selectivity for adrenaline and noradrenaline that is characteristic of the wild-type β_2_R (Supplementary Fig. 6a). These results supported that introduction of the E122L/T123N mutations led to constitutive receptor activity and enhanced response to ligands.

The addition of mutations in the hotspot region located in the TM1-TM2-TM7 interface increased potency and switched selectivity of the D_1_R. In cryo-EM structures of D_1_R, the selectivity hotspot residue K81^2x60^ is part of a network of polar interactions that also includes residues E85I^2x64^ and D314^7x35^, which are conserved in the D_1_-like subfamily^18,25^. In the β_2_R, these three charged side chains are replaced by nonpolar residues. The addition of K81G/E85I/D314I to the non-selective binding site mutant (S107V/V317N/W321Y, M14; Fig. 3a) enhanced agonist potencies and led to a receptor (M79) that was selective for adrenaline and noradrenaline over dopamine (Supplementary datafile 1). Ligand potencies were then further enhanced by introducing a mutation in the TM3-TM4-TM5 hotspot interface (A147V). These results aligned with the increase in potency observed for the E122L/T123N mutation in the corresponding TM3-TM4-TM5 interface of the β_2_R. For this mutant (K81G/E85I/S107V/A147V/D314I/V317N/W321Y, M74), the potencies of adrenaline and noradrenaline were in a more physiological range (pEC_50_ = 6.67 ± 0.07 and 6.64 ± 0.10, respectively), whereas the potency of dopamine was reduced by 3400-fold (pEC_50_ = 4.95 ± 0.15) compared to the wild-type D_1_R. These potencies resulted in selectivities of 52- and 49-fold for adrenaline and noradrenaline over dopamine, respectively (Fig. 3d). To assess the effect of the mutations outside the binding site, we also introduced K81G/E85I/D314I (M82) and K81G/E85I/A147V/D314I (M85) into the wild-type D_1_R. These receptor mutants retained selectivity for the native ligand dopamine (Supplementary Fig. 6b, c, respectively). The combination of mutations within and outside the binding site was hence required to switch receptor selectivity.

Two β_2_R and D_1_R mutants with swapped selectivity profiles were further validated by measuring Gα_s_ recruitment. The β_2_R mutant with four mutations (V117S/E122L/T123N/N312V, M16) was dopamine-selective (pEC_50_ = 5.21 ± 0.13) with 6- and 11-fold higher potencies for dopamine than for noradrenaline (pEC_50_ = 4.46 ± 0.10) and adrenaline (pEC_50_ = 4.17 ± 0.18), respectively (Fig. 3c). The D_1_R mutant with seven mutations (K81G/E85I/S107V/A147V/D314I/V317N/W321Y, M74) was selective for adrenaline (pEC_50_ = 5.12 ± 0.06) and noradrenaline (pEC_50_ = 4.93 ± 0.06) with 21- and 13-fold higher potencies, respectively, than for dopamine (pEC_50_ = 3.80 ± 0.24) (Fig. 3d). The selectivity profiles of the mutant receptors obtained from the Gα_s_ recruitment assay were thus in agreement with the cAMP experiments. In the case of the β_2_R mutant, the fold selectivity was lower in the Gα_s_ recruitment assay compared with the cAMP assay, which agreed with the results obtained for the wild-type receptor.

### Receptor mutants with swapped selectivity profiles are not promiscuous

The GPCR signaling machinery is dependent on specific recognition of the native ligand over other closely related biogenic amines. To assess if the mutations affected this selectivity profile, we evaluated the activity of serotonin, histamine, and acetylcholine at the β_2_R (V117S/E122L/T123N/N312V, M16) and D_1_R (K81G/E85I/S107V/A147V/D314I/V317N/W321Y, M74) mutants. Concentrations up to 100 µM were tested and no substantial cAMP response was observed, confirming that the mutations did not lead to receptor promiscuity (Supplementary Fig. 7).

### The selectivity mechanism is conserved within the receptor subfamilies

**Fig. 4:**
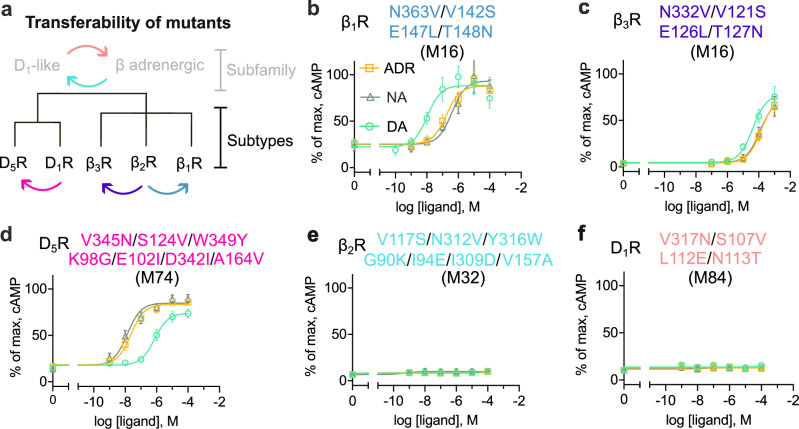
Transferability of selectivity-switching mutants. **a** Phylogenetic tree illustrating the evaluation of the transferability of the identified mutations. Mutations were transferred between subtypes within the β-adrenergic and D_1_-like dopaminergic receptor subfamilies (**b–d**) and between the receptor subfamilies (**e**, **f**). The arrows and mutants in **b–f** are color-matched. **b–d** cAMP concentration-response curves for adrenaline (ADR, orange curves), noradrenaline (NA, gray curves), and dopamine (DA, green curves) at mutants where the key mutations were transferred to other receptor subtypes (β_1_R, β_3_R, and D_5_R). The E147L/T148N mutation in β_1_R led to increased basal activity in cAMP experiments. **e**, **f** cAMP concentration–response curves for catecholamines at mutants where key mutations were transferred between receptor subfamilies, β-adrenergic (represented by β_2_R) and D_1_-like dopaminergic (represented by D_1_R), respectively. Data were normalized to the maximum response of the corresponding WT receptor, and are presented as mean ± SEM from at least three independent experiments. Source data are provided as a Source Data file.

One of the first assumptions of our approach was that the mechanism of selectivity would be conserved within each subfamily. Although the mutated residues were conserved in either the D_1_-like dopaminergic or β-adrenergic receptor subfamilies, there were also substantial differences between the sequences. Up to 45% and 23% of the residues in the TM region differ between the three human β-adrenergic and the two human D_1_-like dopaminergic receptors, respectively. To assess if the residue positions that switched selectivity were transferable within subfamilies, we introduced the four mutations of the β_2_R in the other two β-adrenergic receptors (β_1_R and β_3_R), and the seven D_1_R mutations were evaluated for the D_5_ dopaminergic receptor (D_5_R) (Fig. 4a).

The wild-type β_1_ and β_3_ subtypes are strongly selective for adrenaline and noradrenaline over dopamine (Supplementary Fig. 8). In agreement with the results obtained for the β_2_R, the four key mutations switched selectivity of the other two subtypes as well. In the case of the β_1_R mutant (V142S/E147L/T148N/N363V, M16), the pEC_50_ of dopamine was 7.99 ± 0.10, corresponding to a 100-fold increase in potency compared to the wild-type β_1_R with selectivities of 17- and 35-fold over adrenaline (pEC_50_ = 6.75 ± 0.13) and noradrenaline (pEC_50_ = 6.44 ± 0.15), respectively (Fig. 4b). The potencies of all compounds were considerably lower at the corresponding β_3_R mutant (V121S/E126L/T127N/N332V, M16, pEC_50_ = 4.39 ± 0.04, 3.83 ± 0.03, and 3.93 ± 0.03 for dopamine, adrenaline, and noradrenaline), but the receptor was slightly selective for dopamine (Fig. 4c). These results are consistent with the observation that the β_1_R subtype has the highest sequence identity to the D_1_R (41%) in the TM region, whereas β_3_R has the lowest (34%). The greater similarity in the behavior of the β_1_R and β_2_R mutants in these assays (e.g., comparable selectivity profiles and increased basal activity) is also consistent with the higher sequence identity between these receptors (66%) compared to that between β_2_R and β_3_R (55%). The D_5_R shares 77% sequence identity with D_1_R and also exhibited substantial selectivity for dopamine over adrenaline and noradrenaline in the cAMP assay (Supplementary Fig. 8). Introduction of the seven key mutations into the D_5_R (K98G/E102I/S124V/A164V/D342I/V345N/W349Y, M74) switched selectivity (Fig. 4d). Adrenaline and noradrenaline showed high potency at the mutant receptor (pEC_50_ = 7.62 ± 0.17 and 7.80 ± 0.28, respectively) with 34- and 51-fold selectivity over dopamine, respectively. These results supported that mechanisms governing receptor selectivity were conserved within the β-adrenergic and D_1_-like dopaminergic receptor subfamilies.

### The selectivity mechanism is not transferable between receptor subfamilies

Only two of the residue positions mutated to switch the selectivity of the β_2_R and D_1_R overlapped (3x36 and 7x38) and the remaining five were unique. To assess whether the selectivity mechanism in the D_1_R was transferable to the β_2_R, we introduced the corresponding mutations in the same seven positions that switched selectivity of the D_1_R into β_2_R (G90K/I94E/V117S/V157A/I309D/N312V/Y316W, M32). Conversely, four residues were mutated in the D_1_R (S107V/L112E/N113T/V317N, M84) to match the mutant that switched selectivity of the β_2_R. Both receptors were expressed on the cell surface, but no substantial cAMP response to adrenaline, noradrenaline, or dopamine was detected (Fig. 4e, f and Supplementary Fig. 4). These results supported that mechanisms governing receptor selectivity were different in the β-adrenergic and D_1_-like dopaminergic receptor subfamilies.

### Cryo-EM structures and simulations reveal the molecular basis of selectivity

The molecular mechanisms underlying selectivity were further investigated by determining cryo-EM structures of the key mutant receptors bound to G_s_ and their preferred ligand. The structure of the β_2_R mutant (V117S/E122L/T123N/N312V, M16) was solved in complex with dopamine at a resolution of 2.90 Å, whereas the structure of the D_1_R mutant (K81G/E85I/S107V/A147V/D314I/V317N/W321Y, M74) bound to adrenaline had a resolution of 2.85 Å (Supplementary Fig. 9, 10 and Supplementary Table 3). The overall receptor conformations closely resembled the activated wild-type β_2_R (Cα Root Mean Square Deviation (RMSD) = 0.54 Å to β_2_R mutant, PDB accession code: 4LDO^16^) and D_1_R (Cα RMSD = 0.61 Å to D_1_R mutant, PDB accession code: 7LJD^18^) in complex with native agonists. The conserved microswitches in class A receptor activation (*e.g*., the outward movement of TM6, and the PIF and NPxxY motifs^5^) occupied similar conformations in the mutant and wild-type receptors (Supplementary Fig. 11). To understand the swapped selectivity profiles, we examined structural differences in the regions where mutations were introduced and compared with MD simulations of wild-type and modified receptors.

**Fig. 5:**
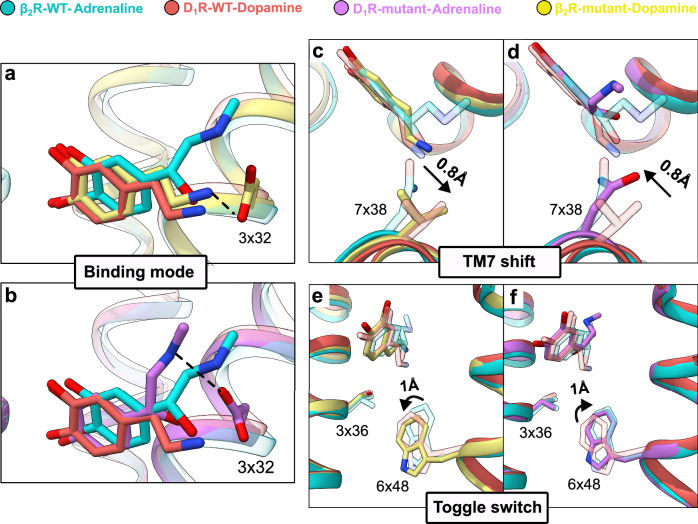
Molecular basis of swapping selectivity: mutations within the binding site. **a**, **b** Comparisons of the binding modes of adrenaline in D_1_R-M74 (violet) and dopamine in β_2_R-M16 (yellow) to those in β_2_R-WT (cyan, PDB accession code: 4LDO) and D_1_R-WT (light red, PDB accession code: 7LJD), respectively. **c**, **d** Structural comparison of changes affecting the key position 7x38 in mutant and WT receptors. **e**, **f** Structural comparison of changes in the toggle switch motif between mutant and WT receptors. Ligands are depicted in stick representations. Side chains are shown as sticks and labeled with generic numbering. Hydrogen bonds are indicated by dashed black lines. Distances and angles are labeled with black arrows. The side chains of WT receptors are shown with transparency for clarity.

The mutations introduced in the receptors influenced the agonist binding modes and the shape of the binding site (Fig. 5 and Supplementary Fig. 12). One of the key differences between the orthosteric binding sites of the wild-type β_2_R and D_1_R is that the catecholamine agonists adopt different binding modes, in which the amine group of dopamine is more deeply buried in the D_1_R pocket (Fig. 1b). The density of dopamine in the β_2_R mutant was consistent with several positions of the amine group, and one of these resembled the wild-type D_1_R-dopamine complex (Fig. 5a). Furthermore, similar binding poses of adrenaline were observed for the D_1_R mutant and wild-type β_2_R (Fig. 5b). These results suggested that the mutations at positions 7x38 and 3x36 led to a swap of agonist binding modes between the receptors, which was also consistent with MD simulations of these complexes (Supplementary Fig. 12a, b and 13a, b). In the β_2_R cryo-EM structure, the N312V^7x38^ mutation led to an approximately 0.8 Å shift of TM7 toward TM2, resulting in a binding site conformation that was more similar to that observed in the wild-type D_1_R structure (Fig. 5c). These changes may favor the more deeply buried binding mode of the amine group of dopamine (Supplementary Fig. 12a and 14a, b). Conversely, the V317N^7x38^ mutation in D_1_R led to a shift of TM7 toward TM6, which contributes to pushing adrenaline toward EL2, in agreement with the wild-type β_2_R crystal structure and MD simulations (Fig. 5b, d, Supplementary Figs. 12b, 14a, 14c). The mutations in position 3x36 (V117S^3x36^ and S107V^3x36^ in the β_2_R and D_1_R, respectively) also altered the shape and polarity of the amine-binding pocket. Notably, the 3x36 mutation slightly altered the side chain conformation of W^6x48^, a conserved microswitch in the activation of class A GPCRs (“the toggle switch”)^2,26^. In the β_2_R mutant, there was an approximately 1 Å shift downward shift of the side chain of W285^6x48^ compared to the wild-type β_2_R, leading to a side chain conformation more similar to that observed in the wild-type D_1_R structure and a deeper pocket (Fig. 5e). Conversely, the introduction of the corresponding mutant (S107V^3x36^) in D_1_R led to a shift of W285^6x48^ upward to an orientation similar to the wild-type β_2_R and a slightly shallower pocket (Fig. 5f). The structural differences observed for the ligand, 6x48 and 7x38 were relatively subtle, but were supported by MD simulations of the wild-type and mutant receptors (Supplementary Fig. 12c–f and 14d, e).

**Fig. 6:**
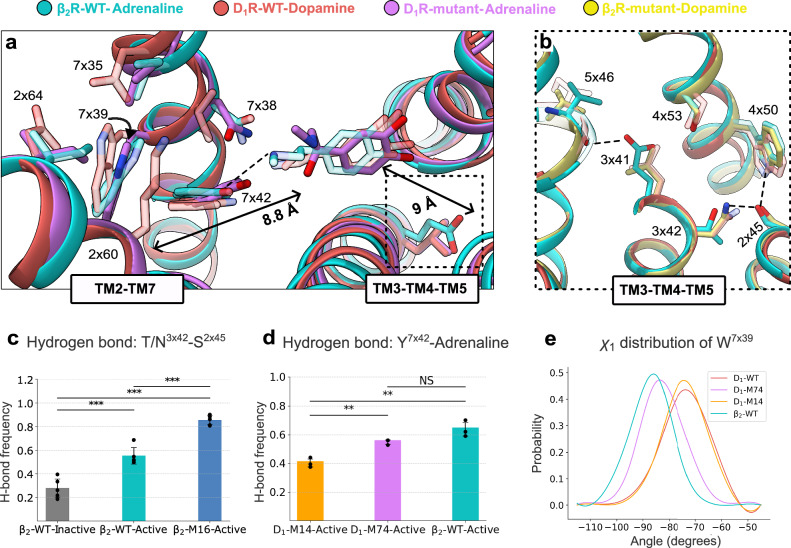
Molecular basis of swapping selectivity: mutations outside the binding site. **a** The TM3-TM4-TM5 and TM2-TM7 interfaces of D_1_R-M74-adrenaline compared to WT receptors. **b** Structural changes in TM3-TM4-TM5 interface of β_2_R-M16-dopamine compared to WT receptors. **c** The frequency of hydrogen bonding (H-bond) in MD simulations between S74^2x45^ and N/T123^3x42^ in mutant and WT receptors. Data bars represent the mean ± standard deviation from five independent simulations (gray, β_2_R-WT-inactive; cyan, β_2_R-WT-active; blue, β_2_R-M16-active). Statistical significance was assessed with a pairwise two-tailed Welch’s t-tests (β_2_R-WT-inactive vs β_2_R-WT-active, *p* = 0.00076; β_2_R-WT-inactive vs β_2_R-M16, *p* = 0.0001; β_2_R-WT-active vs β_2_R-M16, *p* = 0.00022. Significance levels: not significant (NS) *p* > 0.05, *p < 0.05, **p < 0.01, ***p < 0.001). **d** The frequency of hydrogen bonding in MD simulations between Y321/316^7x42^ and adrenaline in mutant and WT receptors. Data bars represent the mean ± SD from three independent simulations (orange, D_1_R-M14-active; violet, D_1_R-M74; cyan, β_2_R-WT-active). Statistical significance was assessed with a pairwise two-tailed Welch’s t-tests (D_1_R-M14-active vs D_1_R-M74-active, *p* = 0.00627; D_1_R-M14-active vs β_2_R-WT-active, *p* = 0.00682; D_1_R-M74-active vs β_2_R-WT-active, *p* = 0.10156. Significance levels: not significant (NS) *p* > 0.05, *p < 0.05, **p < 0.01, ***p < 0.001). **e** The distribution curves of W318/313^7x39^ *χ*_1_ dihedral angle in MD simulations of mutant and WT receptors. Data curves represent dihedral angles collected from five independent simulations (light red, D_1_R-WT; orange, D_1_R-M14-active; violet, D_1_R-M74; cyan, β_2_R-WT-active). Ligands are depicted in stick representations. Side chains are shown as sticks and labeled with generic numbering. Hydrogen bonds are indicated by dashed black lines. Distances and angles are labeled with black arrows. The side chains of WT receptors are shown with transparency for clarity. Source data are provided as a Source Data file.

Mutations in the TM3-TM4-TM5 and TM2-TM7 interfaces influenced selectivity and receptor response, but none of the mutated residues were in direct contact with the ligands (Fig. 6a). Mutations in the TM3-TM4-TM5 interface of the β_2_R, in which the selectivity hotspot residue 3x41 was located, increased agonist potency and basal activity. Analysis of the β_2_R mutant structure suggested that the E122L/T123N mutation influenced the interaction network controlling conformational changes involved in activation. One of the characteristics of β_2_R activation is that agonist binding leads to a bulge of TM5, which favors active-like conformations of the conserved PIF motif. This structural change then propagates the signal from the orthosteric binding pocket to the G protein binding site^27^. Activation leads to a rotation of TM3, which rearranges the polar interaction network involving residues E122^3x41^ and T123^3x42^. A hydrogen bond between E122^3x41^ and S161^4x53^ is weakened upon activation, and E122^3x41^ instead interacts with the backbone of V206^5x46^. The TM3 rotation also leads to a stronger hydrogen bond between T123^3x42^ and S74^2x45^ in the active conformation (Fig. 6b). In the structure of the β_2_R mutant, the network of interactions connecting the TM5 bulge to E122^3x41^ was broken as the introduced leucine side chain (E122L) is unable to form hydrogen bonds. The side-chain conformations and interactions of L122^3x41^ and N123^3x42^ in the β_2_R mutant were essentially identical to those observed in the active D_1_R structure. Moreover, the TM3 rotation upon activation appeared to be further stabilized by the T123N mutation (Fig. 6b). Our observations in the cryo-EM structures were supported by MD simulations of wild-type and mutant receptors (Supplementary Fig. 12g). In order to gain further insight into the molecular mechanism underlying the basal activity observed in the cAMP assays (Fig. 3c), we used MD simulations to calculate free energy landscapes describing activation of the wild-type and mutant β_2_R in the apo state. The free energy landscapes revealed that the M16 mutations (V117S/E122L/T123N/N312V) shift the receptor conformation towards more active-like conformations (Supplementary Fig. 15). This result is consistent with the increased basal activity observed experimentally for the M16 and M24 mutants (Fig. 3c and Supplementary Fig. 6a) and the formation of an active-like hydrogen-bonding network in the TM2-TM3 interface in simulations of these receptors (Fig. 6b, c and Supplementary Fig. 16a). MD simulations of the β_2_R wild-type and several mutants, combined with network analysis, also revealed a communication pathway linking the key binding-site residue S207^5x461^ to the mutated position 3x42 via residue I121^3x40^ (Supplementary Fig. 17a). I121^3x40^ is part of the conserved PIF motif, an important microswitch for receptor activation that has been suggested to connect the orthosteric pocket with the G protein binding site^26^.

The combination of mutations in the binding site and the TM2-TM7 interface switched the selectivity profile of the D_1_R and increased agonist potencies. Notably, neither the binding site mutations (S107V/V317N/W321Y, M14) nor the mutations outside the binding site (K81G/E85I/D314I, M82) alone switched selectivity, suggesting communication between these regions (Fig. 6a). Analysis of the wild-type D_1_R structure showed that K81^2x60^, E85^2x64^, and D314^7x35^ are part of an interaction network that indirectly influences the binding site residue W321^7x42^. Both E85^2x64^ and D314^7x35^ can form hydrogen bonds with the W318^7x39^ side chain, and K81^2x60^ and W318^7x39^ together stabilize binding site residue W321^7x42^ with van der Waals and π–π stacking interactions. In the cryo-EM structure of the D_1_R mutant, the mutated residues G81^2x60^, I85^2x64^, and I314^7x35^ led to conformations of W318^7x39^ and Y321^7x42^ that were more similar to those observed in the wild-type β_2_R crystal structure. These observations were further supported by comparing MD simulations of wild-type and mutant receptors (Supplementary Fig. 12h). Network analysis of MD simulations showed that residue positions 2x60, 2x64, and 7x35 are part of two communities, groups of residues with highly correlated motions, that connect the residues within and outside the binding site (Supplementary Fig. 17b). The network of interactions in the D_1_R mutant stabilized the W318^7x39^ and Y321^7x42^ side chains in conformations characteristic of the wild-type β_2_R in the MD simulations, thereby favoring the binding of noradrenaline and adrenaline over dopamine, in agreement with the experimentally determined selectivity profile (Fig. 6d, e). The MD simulations also indicated that there was a synergistic effect on the W318^7x39^ rotamer distribution from combining mutations within and outside the binding site (Supplementary Fig. 16b).

During the course of this study, major advances in machine learning-based protein structure prediction allowed us to model the wild-type and mutant receptors using an approach complementary to MD simulations^28,29^. Notably, AlphaFold3 models largely recapitulated the conformational changes and ligand-binding mode differences observed in the cryo-EM structures and MD simulations (Supplementary Fig. 18).

### Selectivity mechanisms in adrenergic, dopaminergic, and serotonergic GPCRs

To facilitate identification of residues determining selective responses to ligands, we developed a web-based resource for comparing groups of receptors (https://carlssonlabtools.icm.uu.se/GPCR_Selectivity_Explorer). This tool automated our analyses of the β-adrenergic and D_1_-like dopaminergic receptors to identify selectivity hotspots and can be used to compare any two sets of GPCRs. We extended our analysis of selectivity hotspots to other pairs of adrenergic, serotonergic, and dopaminergic receptors. We identified two groups of closely related receptors (group 1: β-adrenergic, D_1_-like dopaminergic, and 5-HT_6_ serotonergic receptors; group 2: α_2_-adrenergic, D_2_-like dopaminergic, and 5-HT_2_ serotonergic receptors). We then identified selectivity hotspots for the six pairs of receptors recognizing different native ligands using our approach. In total, 22 selectivity hotspot positions were identified, and a majority of these were located in the extracellular region of the receptors (Supplementary Fig. 19a). Four hotspot positions were located in the orthosteric binding site (Supplementary Fig. 19a). Binding site positions 3x36 and 7x38 emerged as the most frequently identified hotspots and were predicted to contribute to selectivity in five and three out of the six receptor pairs, respectively. Hotspot positions outside the binding site exhibited lower conservation. The two positions identified for the β-adrenergic and D_1_-like dopaminergic receptors (2x60 and 3x41) were only identified in one additional receptor pair. Instead, hotspots appeared in other regions, often in close proximity to conserved motifs involved in activation. For example, there was a single hotspot region in the TM1-TM2-TM7 interface (positions 1x46 and 7x43) for the α_2_-adrenergic and D_2_-like dopaminergic receptor pair (Supplementary Fig. 19b). Notably, previous studies have shown that mutations in position 1x46 (I48T) enhance the response of D_2_R to dopamine and serotonin^30^. These results suggest that this site may have a similar role to the TM3-TM4-TM5 site in the β_2_R, where mutations enhanced receptor response to ligands (Fig. 3c).

## Discussion

In this work, we identified molecular mechanisms governing receptor selectivity for native ligands by integrating sequence analysis, structural biology, and molecular pharmacology. Our approach identified regions responsible for catecholamine selectivity in β-adrenergic and D_1_-like dopaminergic receptors guided by sequence and structural data. Characterization of receptor mutants combined with molecular simulations led to three main findings. First, the selectivity profiles of the β_2_R and D_1_R could be swapped by exchanging a small number of residues between the receptors. Mutations in four to seven subfamily-specific residue positions switched receptor selectivity and maintained high agonist potency. Second, the regions responsible for controlling selectivity were located both within and outside the binding site. Within each receptor subfamily (D_1_-like dopaminergic and β-adrenergic), the same set of mutations swapped the selectivity profile of all human members. Finally, cryo-EM structures of receptor mutants combined with molecular simulations revealed mechanisms determining receptor selectivity.

We identified distinct mechanisms that act in concert to alter the selectivity profile of catecholamine-binding GPCRs. As anticipated, exchanging residues within the orthosteric site is required to modify the shape and polarity of the pocket, enabling accommodation of a different ligand. However, binding-site mutations primarily led to a loss of response to the native agonist, rather than switching the selectivity profiles and increasing the potency of the new ligand. Receptors with several binding site mutations typically exhibited weak responses and either remained selective for the native agonist or were non-selective. Our observations agree with alanine and deep-mutational scanning studies focusing on the β_2_R, which observed a loss of agonist activity for most binding site mutations^18,25,31–33^. These results suggest that the interaction network linking the orthosteric and G protein binding sites has evolved high selectivity for a certain agonist and even a single mutation can disrupt ligand binding or allosteric communication. One of our key findings is that mutations both within and outside the binding site need to be combined to switch the receptor selectivity profile. The regions outside the binding site involved networks of residues (2x60, 2x64, 3x41), which were not identified as important for receptor function by studies restricted to single mutations or alanine substitutions^25,31^. Two distinct mechanisms contributing to switching receptor selectivity were identified. The first of these involved residues in the TM3-TM4-TM5 interface and did not influence selectivity per se. In the β_2_R, this region connects the ligand binding site to microswitches that control conformational changes. Mutations increased the receptor response to agonist binding, resulting in potencies closer to physiological concentrations. The second mechanism involved residues in the TM2-TM7 interface surrounding the orthosteric ligand-binding pocket. A switch in the selectivity profile of D_1_R was only possible by exchanging residues located within the binding site combined with three mutations in this second region. Our MD simulations revealed that this interaction network positioned binding site residues optimally for ligand recognition. The fact that the same set of mutations swapped the selectivity of all receptors in the same subfamily suggested that the mechanism is conserved. Notably, the probability of selecting a set of seven specific residues by chance from the 100 positions that differ between the D_1_R and β_2_R would be around one in 16 billion, illustrating the power of our data-driven approach.

**Fig. 7:**
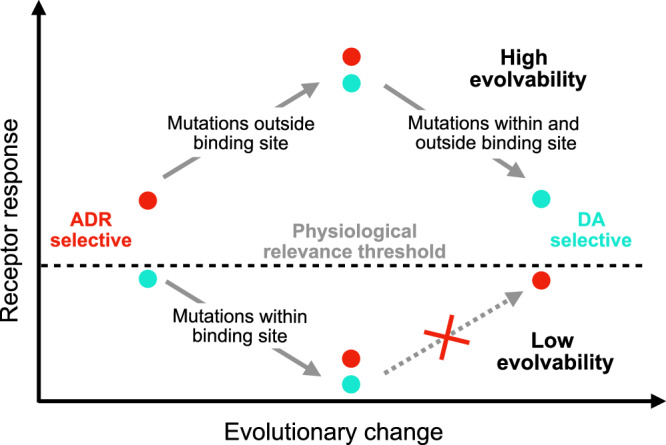
Model for the evolution of switched selectivity. The mechanism of altering the ligand preference (ADR vs. DA) in catecholamine GPCRs is likely initiated by mutations outside the binding site because changes within the orthosteric site tend to impair activity, resulting in poorly evolvable receptors. Residue positions outside the binding site can increase receptor response to a new ligand, leading to activity at physiologically relevant concentrations. Such receptors are promiscuous as they respond to the native and new ligand at physiological conditions, which will facilitate the acquisition of a new function under selection pressure.

Class A GPCRs share a common evolutionary origin, and the superfamily has expanded through a number of gene duplications, resulting in one of the largest families in vertebrate genomes. Following duplication, GPCR genes have accumulated mutations that have made the receptors selective for an exceptionally broad range of compounds^34^. However, the molecular mechanisms enabling the evolution of new receptor functions are not fully understood. Extensive studies on enzymes have demonstrated that new protein functions typically occur in a stepwise manner^35^. The process is often initiated by the development of a promiscuous state, in which the protein exhibits weak activity for the new function. Once this activity becomes relevant at physiological conditions, the new function can often evolve, driven by selection pressure^36^. Based on our results, the mechanism of altering the ligand preference in catecholamine GPCRs is likely initiated by mutations outside the binding site, as changes within the orthosteric site tend to impair receptor activity (Fig. 7). Our work, along with previous studies^30,37,38^, have identified residue positions outside the binding site that increase receptor response to a non-native ligand. Such receptors are, in principle, promiscuous as they respond to the native and new ligand at physiological conditions, which will facilitate the acquisition of a new function under selection pressure. Another important aspect is that altered function is facilitated by gene duplication, after which one copy may maintain the original function and the other copy can evolve new properties (neo-functionalization). It is essential that some degree of functionality is maintained in the copy that undergoes a transition to an altered function, otherwise it would be likely to end up as a pseudogene^39^. Our observations explain how receptor selectivity for ligands in this group has changed on multiple occasions, and the molecular mechanisms identified by our work may be a key contributor to the remarkable evolvability of the GPCR family.

The adrenergic and dopaminergic receptors are important therapeutic targets^40^, and one of the major challenges in drug discovery has been to develop target-selective compounds. Our findings can inform the design of selective orthosteric ligands and provide directions for identification of drugs with new mechanisms of action. The potential to target the identified selectivity hotspots in ligand design is supported by recently solved receptor structures. In the orthosteric site, the selective β-blocker carazolol forms hydrogen bonds with the hotspot residue N^7x38^ in the β_2_R^41^, and D_1_-like selective drug fenoldopam occupies the hotspot region located in the second extracellular loop of the receptors^33^. The other two selectivity hotspot regions are located outside the binding site, but were found to have a strong influence on ligand potency and efficacy. Recently solved receptor structures show that both of these pockets are promising targets for allosteric modulators. Remarkably, the negative allosteric modulator AS408 interacts with hotspot residue E^3x41^ in the identified TM3-TM4-TM5 pocket of the β_2_R^42^, and a cavity occupied by cholesterol was recently identified in TM2-TM7 hotspot region of the D_5_R (Supplementary Fig. 20)^25^. Notably, the allosteric pockets are less conserved than the orthosteric site, which will facilitate the design of selective drugs. As reflected by our work, MD simulations will be essential in drug design to understand how interaction networks influence the orthosteric binding site and the receptor response^24,42–47^.

In addition to providing insights into the general principles of how GPCRs evolved ligand selectivity and strategies for drug design, our results have several other potential application areas. Characterizing the residue interaction networks controlling ligand recognition and activation can provide insights into the mechanisms of disease-related mutations and differences in drug response due to genetic variation^48,49^. Our findings may also pave the way for engineering of receptor selectivity and signaling properties, a research field that our results indicate can be further accelerated by the recent advances in machine learning^29,50,51^. Such designer receptors will have transformative applications in biosensors^51^, chemogenetics, and gene therapy^52^. The openly accessible tools developed in our study could also be applied to understand the molecular mechanisms of selective ligand recognition in other protein families.

## Methods

### Cell culture and transfection

The CHO-K1 Chinese hamster ovary cell line was cultured at 37 °C in 5% CO_2_ in an air-ventilated humidified incubator, in Dulbecco’s Modified Eagle Medium/Nutrient Mixture F-12 Ham (Thermo Fisher Scientific, Waltham, MA, US), supplemented with 10% fetal calf serum (Thermo Fisher Scientific), 1% Glutamax (Thermo Fisher Scientific), 100 U/mL penicillin G (Sigma Chemical Co., St. Louis, MO, USA) and 80 U/mL streptomycin (Sigma). Cells were divided 2–3 times/week using detachment by trypsin-EDTA (Ethylene-diamine-tetraacetic acid) (0.25%, 0.02%) (Thermo Fisher Scientific). EDTA (0.02%) in phosphate-buffered saline (PBS) was used for detaching the cells prior to the assays in order to prevent receptor cleavage by trypsin. The cDNA of D_1_R and β_2_R wild-type and mutant variants were produced by GenScript (GenScript Biotech Corporation, Piscataway, NJ, US) in pcDNA3.1(+)-N-HA expression vector. The cells were transiently transfected 24–48 h prior to the experiments. For the transfection, the cells were grown to 60–80% confluency in antibiotic-free culture medium. TransIT-CHO (Mirus Bio, Madison, US) was used as a transfection reagent and used according to the manufacturer’s protocol. Receptor expression was verified by antiHA immunofluorescence staining of the N-terminal HA-tag in flow cytometry. Prior to the immunostaining, the cells were cultured on 6-well plates in antibiotic-free medium to 80% confluency. The cells were transfected as described above. After 24 h the cells were collected and prepared for immunostaining in suspension. Shortly, the cells were washed twice with PBS followed by fixation by 4% paraformaldehyde (PFA) in PBS (15 min at room temperature, occasional mixing). The cells were washed twice with PBS, and suspended in blocking buffer 1% bovine serum albumin (BSA) in PBS + 0.1% Tween 20 (Polyoxyethylene (20) sorbitan monolaurate), followed by 30 min incubation at room temperature. Cells were then incubated with the fluorescein-conjugated anti-HA-antibody, diluted in the blocking buffer at 2 μg/ml, and the suspension was incubated 1 h on a shaker in darkness. The cell suspension was washed three times with PBS, and the fluorescence (*λ*_ex_  =  488 nm, *λ*_em_  =  525/30 or 533/30 nm) was measured in flow cytometry (Guava® easyCyte™, Merck Millipore, Burlington, US, or BD Accuri™ C6, BD Biosciences, Frankin Lakes, US). Cells were not permeabilized in order to detect only the membrane expression. The data were then normalized to the wild-type receptor expression (100%) of each experiment before averaging. The measurement of expression was conducted for each expression construct in 3–4 batches of cells. The flow cytometry gating strategy is shown in Supplementary Fig. 21.

### cAMP assays

The intracellular cAMP elevation was measured with Homogeneous Time-Resolved Fluorescence (HTRF) Gs dynamic Kit (CisBio, PerkinElmer) in Chinese Hamster Ovary K1 (CHO-K1) cells. The cells were seeded on 12-well plates 48 h before the assay. On the next day, the cells were transiently transfected with wild-type or mutant receptor with TransIT-CHO (Mirus Biosciences) and 0,286 μg/cm^2^ DNA according to manufacturer protocol. After 24 h, the cells were collected and resuspended in the assay buffer. The assay buffer consisted of Hank’s Balanced Salt solution, supplemented with 20 mM 2-[4-(2-hydroxyethyl)piperazin-1-yl]ethane-1-sulfonic acid (HEPES), 0.1% bovine serum albumin and 500 μM isobutylmethylxanthine. Three thousand cells per well were plated on a white 96-low volume plate (CisBio, PerkinElmer), and stimulated with buffer only or increasing concentrations of adrenaline, noradrenaline or dopamine dissolved in the assay buffer. The cells were stimulated for 30 min at 37 °C, and then the reaction was stopped by adding the d2-labeled cAMP tracer (acceptor) in detection and lysis buffer, followed by the addition of the Europium cryptate labeled antibody (donor). The plates were incubated for 1 h at room temperature in darkened conditions. The fluorescence emission was then read with two wavelengths (665 nm and 620 nm) with FluoStar Omega plate reader. The HTRF signals were plotted on a standard curve to acquire the actual concentration of cAMP. The actual cAMP concentrations were normalized to wild-type D_1_R or β_2_R maximum response (100%). A local maximum response control was included in each experiment for each plate. The data were analyzed in GraphPad Prism9, using 3-parameter logistic function. The experiments were conducted with at least two batches of cells. The number of biological experiments for each mutant is provided in Supplementary Data 1.

### G protein recruitment assays

Agonist-mediated receptor activation was measured by bystander BRET using an engineered G protein (donor: Rluc8-mGs) that translocates from the cytosol to the plasma membrane (acceptor: rGFP-CAAX) to bind the receptor following its transition to an active conformation^53,54^. HEK293A cells were propagated in plastic flasks and grown at 37 °C in 5% CO_2_ and 90% humidity. Cells (3.5 × 10^5^ cells/ml) were transfected in suspension with plasmid DNA adjusted to 1 μg with salmon sperm DNA (Invitrogen), complexed with polyethylenimine (PEI) (MW 25,000, 3:1 PEI ratio), and seeded at 3.5 × 10^4^ cells/well in white 96-well plates precoated with PDL. After 48 h, the cells were washed with and maintained in HBSS. The cells were incubated with coelenterazine 400a (5 μM) for 5 min at 37 °C and then stimulated with agonist prior to Bioluminescence Resonance Energy Transfer (BRET) measurement. Plates were read on a Tecan Spark multimode microplate reader (Tecan, Männedorf, Switzerland) equipped with filters for BRET2 (center wavelength/bandwidth: 400/70 nm for donor and 515/20 nm for acceptor) for the detection of Renilla reniformis luciferase (RlucII) and Renilla reniformis green fluorescent protein (rGFP) emissions, respectively. Each well was recorded for 130 s after stimulation with agonist, and the maximum BRET change of each condition was used to obtain concentration-response curves. Plasmid DNA constructs Rluc8-mGs were from Nevin A. Lambert (Augusta University) and rGFP-CAAX (polybasic sequence with the prenylated CAAX box of the GTPase first identified in Kirsten Rat Sarcoma virus [KRAS]) was from Michel Bouvier (Université de Montréal). β_2_R was purchased from cDNA.org (Bloomsburg University, Bloomsburg, PA, USA). All plasmid constructs were verified by Sanger sequencing. Dulbecco’s modified Eagle’s medium (DMEM), Dulbecco’s phosphate-buffered saline (D-PBS), Poly-D-Lysine (PDL), Trypsin, PBS, penicillin/streptomycin, and fetal bovine serum were purchased from Gibco (Thermo Fisher Scientific, Waltham, MA, USA). PEI was from Alfa Aesar (Thermo Fisher Scientific, Waltham, MA, USA). Coelenterazine 400a was purchased from Nanolight Technologies (Pinetop, AZ, USA). Dopamine, adrenaline, and noradrenaline were sourced from Sigma-Aldrich (St. Louis, MO, USA). The data were normalized to wild-type D_1_R or β_2_R basal and maximum response (0–100%) before averaging, and analyzed in GraphPad Prism 9, using 3-parameter logistic function and presented as mean ± SEM. The experiments were conducted with 3 to 6 batches of cells.

### Expression and purification of the receptors

The β_2_R mutant (V117S/E122L/T123N/N312V, M16) and D_1_R mutant (K81G/E85I/S107V/A147V/D314I/V317N/W321Y, M74) were cloned into pcDNA3.1 vector with N-terminal hemagglutinin (HA) signal peptide followed by Flag tag and a C-terminal miniG_αsiN_ fusion protein. Expi293F cells (Thermo Fisher), cultured in SMM 293-TII-N expression medium (SinoBiological), were transfected at a density of 2.0 million cells/mL using linear 25 kDa MW polyethylenimine (Polysciences Inc) at a PEI:DNA ratio of 3:1 with expression vectors and harvested 48 h post-transfection. Nanobody 35 (Nb35) was subcloned into the pET26b vector with a C-terminal hexahistidine (6×His) tag. The plasmid was transformed into BL21(DE3) *E. coli* cells and cultured in Terrific Broth (TB) medium. Protein expression was induced with 1 mM isopropyl β-d-1-thiogalactopyranoside (IPTG) when the culture reached a density (OD600) of 0.8, followed by incubation for 18 h at 20 °C. Gβ_1γ2_ and scFv16 were subcloned into the pFastBac vector, and baculoviruses were generated using the Bac-to-Bac system. For protein expression, Trichoplusia ni (Hi Five) insect cells at a density of approximately 3.0–4.0×10^6^ cells/mL were infected with baculovirus and cultured for 48 h before harvest.

For the purification of Nb35, Gβ_1γ2_ and scFv16, Ni-affinity chromatography was employed according to established protocols^55,56^. Cell lysates (for Nb35 and Gβ_1γ2_) or culture supernatant (for scFv16) containing the target proteins were bound to Ni-NTA resin. After extensive washing with a buffer containing 20 mM imidazole to remove non-specifically bound impurities, the proteins of interest were eluted using a buffer supplemented with 200 mM imidazole. The eluted Nb35 and scFv16 were subsequently subjected to size exclusion chromatography (SEC) for further purification. The eluted Gβ_1γ2_ was dialyzed, cleaved with HRV 3 C protease, and then subjected to reverse Ni-NTA chromatography.

For the β_2_R(M16)-dopamine-miniG_αsiN_-Gβ_1γ2_-Nb35-scFv16 complex, β_2_R(M16)-expressing cell pellets from 1 L culture were lysed in 100 mL of buffer containing 10 mM HEPES, pH 7.5, 1 mM EDTA, 160 μg/mL benzamidine, 100 μg/mL leupeptin and 10 µM dopamine for 30 min at room temperature. Upon complete lysis, the membrane fraction was isolated by centrifugation and subsequently solubilized using 100 mL solubilization buffer (20 mM HEPES, pH 7.5, 100 mM NaCl, 1% dodecyl maltoside (Anatrace), 0.1% cholesterol hemisuccinate (CHS, Sigma), 160 μg/mL benzamidine, 100 μg/mL leupeptin, 2 mg/mL iodoacetamide, benzonase and 10 µM dopamine) at 4 °C for 2 h with stirring. The supernatant was supplemented with 2 mM CaCl_2_ and was loaded onto an M1-FLAG affinity column (Sigma). During this step, the detergent was gradually replaced by 0.01% LMNG (Lauryl maltose neopentyl glycol, Anatrace). The purified β_2_R mutant was concentrated and mixed with Gβ_1γ2_, Nb35, and scFv16 at a molar ratio of 1:1.2:1.2:1.2, followed by incubation on ice for 2 h. After incubation, the mixture was purified with Superdex 200 Increase 10/300 GL column equilibrated with a buffer containing 20 mM HEPES, pH 7.5, 100 mM NaCl, 0.001% (w/v) LMNG, 0.0002% CHS, and 10 µM dopamine. The purified complex was concentrated to ~10 mg/mL and aliquoted for further use.

For the D_1_R(M74)-adrenaline-miniG_αsiN_-Gβ_1γ2_-Nb35 complex, D_1_R(M74) expressing cell pellets from 1 L culture were lysed in 100 mL of buffer containing 10 mM HEPES, pH 7.5, 1 mM EDTA, 160 μg/mL benzamidine, 100 μg/mL leupeptin and 10 µM adrenaline for 30 min at room temperature. Upon complete lysis, the membrane fraction was isolated by centrifugation and subsequently solubilized using 50 mL solubilization buffer (20 mM HEPES, pH 7.5, 100 mM NaCl, 0.5% LMNG, 0.1% CHS, 160 μg/mL benzamidine, 100 μg/mL leupeptin, 2 mg/mL iodoacetamide, benzonase and 10 µM adrenaline) at 4 °C for 2 h with stirring. The supernatant was supplemented with 2 mM CaCl2 and was loaded to an M1-FLAG affinity column. The purified D_1_R mutant was concentrated and mixed with Gβ_1γ2_ and Nb35 at a molar ratio of 1:1.2:1.2, followed by incubation on ice for 2 h. After incubation, the mixture was purified with Superdex 200 Increase 10/300 GL column equilibrated with a buffer containing 20 mM HEPES, pH 7.5, 100 mM NaCl, 0.001% (w/v) LMNG, 0.0002% CHS, and the 10 µM adrenaline. The purified complex was concentrated to ~10 mg/mL and aliquoted for further use.

### Cryo-EM grid preparation

For cryo-EM grid preparation, 4 µL of purified receptor-G protein complexes were applied onto glow-discharged Au Quantifoil grids. The grids were subsequently blotted using Whatman No.1 qualitative filter paper in a Vitrobot Mark IV (Thermo Fisher) maintained at 8 °C and 100% humidity for 4 s with a blotting force of four. Following blotting, the grids were rapidly plunged into liquid ethane for vitrification.

### Data collection and processing

Cryo-EM data for the β_2_R(M16)-dopamine-Gβ_1_γ_2_-Nb35-scFv16 complex and D_1_R(M74)-adrenaline-Gβ_1_γ_2_-Nb35 complex were collected using a Titan Krios G3i TEM (ThermoFisher) and acquired through AutoEMation^57^. Data processing was performed by following a standard workflow using cryoSPARC (v4.5.1)^58^. Initially, multiple rounds of 2D classification were performed, and the best 2D averages were selected for subsequent 3D reconstruction. These selected 2D averages served as templates for particle picking across the entire dataset. A 3D volume displaying clear soluble features and a well-defined transmembrane domain was chosen as the template for further 3D classification. After several rounds of heterogeneous refinement and non-uniform refinement, a 3D volume with well-resolved secondary structures was obtained. The detergent micelles present in the 3D volume were removed to create a mask that specifically encapsulated the transmembrane domain, which was used for focused classification. Through iterative cycles of heterogeneous refinement and 3D classification, a high-resolution volume was achieved. The β_2_R(M16)-dopamine-Gβ_1_γ_2_-Nb35-scFv16 complex dataset comprising 230,232 particles, and the D_1_R(M74)-adrenaline-Gβ_1_γ_2_-Nb35 complex dataset comprising 231,507 particles, were both utilized for the final structure reconstruction.

### Model building and refinement

The cryo-EM structures of the β_2_R-G_s_ complex (PDB accession code: 3SN6^59^) and the MC1R complex (PDB accession code: 7KH0^56^) were utilized as initial structural models for the receptor and other components of the β_2_R(M16)-dopamine-Gβ_1_γ_2_-Nb35-scFv16 complex, while the cryo-EM structure of the D_1_R complex (PDB accession code: 7F0T^60^) served as the initial structural model for the D_1_R(M74)-adrenaline--Gβ_1_γ_2_-Nb35 complex. Coordinates and chemical constraints for dopamine and adrenaline were generated using Phenix.elbow (1.20.1-4487^61^). The models were fitted into the cryo-EM maps using UCSF ChimeraX-1.3^62^, followed by manual adjustments with COOT (0.9.8.7^63^). Structural refinement and validation were performed using PHENIX^64^.

### UMAP analysis of sequences

A set of 83 GPCR sequences in FASTA format was retrieved from GPCRdb^65^, which included adrenergic (α_1_, α_2_, and β) and dopaminergic (D_1_- and D_2_-like) receptors. To assess if differences captured by the UMAP analysis were conserved across species, sequences from 16 different organisms were included (UniProt codes: bovin, canlf, caphi, cavpo, chlae, felca, human, macmu, mesau, mouse, muspf, pantr, pig, rabit, rat, and sheep). Sequence alignments were performed using GPCRdb for both the full receptor sequences and the orthosteric binding pocket (amine and major binding pocket, positions: 3x32, 3x36, 6x48, 6x51, 7x38, 7x42, 3x33, 3x37, 3x40, 4x56, 4x57, 5x39, 5x40, 5x43, 5x44, 5x461, 5x47, 6x44, 6x52, 6x55, and 6x56)^17^. One-hot encoding was performed by converting each residue in the multiple sequence alignment into a binary vector of length 21, representing the 20 standard amino acids plus the gap character “–”. Dimensionality reduction was applied using UMAP^66^ to visualize the relationship between the receptor subfamilies with the resulting 2D projection.

### Structure-based sequence alignment

Sets of 515 β-adrenergic (β_1-3_) and 401 D_1_-like dopaminergic (D_1,5_) receptor sequences from various species were obtained from the Reference sequence (RefSeq) database at NCBI^67^. Sequences were aligned to the structurally conserved regions of wild-type sequences of β_2_R and D_1_R from GPCRdb^65^ using Jalview^68^. The consensus sequences for β-adrenergic and D_1_-like dopaminergic receptors were scored according to the BLOSUM62 substitution matrix using the package Biopython^69^. Positions with >80% conservation and BLOSUM62 substitution score <0 were considered as potential selectivity hotspots. Selection of mutants was guided by the identified selectivity hotspots combined with comparisons of β₂R and D₁R structures, with particular focus on the extracellular region and ligand binding site (defined as the residues within 4 Å of the native ligands in experimental structures^16,18^, positions: 3x28, 3x32, 3x33, 3x36, 45x52, 45x52 + 2, 5x43, 5x461, 6x51, 6x52, 6x55, 7x38, 7x42). In the analysis of the α_2_-adrenergic, D_2_-like dopaminergic, and serotonergic (5-HT_2_ and 5-HT_6_) receptors, sequence alignments were retrieved from GPCRdb^65^. This set contained 339 α_2A-C_-adrenergic receptor sequences, 445 dopaminergic receptor sequences, 351 5-HT_2_ serotonergic receptor sequences, and 92 5-HT_6_ serotonergic receptor sequences. In these calculations, the same binding site definition as in the UMAP analysis (amine and major binding pocket) was used^17^.

### Molecular dynamics simulations

To study receptor-ligand interactions, we performed MD simulations with explicit representation of the receptor, ligand, membrane, and solvent. Two crystal structures of the β_2_R (PDB accession codes: 4LDO^16^ and 2RH1^41^) and one cryo-EM structure of the D_1_R (PDB accession code: 7LJD^18^) were used as initial coordinates for the simulations. The 4LDO and 7LJD structures were the most suitable starting points for the simulations because they represent the receptors in active-like conformations with their native ligands bound. In the active β_2_R structure, thermostabilizing mutations were reverted and the nanobody was removed. For the active D_1_R structure, the G protein and nanobody were removed, and the missing part of EL2 was modeled using PRIME. Missing side chains were built with the Protein Preparation Wizard in Schrödinger 2021^70^. Mutants of the β_2_R (V117S/N312V, M4; E122L/T123N, M24; V117S/E122L/T123N/N312V, M16) and D_1_R (V317N/S107V/W321Y, M14; A147V/K81G/E85I/D314I, M85; V317N/S107V/W321Y/A147V/K81G/E85I/D314I, M74) were modeled using Maestro (Schrödinger Suite 2021). Protonation states of titratable residues were assigned at pH 7.0 using Maestro. For each simulation, all input files were generated via CHARMM-GUI^71^ with the CHARMM36m force field^72^. The protein was embedded into a POPC membrane bilayer containing 140 lipids and solvated with a TIP3P water box containing 0.15 M NaCl. The system was minimized and equilibrated under gradually decreasing constraints (2000 to 0 kJ/mol/Å) in the NPT ensemble at 300 K, following the default protocol from CHARMM-GUI. Subsequently, three independent 500 ns production simulations were performed for each system. All simulations were executed using GROMACS^73^ v2022.2. Initial and final coordinate files for the simulations are provided in Supplementary Data 2, and a summary of each system setup is included in Supplementary Table 4.

### Binding free energy calculations

The active β_2_R structure (PDB accession code: 4LDO) was prepared as described previously^24^. In the case of D_1_R (PDB accession code: 7LJD^18^), the G protein and nanobody were removed. Missing side chains were added using the Protein Preparation Workflow in Maestro (Schrödinger, LLC), and EL2 was modeled using PRIME in Maestro (Schrödinger, LLC). A set of 100 snapshots with diverse EL2 conformations was evaluated and we selected a starting structure that reproduced the relative binding free energies for dopamine, noradrenaline, and adrenaline to the wild-type D_1_R. This model of the D_1_R was then used to calculate the relative binding free energies for D_1_R mutants. Models of the mutant receptors were generated using the mutagenesis wizard tool in PyMOL (Version 2.5.0, Schrödinger, LLC). The ligands were prepared using LigPrep in Maestro Schrödinger, LLC. OPLS2005 and the force field parameters were generated using ffld_server^74^. Free energy perturbation calculations were then performed using the software Q^75^ and its OPLS all-atom force field version. The calculations were performed under spherical boundary conditions with a sphere radius of 25 Å centered on the ligand. Atoms outside the sphere were excluded from non-bonded interactions. Ionizable residues close to the sphere edge were set to their neutral form and atoms within 5 Å of the sphere edge were restrained to their initial coordinates. Water molecules at the sphere border were subject to radial and polarization restraints according to the surface-constrained all-atom solvent (SCAAS) model^76^. Solvent bonds and angles were constrained using the SHAKE algorithm^77^. A cutoff of 10 Å was used for non-bonded interactions except for ligand atoms, for which no cutoff was applied. Long-range electrostatic interactions were treated using the local reaction field approximation. In all simulations, a time step of 1 fs was used and non-bonded pair lists were updated every 25 steps. Binding free energies were calculated by transforming one ligand into another in the receptor and in aqueous solution using a single topology protocol that gradually transforms one ligand into another in a series of 93 intermediate states with five replicas per window. The transformations were divided into three major steps: (A) Transformation of partial charges, (B) introduction of soft-core potentials on atoms to be annihilated, (C) transformation of Lennard-Jones potentials and bonded terms. The three steps were performed with 31, 11, and 51 intermediate states, respectively. At each intermediate state, the receptor-ligand complexes were first equilibrated for 1 ns. During the equilibration, harmonic positional restraints on solute-heavy atoms were gradually released and the system was heated to the target temperature (298 K). This was followed by a 1 ns production phase and energy was collected every 25 fs. The Qfep software^75^ was used to calculate the free energy difference using Bennet’s acceptance ratio (BAR)^78^. The convergence of the free energy calculations is shown in Supplementary Fig. 22. The free energies were calculated from three independent simulations for each system. Initial and final coordinate files for the calculations are provided in Supplementary Data 2, and a summary of each system setup is included in Supplementary Table 4.

### Calculation of free energy landscapes

To explore free energy landscapes of receptor activation for the wild-type β_2_R and the mutant M16 (V117S/E122L/T123N/N312V), we applied the string method with swarms of trajectories, an enhanced sampling technique used to capture the most probable transition pathways between stable states in high-dimensional space supported by collective variables (CVs^47^). As outlined in prior work^79^, steered MD simulations were conducted with extra bias on 41 CVs to obtain an initial transition path consisting of a set of configurational points derived from the equilibrated systems, as described in the MD simulations section. The string method was then employed to iteratively refine the path with fixed start (active) and end (inactive) states. Each iteration followed three steps: (1) a 30-ps restrained equilibrium simulation with 10,000 kJ/nm^2^ harmonic potential at each point; (2) 32 independent 10-ps unbiased simulations to compute the average drift in CV space, and (3) reparameterization of the string based on updated CV coordinates. In total, 400 iterations, were performed for each system, aggregating over 2 μs of simulation time, until string convergence was achieved in which no substantial fluctuation at each point in the subsequent iterations was observed (Supplementary Fig. 15). Two-dimensional free energy landscapes were estimated using a maximum likelihood Markov State Model (MSM) built in Deeptime Python package^80^. Trajectories from converged string iterations were used to calculate a transition matrix. Time-lagged independent component analysis (tICA) and k-means clustering were employed for dimensionality reduction and discretization, respectively. Two hallmarks of β_2_R activation (the TM5 bulge and ionic lock distance) were chosen to construct MSMs, capturing the conformational dynamics of β_2_R under different conditions. Initial and final coordinate files for the simulations are provided in Supplementary Data 2, and a summary of each system setup is included in Supplementary Table 4.

### Analysis of MD trajectories

Hydrogen bond interactions during MD simulations were analyzed using GetContacts (https://getcontacts.github.io/). The tool identifies hydrogen bonds based on geometric criteria: a donor-hydrogen-acceptor angle cutoff of 120° and a donor-acceptor distance of less than 3.5 Å. Hydrogen bond occupancies were computed as the fraction of frames in which a hydrogen bond was detected throughout the simulation. The rotameric distribution of tryptophan residues was analyzed using the MDAnalysis library^81^. The rotamer state was defined by the *χ*_1_ (N-Cα-Cβ-Cγ) dihedral angle. To assess the statistical significance of differences in hydrogen bond occupancies across different systems, Welch’s t-test was conducted using *ttest_ind* function from the SciPy package^82^.

### Network analysis

The dynamic network analysis was performed using the NetworkView plugin in VMD^83,84^. The analysis included dopamine-bound β_2_R mutants (M4, M24, and M16), adrenaline-bound D_1_R mutants (M14, M85, and M74), and the wild-type receptors (β_2_R-adrenaline and D_1_R-dopamine complexes). For each system, representative snapshots from simulations based on the active receptor were extracted from MD trajectories. In the network analysis, protein residues were defined as nodes, and edges were assigned between pairs of residues if any heavy-atom contacts were observed within 4.5 Å for at least 75% of the trajectory. Edge weights were calculated based on interaction frequencies derived from the dynamic cross-correlation matrix. Each network was then divided into communities of nodes with highly frequent and strongly connected interactions using Girvan–Newman algorithm^85^. For the β_2_R simulations, the residue 5x461 was defined as the source node, and positions 3x41 and 3x42 were defined as sink nodes. For the D_1_R simulations, positions 2x60, 2x64, and 7x35 were defined as source nodes, and position 7x42 was defined as the sink node. The Floyd–Warshall algorithm was applied to identify the shortest communication paths between the source and sink nodes^86^.

### AlphaFold3 models

Models of human wild-type and mutant β_2_R and D_1_R receptors in complex with G_s_ protein and ligands were generated using AlphaFold3 version 3.0.0^29^. Amino acid sequences were retrieved from the UniProt database (accession numbers: P07550, P21728, P04896, P54311, and Q5R7U4), whereas the ligands (dopamine, adrenaline, and noradrenaline) were specified using SMILES representations. The N- and C-termini of the receptors were omitted and template-free modeling was employed. The top five models ranked by the predicted local distance difference test (pLDDT) scores were selected for further analysis. The pLDDT and predicted aligned error (PAE) scores for the top models of each complex are shown in Supplementary Fig. 23.

### Reporting summary

Further information on research design is available in the Nature Portfolio Reporting Summary linked to this article.

## Supplementary information

Supplementary Information

Description of Additional Supplementary Files

Supplementary Data 1

Supplementary Data 2

Reporting Summary

Transparent Peer Review file

## Source data

Source Data 1

Source Data 2

## Data Availability

The data supporting the findings of this study are available within the article, its Supplementary Information, and the Source Data file. The structural models and associated datasets generated in this work have been deposited in the Protein Data Bank (PDB) under accession codes 9LW5 and 9LWC, and in the Electron Microscopy Data Bank (EMDB) under accession codes EMD-63431 and EMD-63440. Source data are provided with this paper.

## Code availability

The GPCR Selectivity Explorer server is accessible at https://carlssonlabtools.icm.uu.se/GPCR_Selectivity_Explorer. The source code is freely available on GitHub [https://github.com/Carlssonlab/GPCR_Selectivity_Explorer] and archived at Zenodo [10.5281/zenodo.18589679].
