# Supplementary material for: Molecular mechanisms of native ligand selectivity in catecholamine G protein-coupled receptors
Source: Nat Commun. 2026 Apr 23;17:4112. doi: 10.1038/s41467-026-71361-8 (PMC13150006; doi:10.1038/s41467-026-71361-8)
Supplement: Supplementary file 1 — Supplementary Information [file 41467_2026_71361_MOESM1_ESM.pdf]

Supplementary Information:

Molecular mechanisms of native ligand selectivity in catecholamine G protein-coupled receptors

Nour Aldin Kahlous[1,†], Maiju K. Rinne[2,3,†], Xin Zhang[4,,5†], Yanying Li[2], Yue Chen[6], Aikaterini Motso[7,8], Kaixuan Gao[4,5], Christina Bergqvist[2], Hongda Sheng[7,9,10], Yi Wang[9,10,11], Israel Cabeza de Vaca[1], Alejandro Díaz-Holguín[1], Philip Ullmann[1], Tore Bengtsson[8], Volker M. Lauschke[7,12,13,14,15], Jyrki P. Kukkonen[3], Lucie Delemotte[6], Shane C. Wright[7,12], Xiangyu Liu[4,5,*], Dan Larhammar[2,*], Jens Carlsson[1,*]

[1] Science for Life Laboratory, Department of Cell and Molecular Biology, Uppsala University, 75124 Uppsala. Sweden.

[2] Department of Medical Cell Biology, Uppsala University, SE-75123 Uppsala, Sweden.

[3] Department of Pharmacology, Medicum, University of Helsinki, POB 63, 00014, Helsinki, Finland.

[4] State Key Laboratory of Membrane Biology, Tsinghua-Peking Center for Life Sciences, Tsinghua University, Beijing, China.

[5] Beijing Frontier Research Center for Biological Structure, School of Pharmaceutical Sciences, Tsinghua University, Beijing, China.

[6] Science for Life Laboratory, Department of Applied Physics, KTH Royal Institute of Technology, SE-121 21, Stockholm, Sweden.

[7] Department of Physiology and Pharmacology, Karolinska Institutet, Stockholm, Sweden.

[8] Department of Molecular Biosciences, The Wenner-Gren Institute, Stockholm University, Stockholm, Sweden.

[9] College of Pharmaceutical Sciences, Zhejiang University, Hangzhou, 310058, China.

[10] Innovation Institute for Artificial Intelligence in Medicine of Zhejiang University, Hangzhou, 310020, China.

[11] National Key Laboratory of Chinese Medicine Modernization, Innovation Center of Yangtze River Delta, Zhejiang University, Jiaxing, 314100, China.

[12] Center for Molecular Medicine, Karolinska Institutet and University Hospital, Stockholm, Sweden.

[13] Dr Margarete Fischer-Bosch Institute of Clinical Pharmacology, Stuttgart, Germany.

[14] University of Tübingen, Tübingen, Germany.

[15] Department of Pharmacy, the Second Xiangya Hospital, Central South University, Changsha, China.

[†] These authors contributed equally
* Corresponding authors: jens.carlsson@icm.uu.se, dan.larhammar@uu.se, and liu_xy@mail.tsinghua.edu.cn

Table of Contents

Supplementary Figures

Supplementary Fig. 1. UMAP visualization of catecholamine receptor sequences 4
Supplementary Fig. 2. G protein recruitment assays for β_2R and D_1R 5
Supplementary Fig. 3. Snake plots of mutated positions in β_2R and D_1R 6
Supplementary Fig. 4. Relative cell surface expression of key mutants 7
Supplementary Fig. 5. Calculated relative binding affinities for binding site mutants 8
Supplementary Fig. 6. cAMP concentration-response curves for control mutants 9
Supplementary Fig. 7. cAMP response of β_2R and D_1R mutants to other biogenic amines 10
Supplementary Fig. 8. cAMP concentration-response curves of wild-type β_1R, β_3R, and D_5R 11
Supplementary Fig. 9. The cryo-EM data processing of the active β_2R-M16 mutant in complex with dopamine 12
Supplementary Fig. 10. The cryo-EM data processing of the active D_1R-M74 mutant in complex with adrenaline 13
Supplementary Fig. 11. Activation microswitches in wild-type and mutant receptors 14
Supplementary Fig. 12. Comparison of MD simulations snapshots with experimental structures 15
Supplementary Fig. 13. Analysis of the ligand binding mode in MD simulations of wild-type and mutant receptors 16
Supplementary Fig. 14. Analysis of residues 7x38 and 6x48 in MD simulations of wild-type and mutant receptors 17
Supplementary Fig. 15. Enhanced sampling MD simulations 18
Supplementary Fig. 16. Analysis of hydrogen bonding networks and the side chain rotamer of position 7x39 in MD simulations of wild-type and mutant receptors 19
Supplementary Fig. 17. Network analysis using β_2R and D_1R simulations 20
Supplementary Fig. 18. Comparison of AlphaFold3 (AF3) models with experimental structures 21
Supplementary Fig. 19. Selectivity hotspots in adrenergic, dopaminergic, and serotonergic GPCRs 22
Supplementary Fig. 20. Allosteric sites in experimental β_2R and D_1R structures 23
Supplementary Fig. 21. Flow cytometry gating strategy 24
Supplementary Fig. 22. Convergence of free energy estimates from FEP calculations 25

Supplementary Fig. 23. Confidence metrics of AlphaFold3 predicted complexes 26

Supplementary Tables

Supplementary Table 1. Identified selectivity hotspots and their conservation, BLOSUM62 scores, and location 27
Supplementary Table 2. Mutated positions and rationale for their selection 28
Supplementary Table 3. Cryo-EM data collection, refinement, and validation statistics 29
Supplementary Table 4. Setup of MD and FEP calculations 30

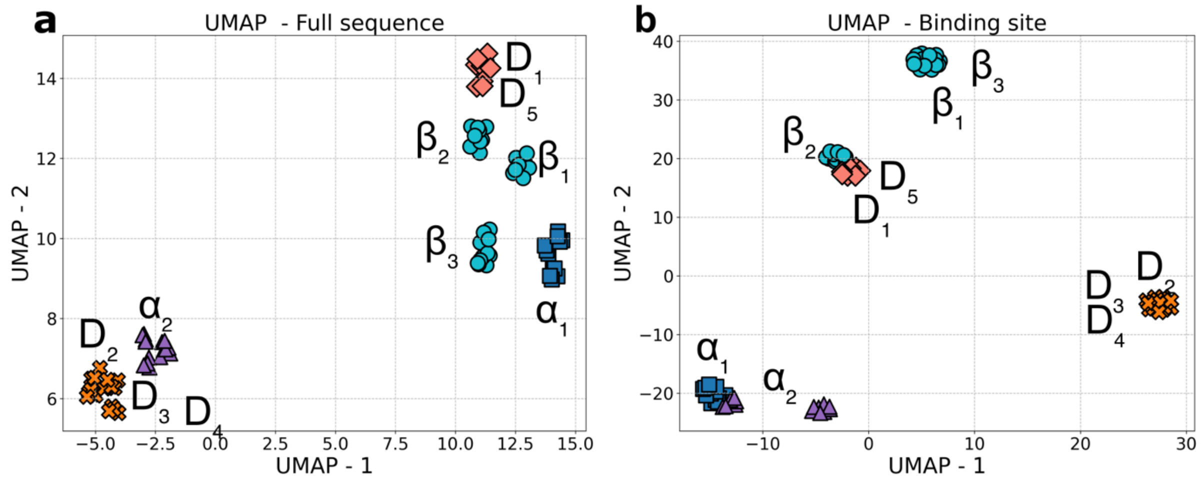

Supplementary Fig. 1. UMAP visualization of catecholamine receptor sequences. a UMAP embedding of full receptor sequences transformed using one-hot encoding of amino acids. **b** UMAP embedding of the aminergic binding pocket sequences transformed using one-hot encoding of amino acids. Points represent receptor sequences, color-coded by subtype (*e.g.*, α_1, α_2, and β for adrenergic and D_1- and D_2-like for dopaminergic receptors), with receptor families distinguished by markers.

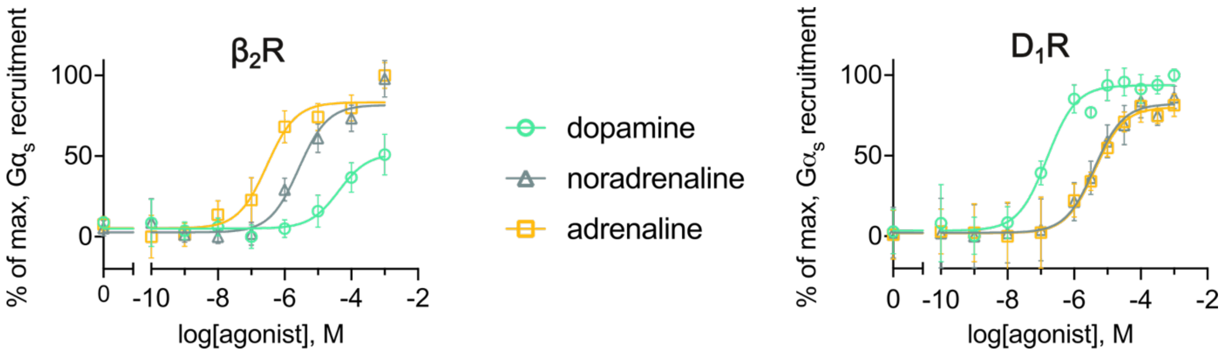

Supplementary Fig. 2. G protein recruitment assays for β_2R and D_1R. G protein recruitment assays for the β_2R and D_1R. Data from BRET assays are presented as mean ± SEM from at least three independent experiments and normalized to the maximal response of adrenaline in β_2R or dopamine in D_1R. Source data are provided as a Source Data file.

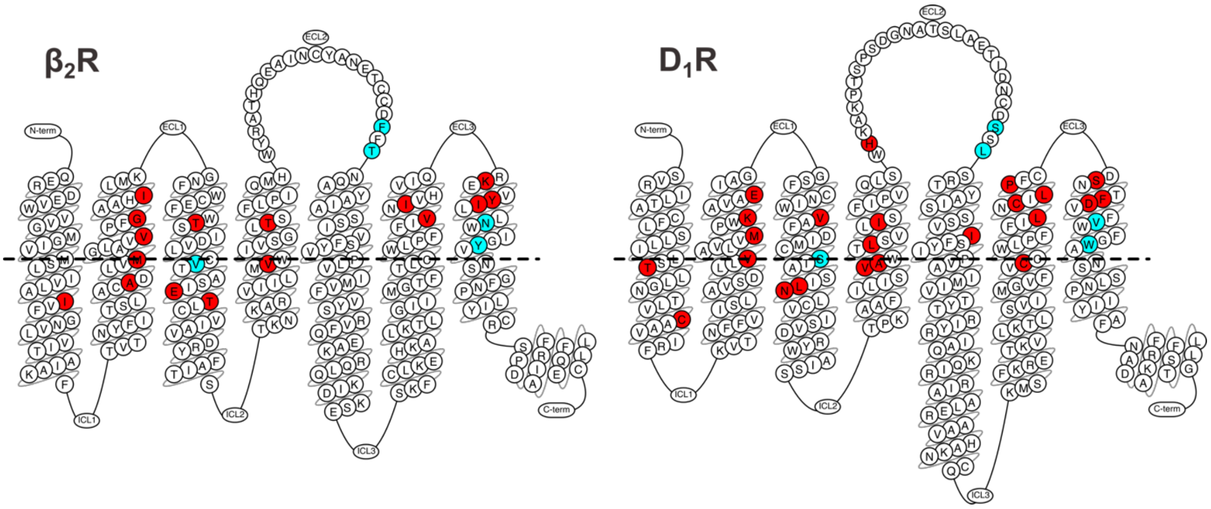

Supplementary Fig. 3. Snake plots of mutated positions in β_2R and D$_1$R. Positions within and outside the orthosteric binding site are colored in cyan and red, respectively. The dashed black lines indicate the midpoint of the transmembrane region.

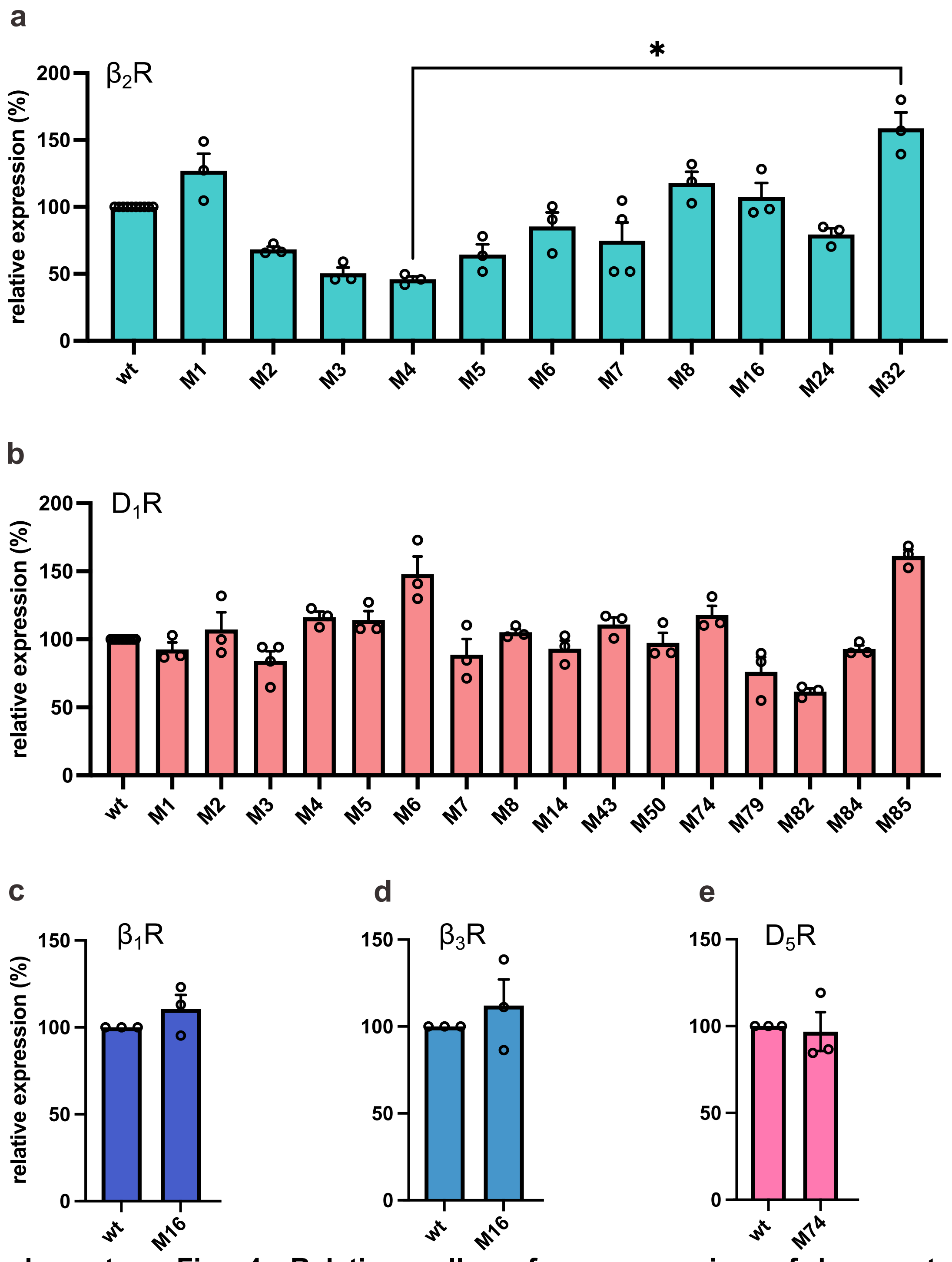

Supplementary Fig. 4. Relative cell surface expression of key mutants. Expression levels of WT and mutant variants of **a** β_2R, **b** D_1R, **c** β_1R, **d** β_3R, and **e** D_5R. Data are presented as mean ± SEM from at least three independent experiments. The expression is relative to the respective WT receptor. Statistical significance was assessed using the Kruskal-Wallis test with Dunn's multiple comparisons test. Only statistically significant comparisons are indicated (*$p < 0.05$). Source data are provided as a Source Data file.

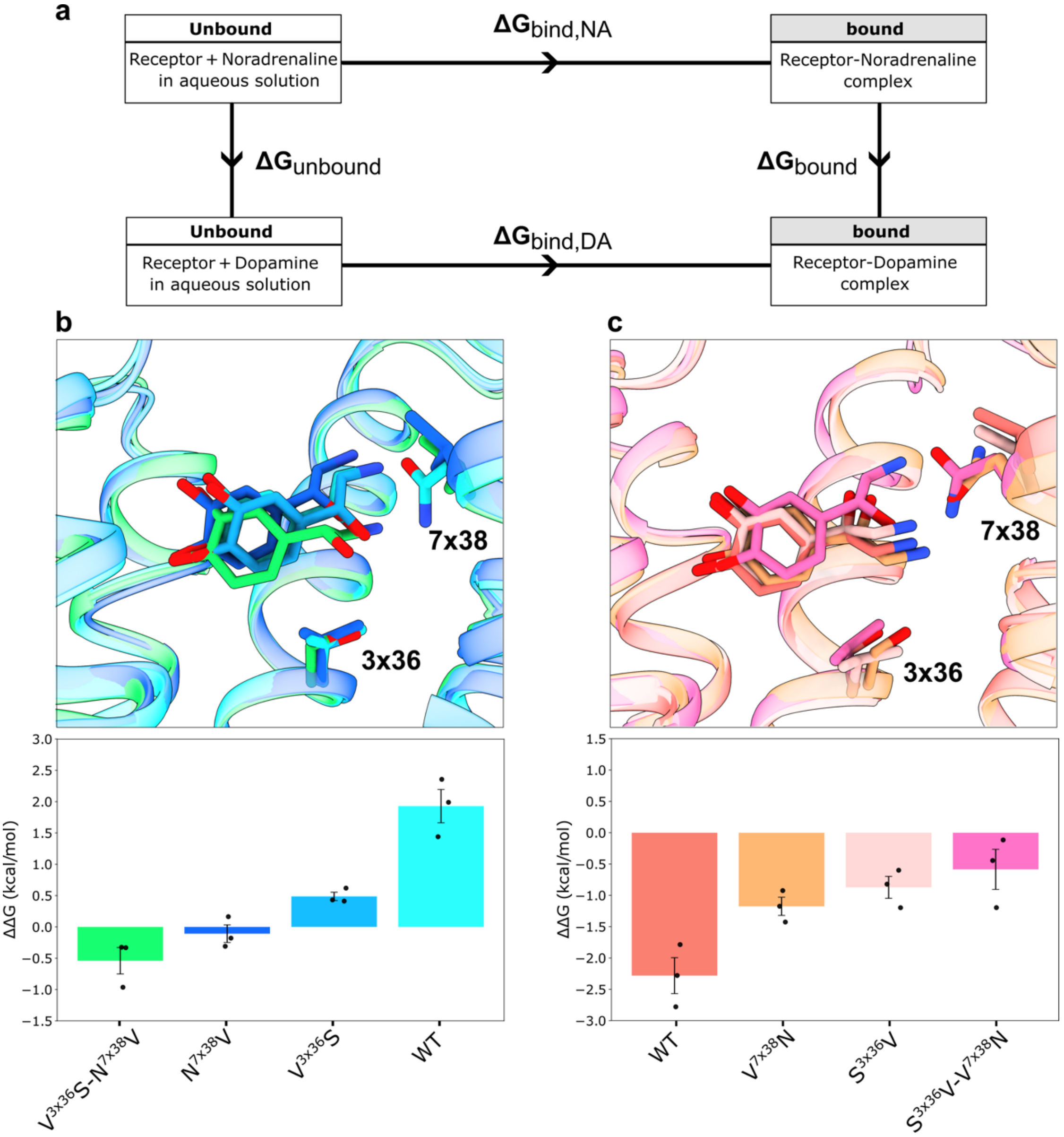

Supplementary Fig. 5. Calculated relative binding affinities for binding site mutants. a Thermodynamic cycle for calculation of the difference in binding free energy between dopamine (DA) and noradrenaline (NA). The relative binding free energy was calculated using MD/FEP by transforming noradrenaline to dopamine in aqueous solution ($\Delta G_{unbound}$) and in the complex with the receptor (ΔG_{bound}). The relative binding free energy ($\Delta\Delta G_{bind}$) can then be calculated as: $\Delta\Delta G_{bind} = \Delta G_{bind,DA} - \Delta G_{bind,NA} = \Delta G_{bound} - \Delta G_{unbound}$. **b** Representative snapshots of receptor-noradrenaline complexes obtained from MD/FEP calculations for the β_2R-WT and three mutant receptors, together with the corresponding calculated relative binding free energies. **c** Representative snapshots of receptor-noradrenaline complexes obtained from MD/FEP calculations for the D_1R-WT and three mutant receptors, together with the corresponding calculated relative binding free energies. In **b-c**, the receptor-noradrenaline complexes and the corresponding bar plots for each receptor variant are color-matched. Noradrenaline is shown as sticks and the receptor is depicted as a cartoon. Side chains of the mutated residues are shown as sticks and labeled with generic numbers. The $\Delta\Delta G_{bind}$ values represent averages of three independent free energy calculations, and error bars represent the SEM. Source data are provided as a Source Data file.

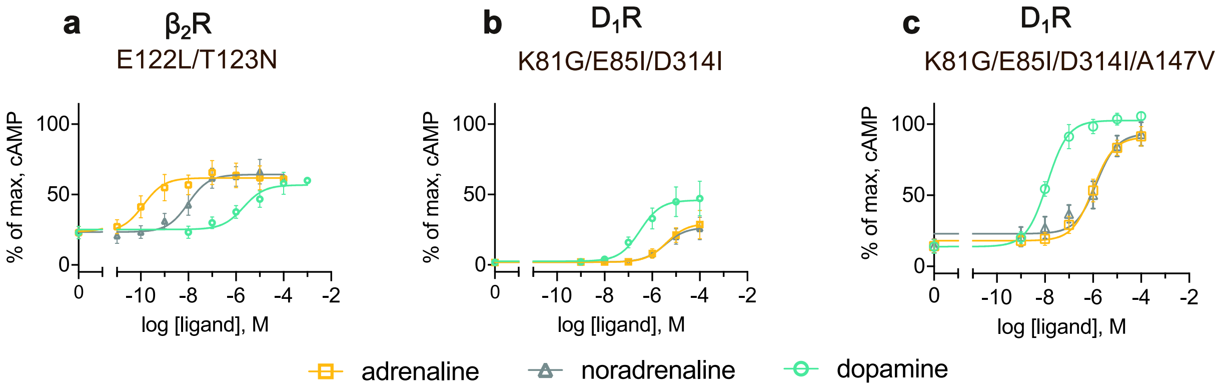

Supplementary Fig. 6. cAMP concentration-response curves for control mutants. a Mutations in the TM3-TM4-TM5 interface of the β2R (E122L/T123N, M24). **b** Mutations in the TM2-TM7 interface of the D1R (K81G/E85I/D314I, M82). **c** The combination of the mutation A147V in the TM3-TM4-TM5 interface and three mutations in the TM2-TM7 interface of D1R (K81G/E85I/D314I/A147V, M85). The E122L/T123N mutation led to increased basal activity in the cAMP experiments. Data were normalized to maximum response of corresponding WT receptor, and are presented as mean ± SEM from at least three independent experiments. Source data are provided as a Source Data file.

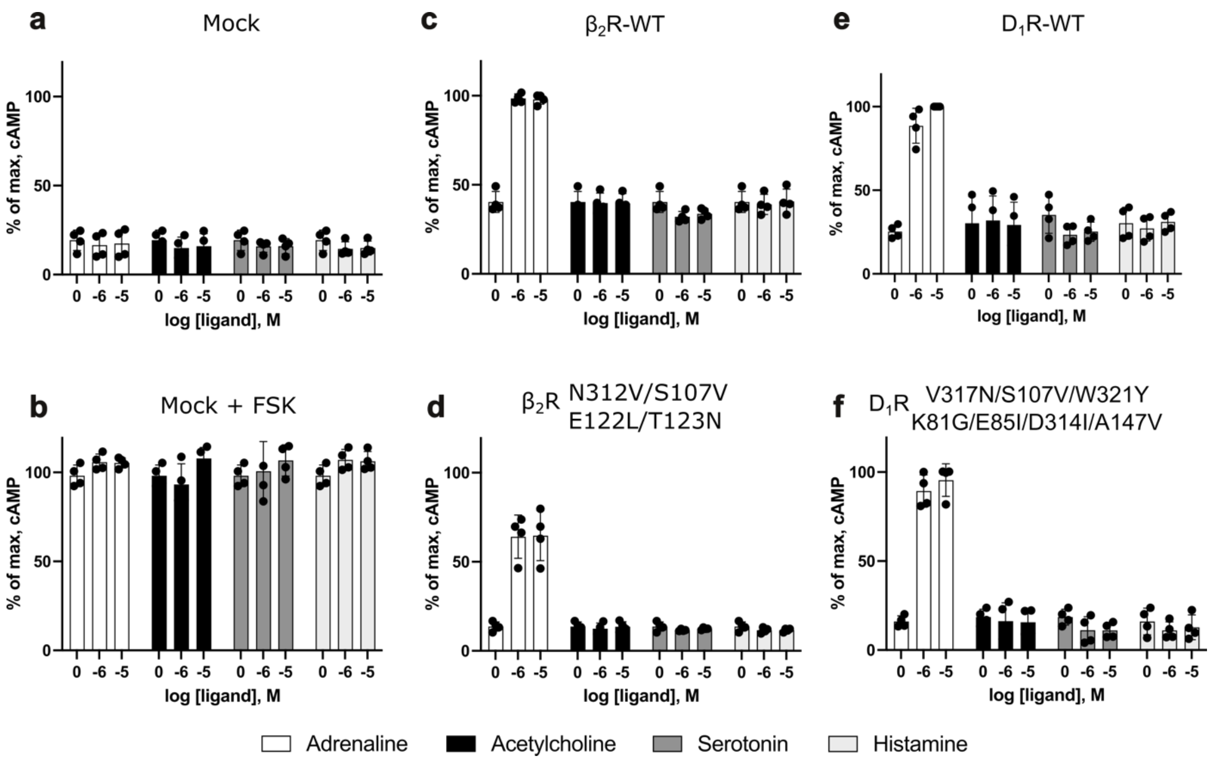

Supplementary Fig. 7. cAMP response of β$_2$R and D$_1$R mutants to other biogenic amines. Activation of receptor mutants by biogenic amines adrenaline, acetylcholine, serotonin, and histamine. **a** and **b** cAMP assay results for mock CHO cells without and with forskolin, respectively. **c** and **d** cAMP assay results for β$_2$R-WT and β$_2$R-M16, respectively. **e** and **f** cAMP assay results for D$_1$R-WT and D$_1$R-M74, respectively. Data are presented as mean ± SEM from at least three independent experiments. Before averaging, all data were normalized to the maximal response of adrenaline in β$_2$R-WT or D$_1$R-WT receptors. Source data are provided as a Source Data file.

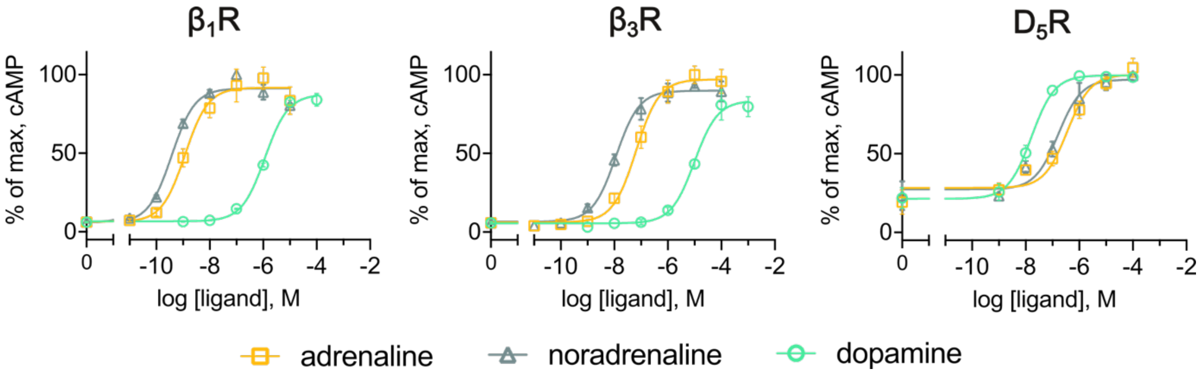

Supplementary Fig. 8. cAMP concentration-response curves of wild-type β_1R, β_3R, and D_5R. The pEC_{50} values of adrenaline and noradrenaline at the β_1R were 8.92 and 9.42, respectively, with 980- and 3000-fold selectivity over dopamine (pEC_{50} = 5.93), respectively. In the case of β_3R, the pEC_{50} values of adrenaline and noradrenaline at the β_1R were 7.18 and 7.93, respectively, with a selectivity of 150- and 850-fold over dopamine (pEC_{50} = 5.00). At the D_5R, the pEC_{50} value of dopamine is 7.79, with a selectivity of 24-fold over adrenaline (pEC_{50} = 6.40) and 10-fold over noradrenaline (pEC_{50} = 6.80). Data are presented as mean ± SEM from at least three independent experiments and normalized to the maximal response of noradrenaline (β_1R and β_3R) or dopamine (D_5R). Source data are provided as a Source Data file.

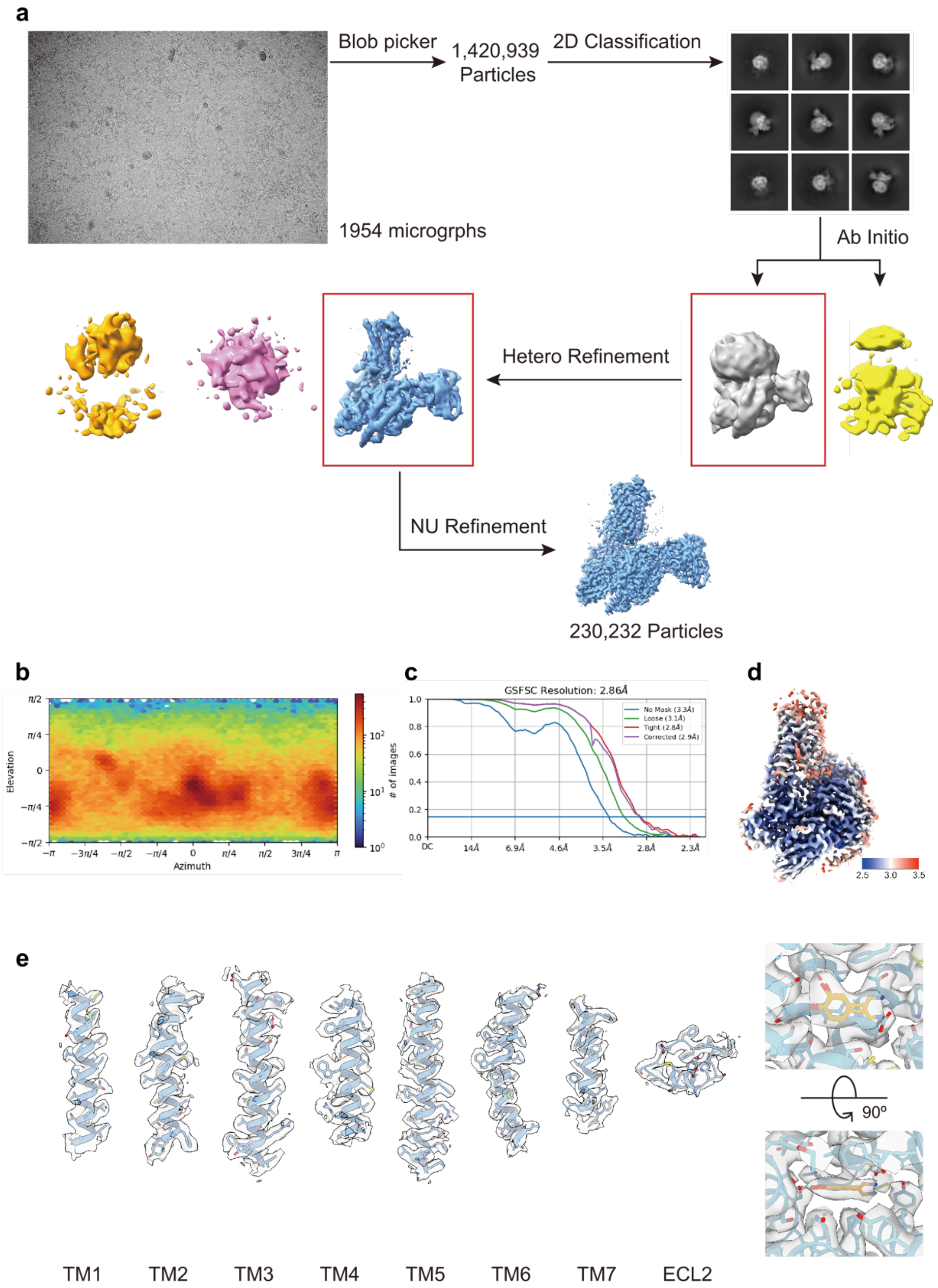

Supplementary Fig. 9. The cryo-EM data processing of the active β_2R-M16 mutant in complex with dopamine. The cryo-EM data processing workflow: **a** Viewing direction distribution, **b** FSC plot, **c** Local resolution, and **d** for the β_2R-M16-miniGsiN-Gβ1γ2-Nb35-scFv16-dopamine complex. **e** Cryo-EM density map of individual β_2R-M16 receptor helices, extracellular loop 2 (ECL2) and dopamine.

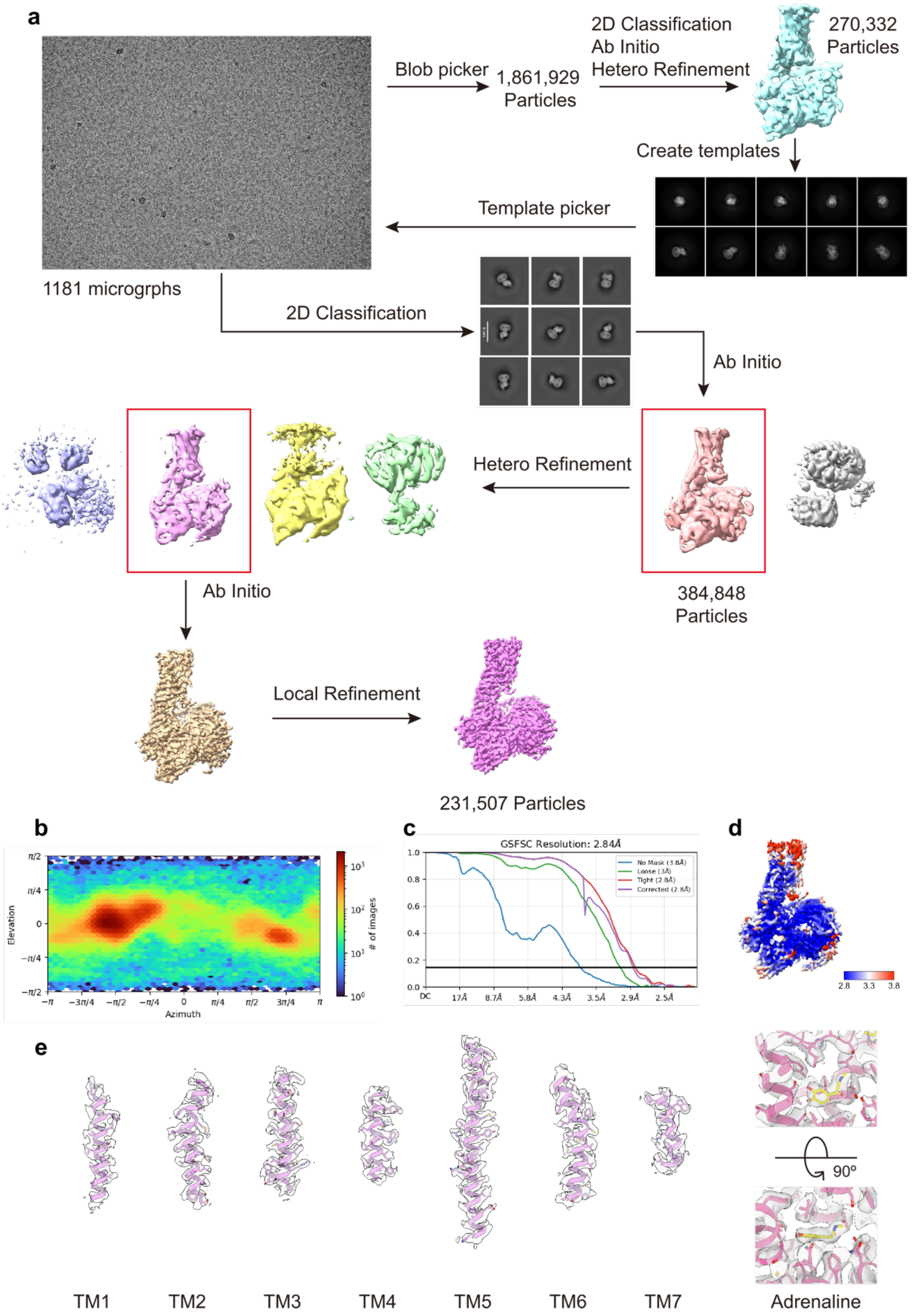

Supplementary Fig. 10. The cryo-EM data processing of the active D_1R-M74 mutant in complex with adrenaline. The cryo-EM data processing workflow: **a** Viewing direction distribution, **b** FSC plot, **c** Local resolution, and **d** for the D_1R-M74-miniGsiN-Gβ1γ2-Nb35-adrenaline complex. **e** Cryo-EM density map of individual D_1R-M74 receptor helices and adrenaline.

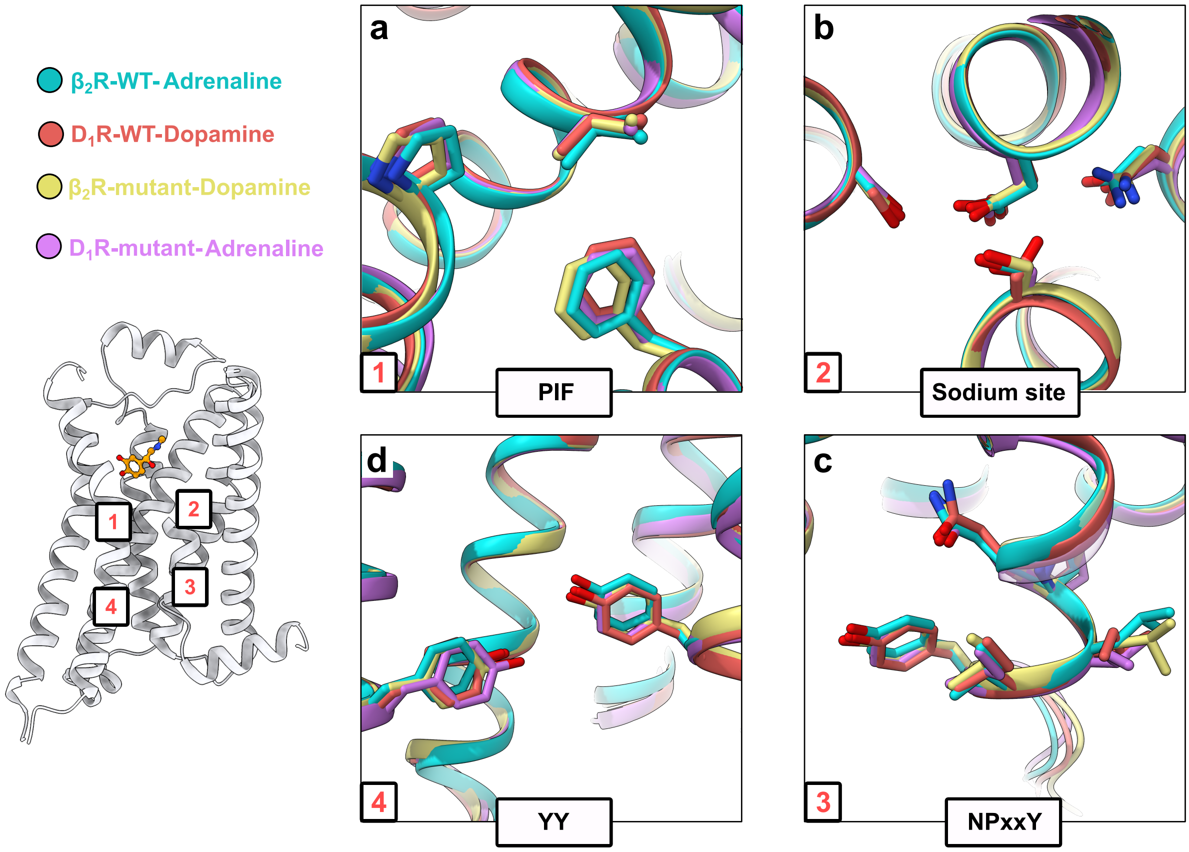

Supplementary Fig. 11. Activation microswitches in wild-type and mutant receptors. Comparison of key activation motifs in the D_1R-M74 (violet) and $β_2$R-M16 (yellow) cryo-EM structures to those in the $β_2$R-WT crystal structure (cyan, PDB accession code: 4LDO) and D_1R (light red, PDB accession code: 7LJD): **a** PIF motif, **b** Sodium binding site, **c** NPxxY motif, and **d** YY motif. Side chains are shown as sticks and each panel is labelled with a number indicating where the motif is located in the receptors, which is shown as a white cartoon.

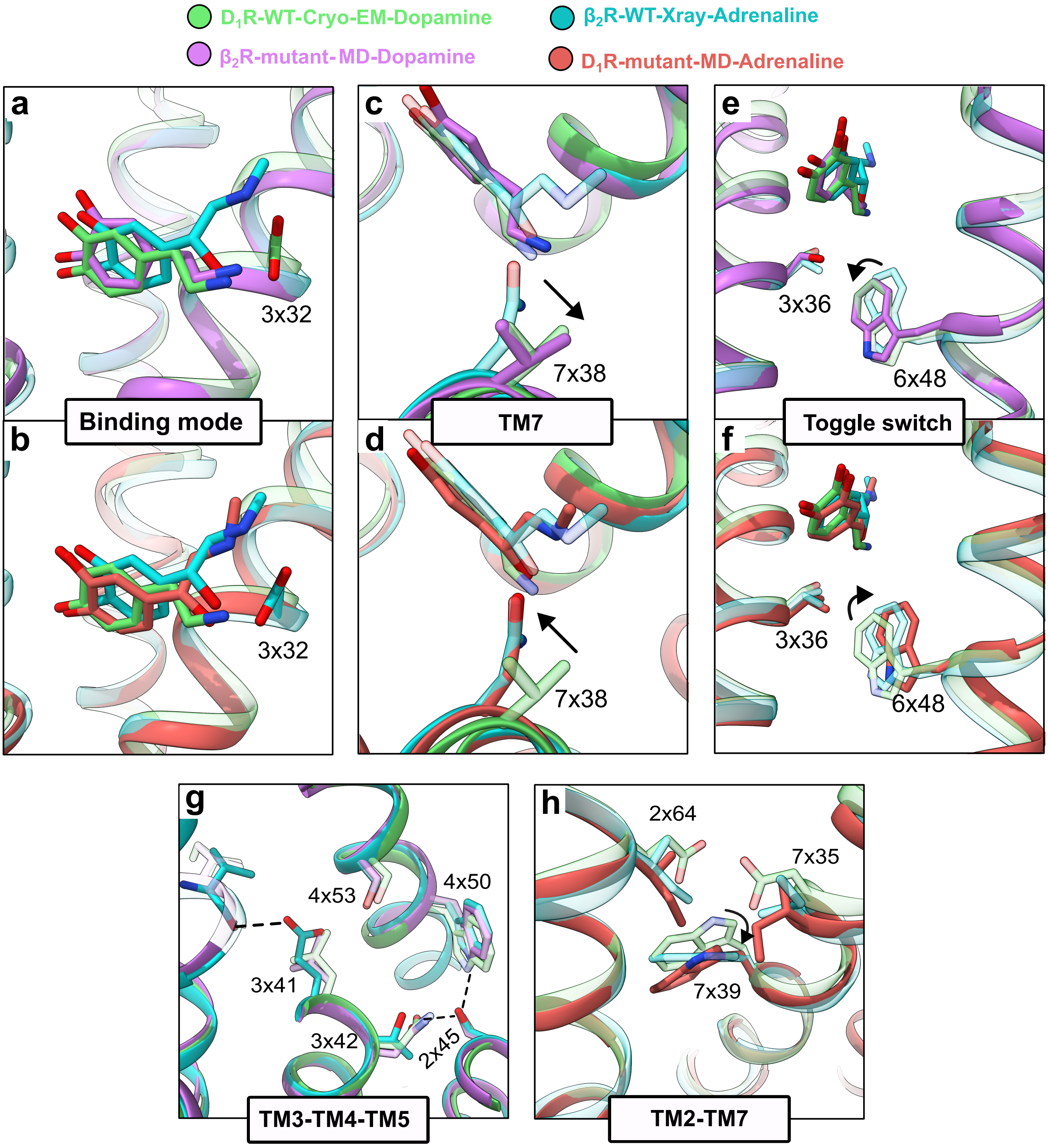

Supplementary Fig. 12. Comparison of MD simulations snapshots with experimental structures. In **a-h**, representative MD simulation snapshots of β_2R-M16 (violet) and D_1R-M74 (light red) are compared to experimentally determined structures of D_1R-WT (green, PDB accession code: 7LJD) and β_2R-WT (cyan, PDB accession code: 4LDO). The simulations of the mutants were prepared based on the WT structures. **a** Dopamine binding mode predicted by MD simulations of β_2R-M16. **b** Adrenaline binding mode predicted by MD simulations of D_1R-M74. **c-d** MD simulation predicted changes in position 7x38 in β_2R-M16 **c** and D_1R-M74 **d.** **e-f** MD simulation predicted changes in the toggle switch motif (W^{7x39}) in β_2R-M16 **e** and D_1R-M74 **f.** **g** MD simulation predicted changes in the TM3-TM4-TM5 interface in β_2R-M16. **h** MD simulation predicted changes in the TM2-TM7 interface in D_1R-M74. Ligands are depicted in stick representations. Side chains are shown as sticks and labelled with generic numbering. Distances and angles are labelled with black arrows. Hydrogen bonds are indicated by dashed black lines.

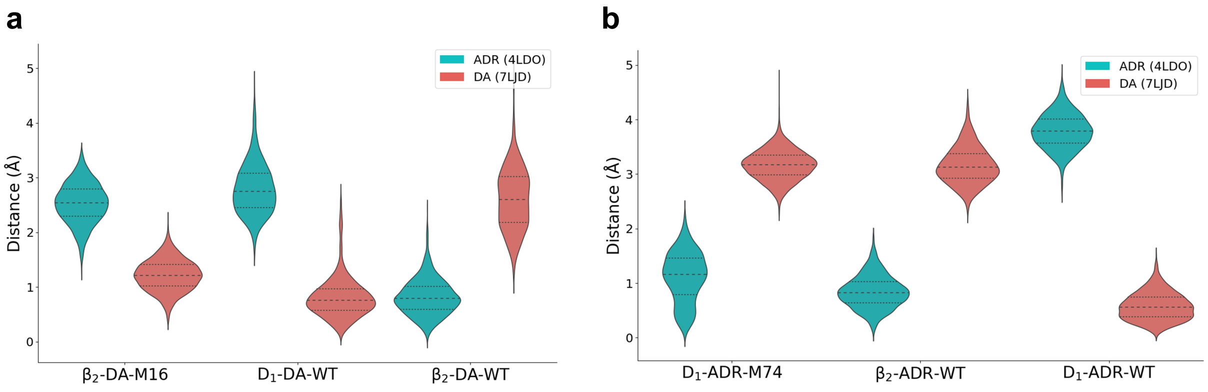

Supplementary Fig. 13. Analysis of the ligand binding mode in MD simulations of wild-type and mutant receptors. **a** Violin plots of the distance between the amine nitrogen of dopamine (DA) in MD simulations and its position in experimental structures of D_1R-WT (bound to dopamine, PDB accession code: 7LJD) and β_2R-WT (bound to adrenaline, PDB accession code: 4LDO). For β_2R-M16 and D_1R-WT, the amine nitrogen was positioned closest to that observed in the D_1R-WT experimental structure. In contrast, in the β_2R-WT simulation, the amine nitrogen was closer to the position observed in the β_2R-WT experimental structure. **b** Violin plots of the distance between the amine nitrogen of adrenaline (ADR) in MD simulations and its position in experimental structures of D_1R-WT (bound to dopamine, PDB accession code: 7LJD) and β_2R-WT (bound to adrenaline, PDB accession code: 4LDO). For D_1R-M74 and β_2R-WT, the amine nitrogen was positioned closest to that observed in the β_2R-WT experimental structure. In contrast, in the D_1R-WT simulation, the amine nitrogen was closer to the position observed in the D_1R-WT experimental structure. Data are based on three independent MD simulations of each receptor-ligand complex. Source data are provided as a Source Data file.

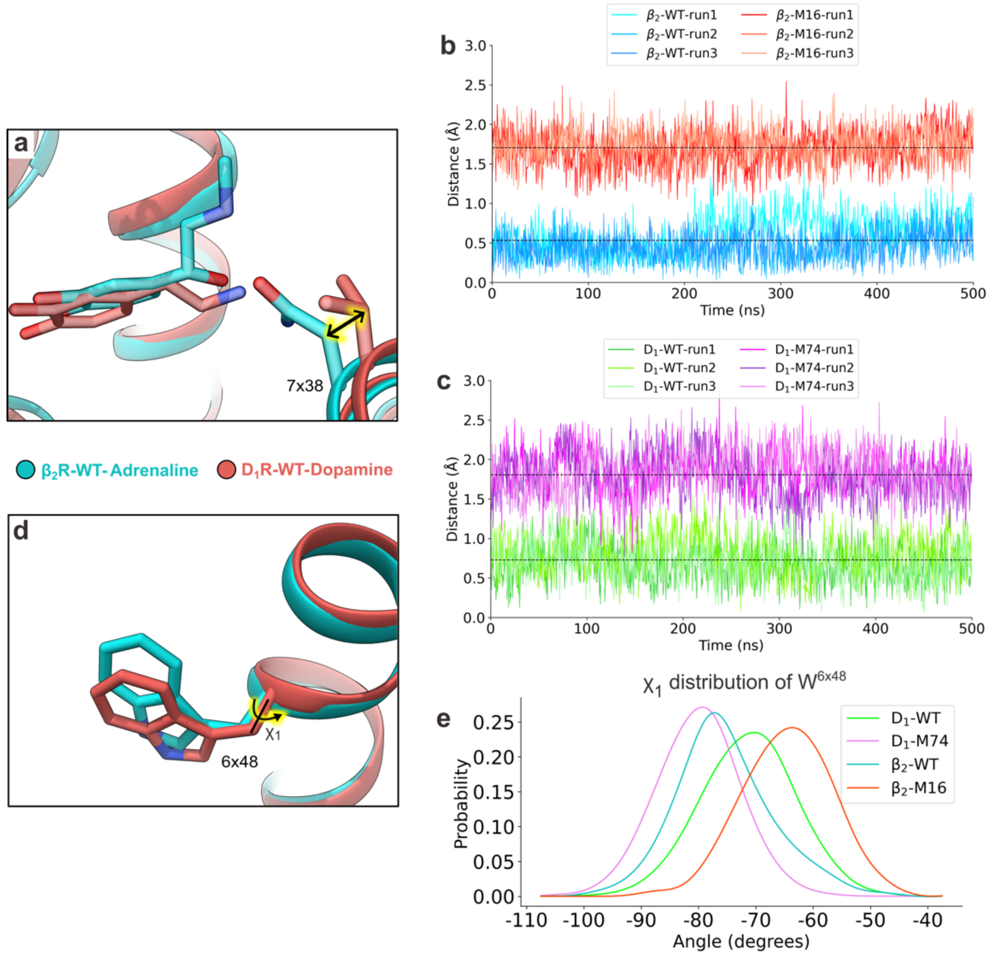

Supplementary Fig. 14. Analysis of residues 7x38 and 6x48 in MD simulations of wild-type and mutant receptors. **a** Differences in the orientation of the residue 7x38 side chain in the experimentally determined structures of β_2R-WT and D_1R-WT. The distance (1.2 Å) between the Cβ atoms at position 7×38 is indicated by a line, illustrating a difference in the binding site conformation. Ligands and key side chains are shown as sticks, and residues are labeled with generic numbering. In **b-c**, structures from the MD simulations were aligned to the experimental structures of the β_2R-WT **b** or D_1R-WT **c**, and distances were calculated between the Cβ atom of residue 7x38 in the snapshots and in the aligned experimental structure. Black dashed lines represent the average distance from three independent simulations. **b** Distance between residue 7×38 in MD snapshots of β_2R-WT or β_2R-M16 and in the experimentally determined structure of β_2R-WT (PDB accession code: 4LDO). In the β_2R-WT simulations, the side chain remained close to the position observed in the experimental structure. In the β_2R-M16 simulations, the side chain was displaced by 1.2 Å, which is similar to the difference observed between the experimental β_2R-WT and D_1R-WT structures shown in **a**. **c** Distance between residue 7×38 in MD snapshots of D_1R-WT or D_1R-M74 and in the experimentally determined structure of D_1R-WT (PDB accession code: 7LJD). In the D_1R-WT simulations, the side chain remained close to the position observed in the D_1R-WT experimental structure. In the D_1R-M74 simulations, the side chain was displaced by 1.1 Å, which is similar to the difference observed between the experimental β_2R-WT and D_1R-WT structures shown in **a**. **d** Differences in the residue W^{6x48} side chain torsion in the experimental structures of β_2R-WT and D_1R-WT**.** The χ_1 torsion angle is indicated by a black arrow. Ligands and key side chains are shown as sticks and residues are labeled with generic numbering. **e** Distributions of the W^{6x48} χ_1 torsion angle in MD simulations of WT and mutant receptors. Curves represent torsion angles calculated from three independent MD simulations (green, D_1R-WT; violet, D_1R-M74; cyan, β_2R-WT; red, β_2R-M16). The 6x48 χ_1 torsion angle distribution of β_2R-M16 resembles that of D_1R-WT and, conversely, the distribution obtained for D_1R-M74 more closely overlaps with that from the β_2R-WT simulations. Source data are provided as a Source Data file.

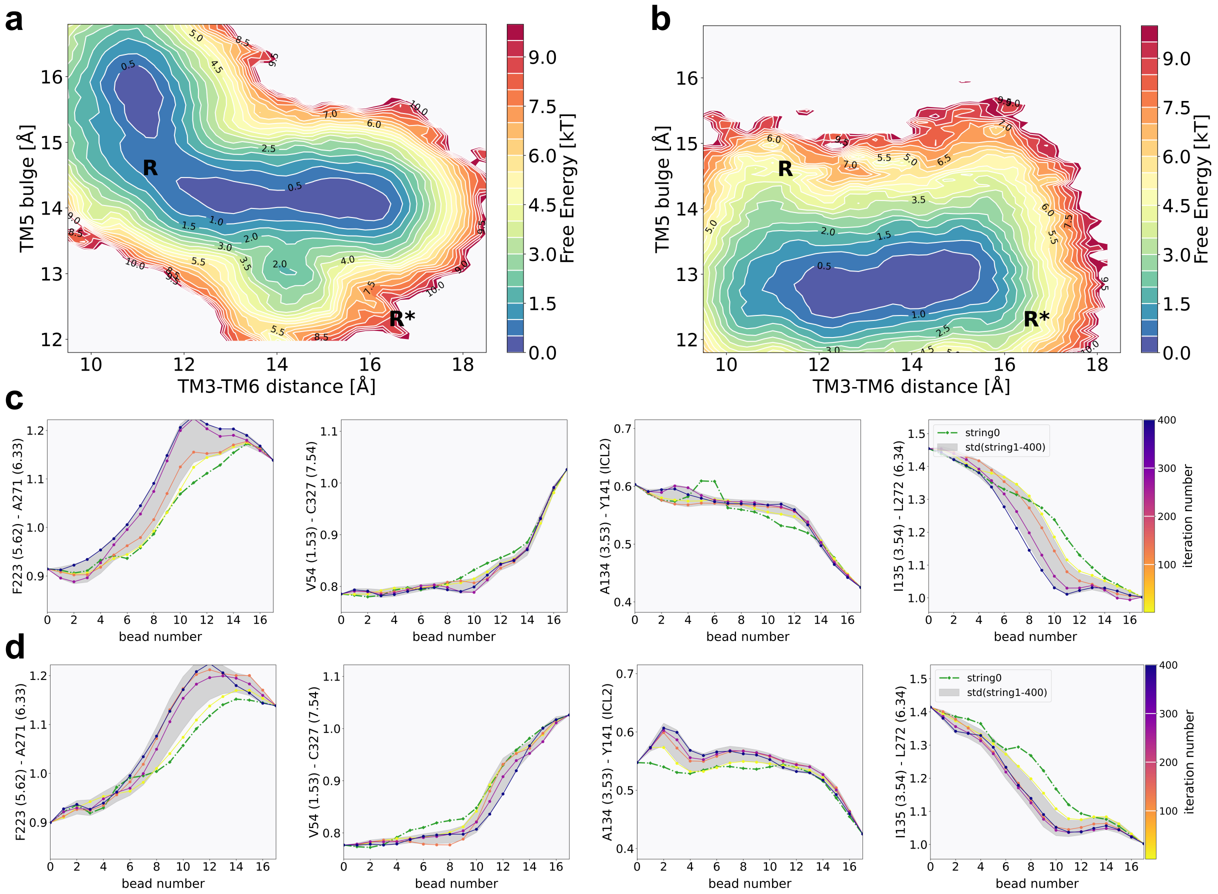

Supplementary Fig. 15. Enhanced sampling MD simulations. Free energy surface of **a** β_2R-WT and **b** β_2R-M16 projected along TM3-TM6 distance (Cα distance between R131$^{3.50}$ and E267$^{6.30}$) and TM5 bulge (Cα distance between S207$^{5.46}$ and G315$^{7.42}$) for the apo state. Strings averaged over hundreds of iterations for **c** β_2R-WT and **d** β_2R-M16 initiated from the active structure (PDB accession code: 4LDO). Four important collective variables (CVs) are shown on the y-axis to evaluate the string convergence. The x-axis shows the evolution of the string points towards the inactive state. The position of a representative active and inactive receptor structure is represented by R* and R, respectively. Source data are provided as a Source Data file.

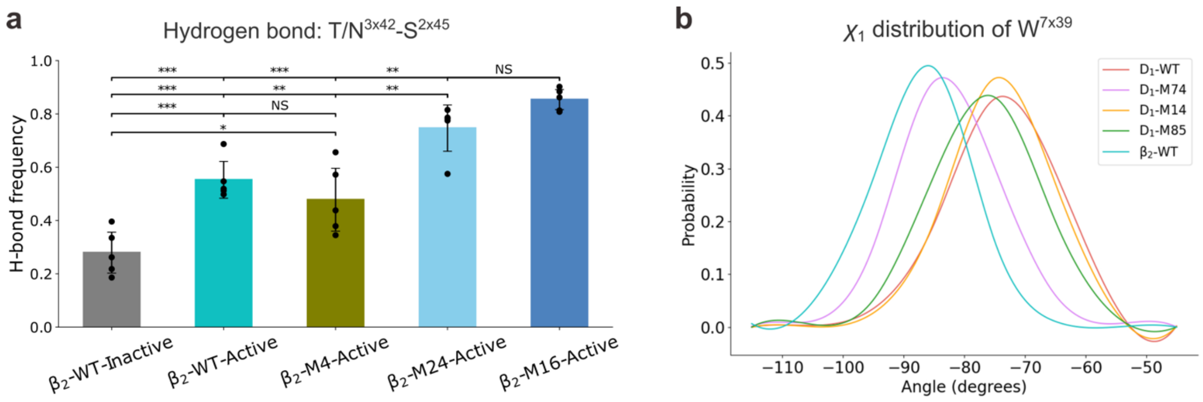

Supplementary Fig. 16. Analysis of hydrogen bonding networks and the side chain rotamer of position 7x39 in MD simulations of wild-type and mutant receptors. **a** The frequency of hydrogen bonding (H-bond) between S74^{2x45} and N/T123^{3x42} in MD simulations of β$_2$R-WT and mutants. Data bars represent the mean ± SD from five independent simulations (grey, β$_2$R-WT-inactive; cyan, β$_2$R-WT-active; olive, β$_2$R-M4-active; light blue, β$_2$R-M24-active; blue, β$_2$R-M16-active). Statistical significance was assessed with a pairwise two-tailed Welch's t-tests. Significance levels: not significant (NS), $p > 0.05$, *p < 0.05, **p < 0.01, ***p < 0.001). An active-like hydrogen bonding network is stabilized in simulations of β$_2$R-M24-active and β$_2$R-M16-active. **b** The distribution curves of W318/313^{7x39} χ_1 dihedral angle in MD simulations of β$_2$R-WT, D$_1$R-WT and mutants. Data curves represent dihedral angles collected from five independent simulations (light red, D$_1$R-WT-active; orange, D$_1$R-M14-active; violet, D$_1$R-M74-active; green, D$_1$R-M85-active; cyan, β$_2$R-WT-active). The results for the D$_1$R with mutations both within and outside the binding site (D$_1$R-M74) most closely resembles that obtained for the β$_2$R-WT. Data curves represent dihedral angles from three independent simulations. Subsets of the data in panels a and b are shown in Fig. 6c and e, respectively. Source data are provided as a Source Data file.

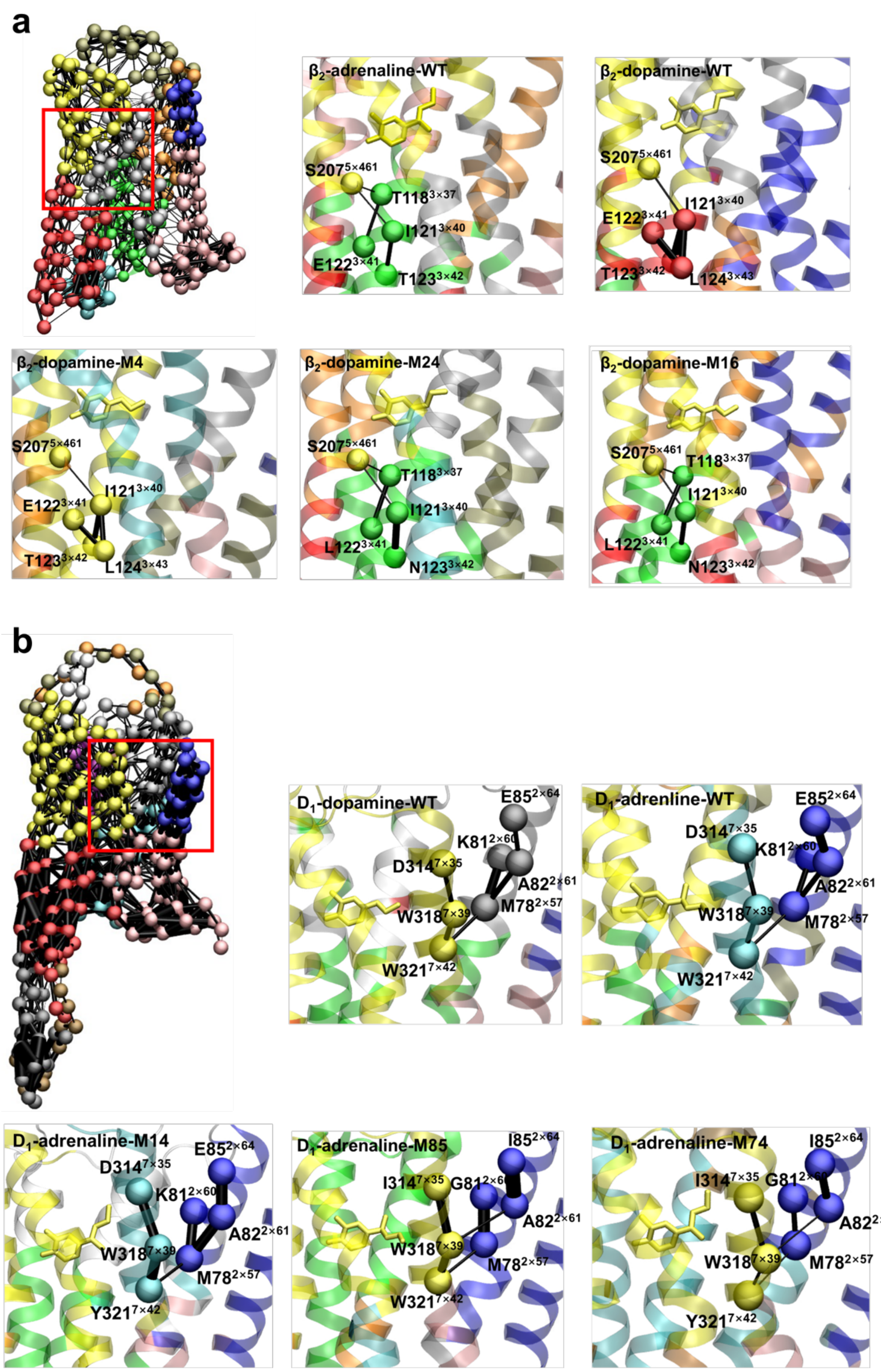

Supplementary Fig. 17. Network analysis using β_2R and D_1R simulations. a Overall residue interaction network of one representative β_2R simulation and local interaction networks connecting residues within and outside the binding site for WT and three mutant receptors (M4, M24, and M16). **b** Overall residue interaction network of one representative D_1R simulation and local interaction networks connecting residues within and outside the binding site for WT and three mutant receptors (M14, M85, and M74). In **a** and **b**, nodes (residues) are shown as spheres and connecting edges are depicted as lines, with line width scaled by betweenness centrality.

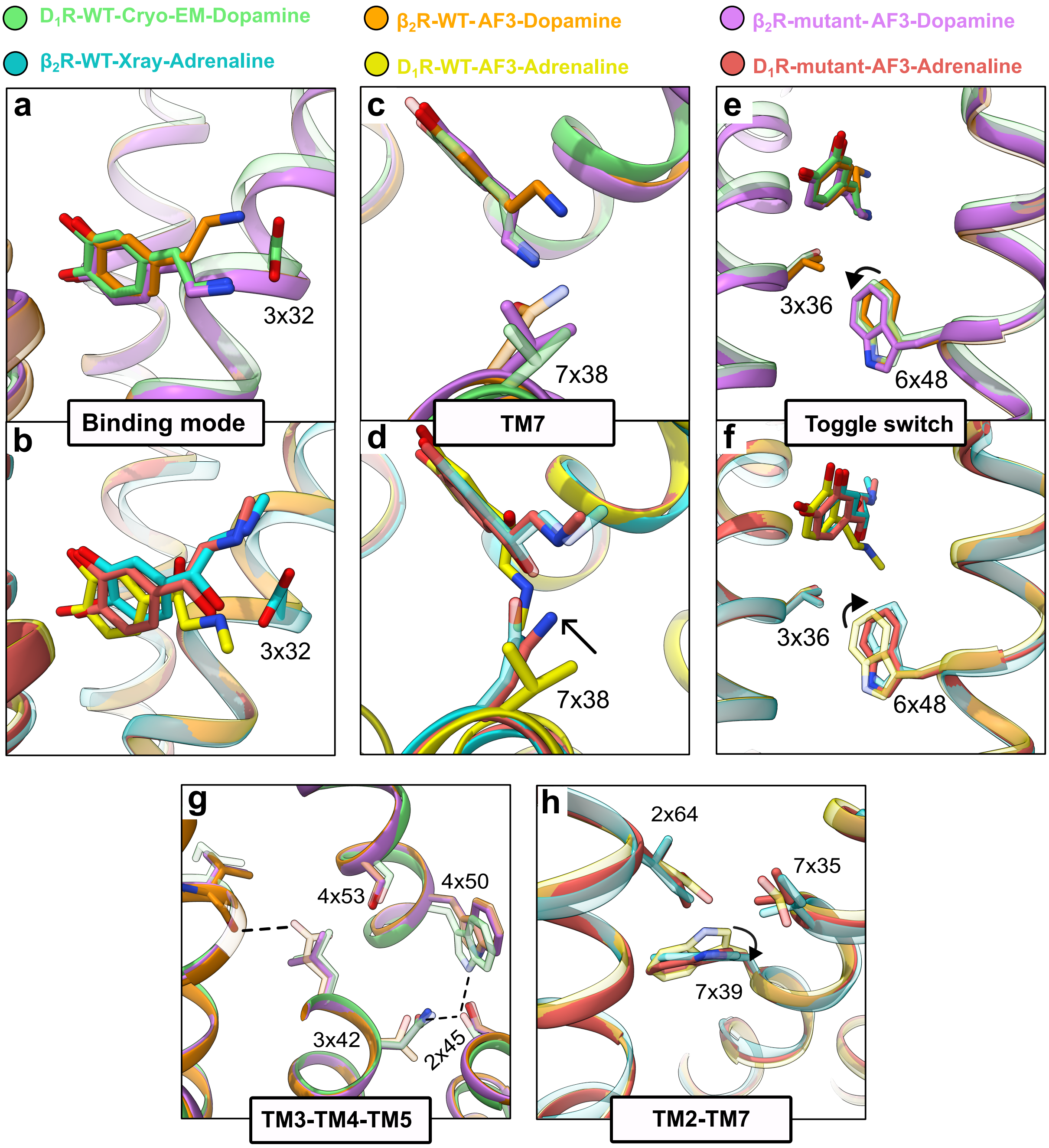

Supplementary Fig. 18. Comparison of AlphaFold3 (AF3) models with experimental structures. **a** Comparisons of dopamine binding modes predicted by AF3 in β_2R-WT (orange) and β_2R-M16 (violet) with that in a cryo-EM structure of the D_1R-WT (green, PDB accession code: 7LJD). The AF3 models predict that the mutations lead to a switch of the dopamine binding mode and the predicted complex resembles the experimentally determined D_1R-WT in complex with dopamine. **b** Comparisons of adrenaline binding modes predicted by AF3 in D_1R-WT (yellow) and D_1R-M74 (light red) to that in a crystal structure of β_2R-WT (cyan, PDB accession code: 4LDO). The AF3 models predict that the mutations lead to a switch of the adrenaline binding mode and the predicted complex resembles the experimentally determined β_2R-WT in complex with adrenaline. **c-d** AlphaFold3 predicted changes in position 7x38 in **c** β_2R-M16 and **d** D_1R-M74 compared to the experimental structures of D_1R-WT and β_2R-WT, respectively. **e-f** AlphaFold3 predicted changes affecting the toggle switch motif (W^{7x39}) in β_2R-M16 **e** and D_1R-M74 **f** to the experimental structures of β_2R-WT and D_1R-WT, respectively. **g** AlphaFold3 predicted changes affecting the TM3-TM4-TM5 interface in β_2R-M16 compared to the D_1R-WT experimental structure. **h** AlphaFold3 predicted changes affecting the TM2-TM7 interface in D_1R-M74 compared to the β_2R-WT experimental structure. Ligands are depicted in stick representations. Side chains are shown as sticks and labelled with generic numbering. Distances and angles are labelled with black arrows. Hydrogen bonds are indicated by dashed black lines.

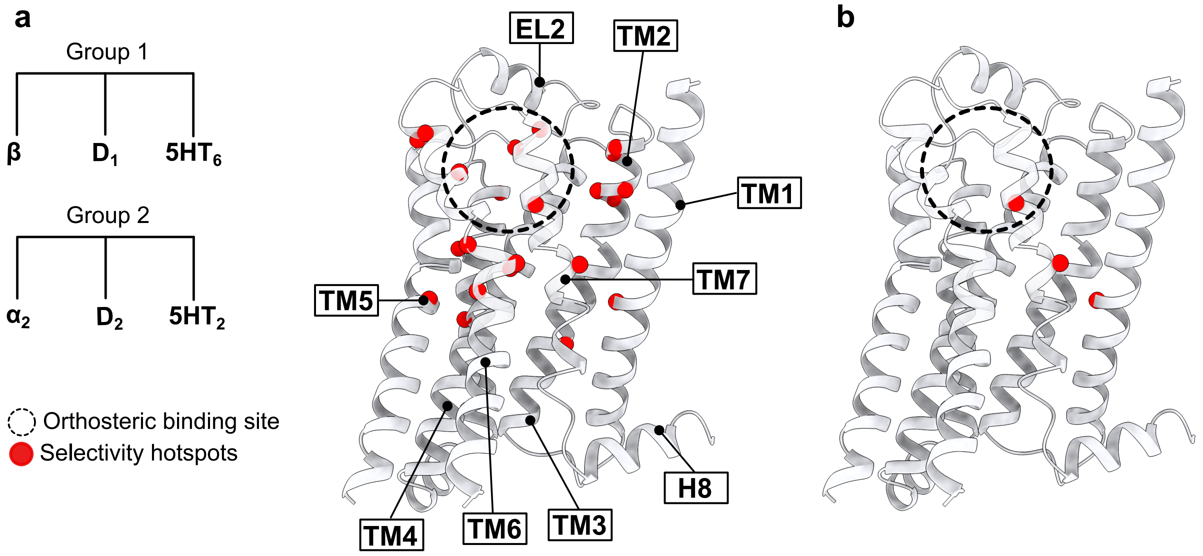

Supplementary Fig. 19. Selectivity hotspots in adrenergic, dopaminergic, and serotonergic GPCRs. a Analysis of two groups of aminergic receptors to identify selectivity hotspots. **b** Selectivity hotspots in the D_2-like dopaminergic and α_2-adrenergic receptors. Receptors are shown as white cartoon representations, with red spheres highlighting the identified selectivity hotspots. The orthosteric binding site is indicated by black dashed lines.

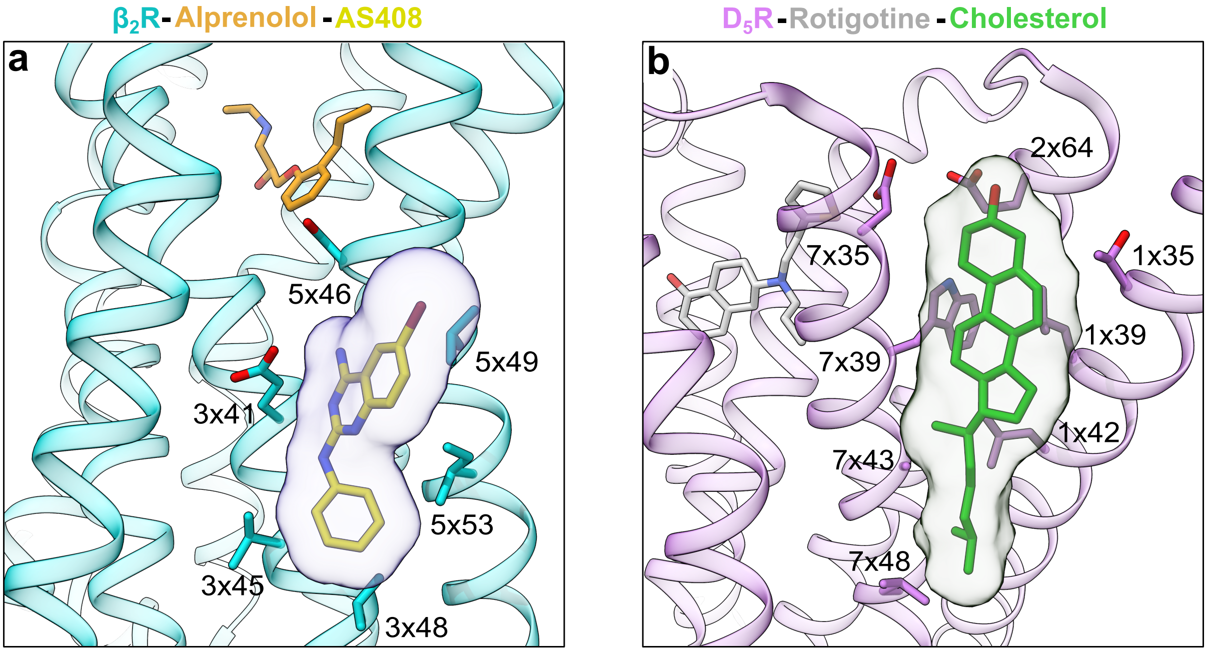

Supplementary Fig. 20. Allosteric sites in experimental β$_2$R and D$_1$R structures. **a** Binding mode of allosteric ligand AS408 (yellow sticks and white surface) and orthosteric ligand alprenolol (orange sticks) to the β$_2$R (cyan cartoon, PDB accession code: 6OBA). **b** Binding mode of cholesterol (green sticks and white surface) and orthosteric ligand rotigotine (gray sticks) bound to the D$_5$R (pink cartoon, PDB accession code: 8RIV). Side chains are shown as sticks and labeled with generic residue numbers.

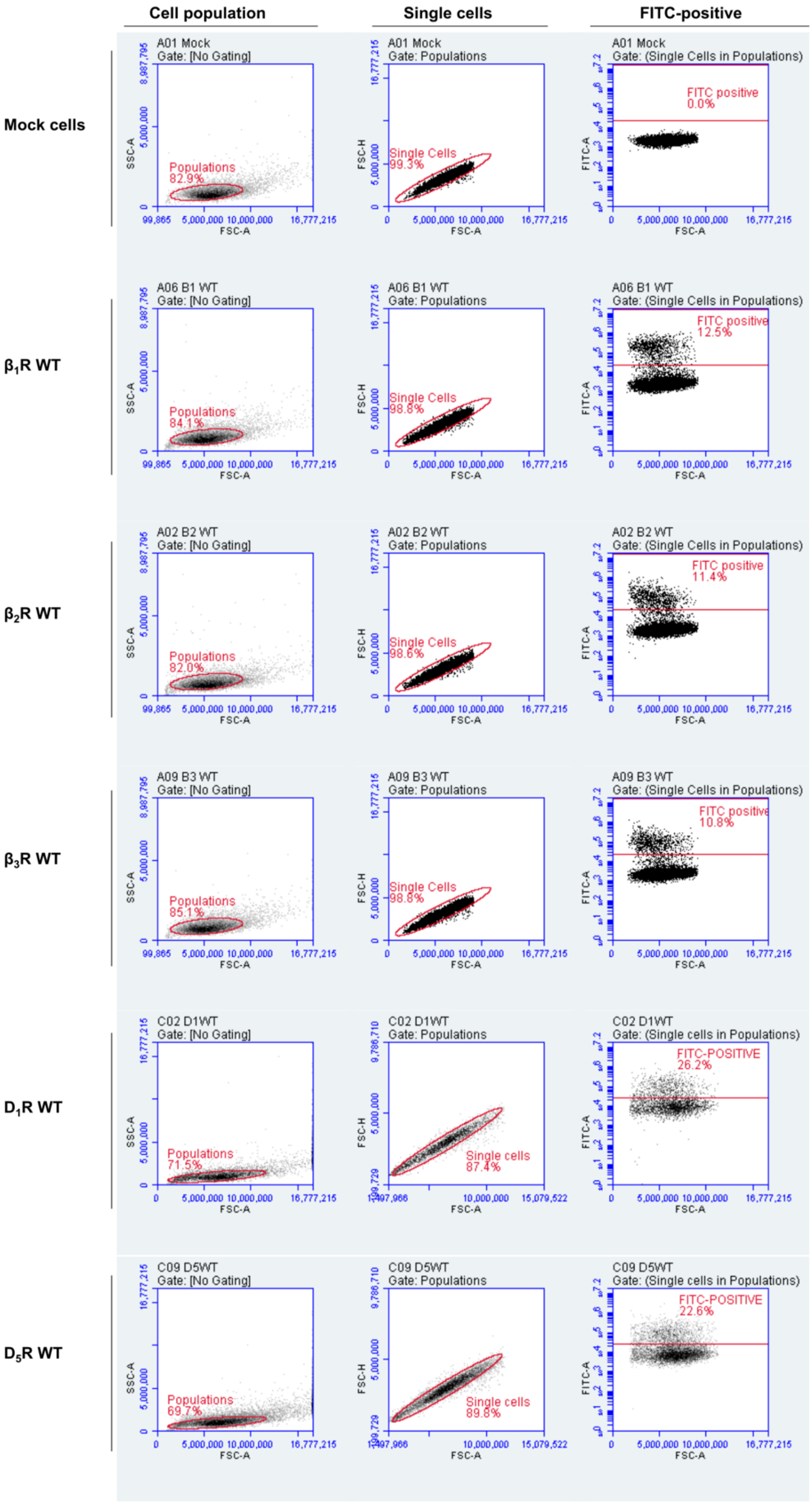

Supplementary Fig. 21. Flow cytometry gating strategy. Representative gating strategy using mock-treated and WT cells. The main cell population was gated on SSC-A vs FSC-A, followed by singlet selection on FSC-H vs FSC-A within the cell population gate. FITC-positive cells were subsequently identified on FITC-A vs FSC-A within the singlet gate. Mock-treated cells were used to define background fluorescence and set the FITC-positive threshold.

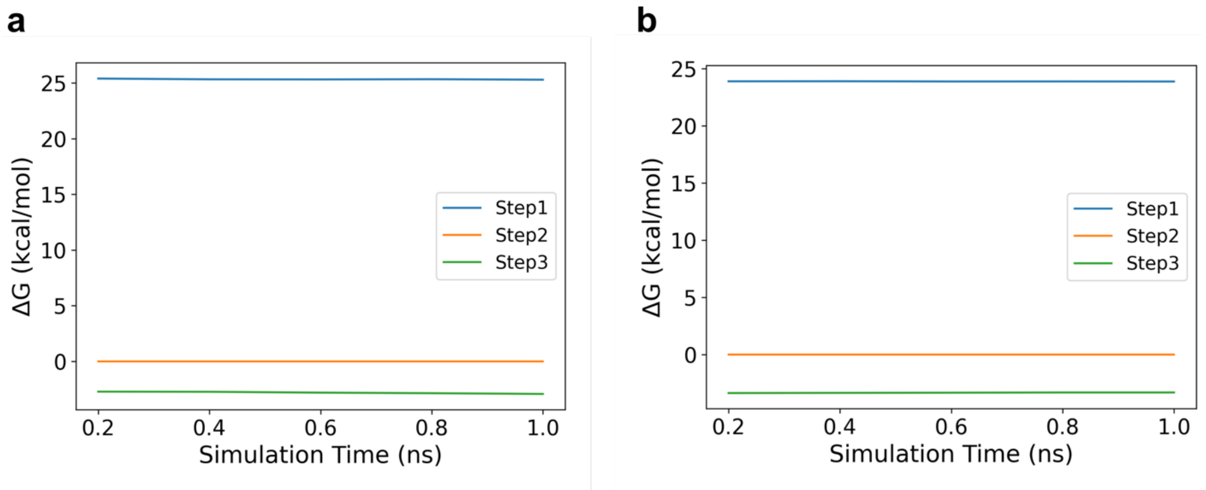

Supplementary Fig. 22. Convergence of free energy estimates from FEP calculations. Time-course of BAR free energy estimates (ΔG) for the used FEP protocol (Step1-Step3) for a representative alchemical transformation of noradrenaline to dopamine. Data correspond to the production phase of the simulations for the ligand-receptor complex (**a**) and the ligand in aqueous solution (**b**). ΔG values remain stable over the production time window, indicating that the simulations are equilibrated and that the free energy estimates are converged. Source data are provided as a Source Data file.

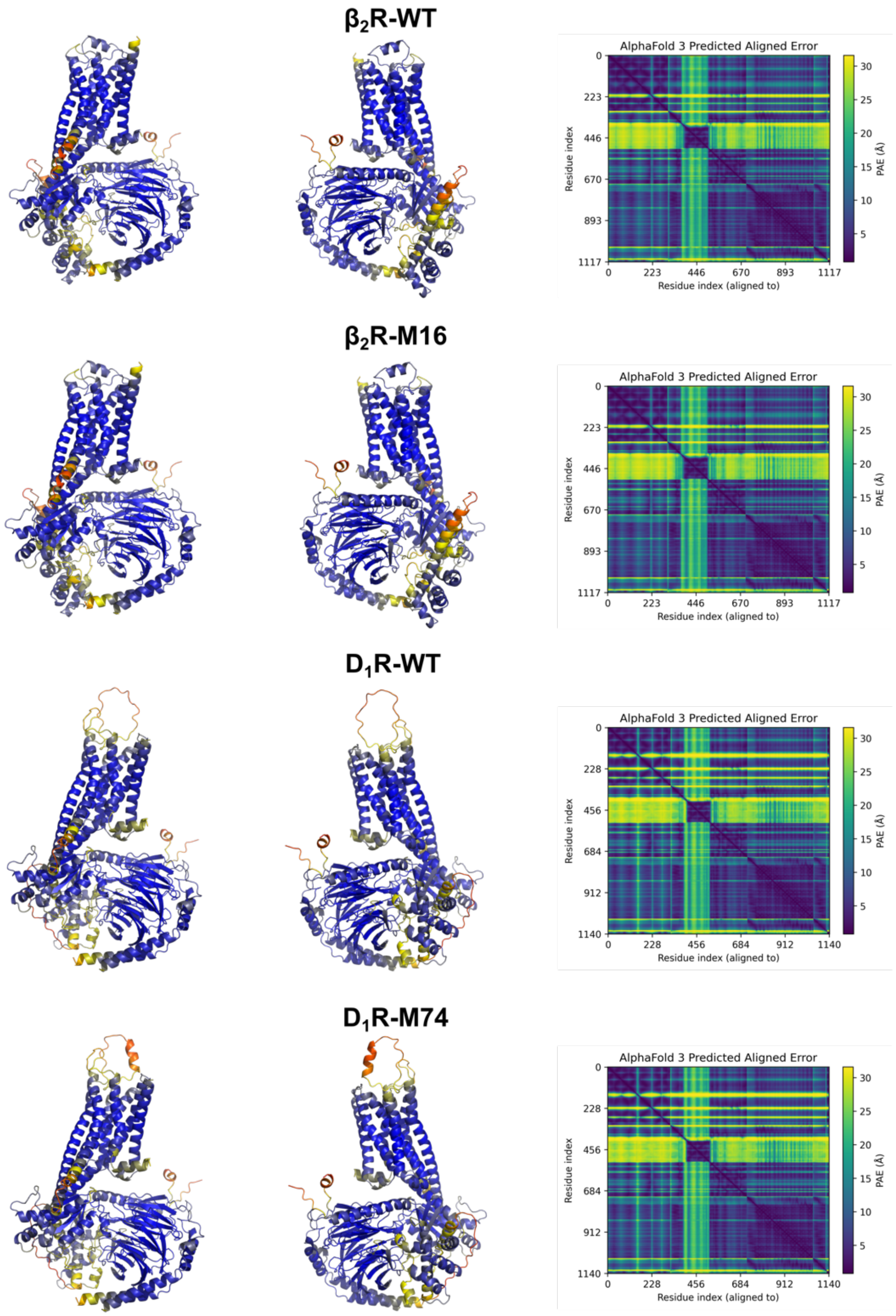

Supplementary Fig. 23. Confidence metrics of AlphaFold3 predicted complexes. For each complex, the left and center panels show two views of the predicted structure colored by pLDDT score (blue, high confidence; yellow, intermediate confidence; orange/red, low confidence). The right panel shows the corresponding PAE score, indicating the expected positional error between residue pairs across the protein-ligand complex.

Supplementary Table 1. Identified selectivity hotspots and their conservation, BLOSUM62 scores, and location.

Generic residue numbering	Hotspot residue (Conservation, %)		BLOSUM62 score	Location in β_2R and D_1R structures				Hotspot region
	β_2R	D_1R		Orthosteric site	interhelical	Surface exposed	Close to G protein binding site	
1x54	I55(98)	C45(100)	-1				Yes	-
2x37	T66(100)	K57(97)	-1			Yes	Yes	-
2x60	G90(96)	K81(100)	-2		Yes			TM2-TM7
3x36	V117(100)	S107(100)	-2	Yes				Binding site
3x41	E122(100)	L112(100)	-3		Yes			TM3-TM4-TM5
45x52	F193(100)	S188(92)	-2	Yes				Binding site
5x56	F217(100)	V212(98)	-1			Yes		-
6x28	L266(83)	K265(82)	-2			Yes	Yes	-
6x46	L284(100)	C283(100)	-1			Yes		-
7x38	N312(100)	V317(91)	-3	Yes				Binding site
7x55	R328(100)	F333(99)	-3			Yes	Yes	-

Supplementary Table 2. Mutated positions and rationale for their selection. All positions are >80% conserved in either the β adrenergic or D_1-like dopaminergic subfamily.

Position	Receptor	Hotspot residue[a]	Hotspot region[b]	Binding site region		Frequency[e]
				1st shell[c]	2nd shell[d]	
**1x46**	Both	-	-	-	Yes	4
**1x54**	D_1R	Yes	-	-	-	7
**2x49**	β_2R	-	-	-	Yes	3
**2x53**	Both	-	-	-	Yes	23
**2x57**	Both	-	-	-	Yes	6
**2x60**	Both	Yes*	Yes	-	Yes	29
**2x64**	Both	-	Yes	-	-	23
**3x29**	Both	-	-	-	Yes	2
**3x36**	Both	Yes*	Yes	Yes	-	65
**3x41**	Both	Yes*	Yes	-	-	31
**3x42**	Both	-	Yes	-	-	28
**45x52**	Both	Yes*	Yes	Yes	-	33
**45x52+2**	Both	-	Yes	Yes	-	9
**EL2**	D_1R (H164)	-	-	-	Yes	2
**4x48[f]**	D_1R	-	-	-	-	1
**4x49**	Both	-	Yes	-	-	39
**4x52**	D_1R	-	Yes	-	-	10
**4x56**	Both	-	-	-	Yes	7
**5x46**	D_1R	-	-	-	Yes	1
**6x46**	D_1R	Yes	-	-	-	1
**6x54**	Both	-	-	-	Yes	8
**6x56**	Both	-	-	-	Yes	7
**6x58**	D_1R	-	-	-	Yes	2
**6x59**	D_1R	-	-	-	Yes	1
**7x31[f]**	Both	-	-	-	-	3
**7x34**	Both	-	Yes	-	Yes	10
**7x35**	Both	-	Yes	-	Yes	24
**7x38**	Both	Yes*	Yes	Yes	-	109
**7x42**	Both	-	Yes	Yes	-	36

[a] Hotspot residue: Identified through sequence analysis. The five prioritized hotspot residues, which are part of the binding site (positions 3x36, 45x52, and 7x38), TM3-TM4-TM5 (3x41) or TM2-TM7 (2x60) interfaces, are marked with an asterisk.
[b] Hotspot region: A residue within 5 Å of a prioritized hotspot residue, including the hotspot residues. The eight positions were selected based on: Interactions with the hotspot residue side chain (4x52 and 4x49), participation in interhelical interactions (2x64, 3x42, 4x49, and 7x35), interactions with the orthosteric ligand (45x52+2 and 7x42), or interactions with binding site residues (7x34).
[c] Binding site residue, first shell: A residue within 4 Å of the ligand.
[d] Binding site residue, second shell: A residue within 4 Å of a binding site residue, excluding binding site residues in the first shell.
[e] Number of evaluated receptor mutants (D_1R and β_2R) that include the residue position.
[f] Does not belong to the categories in a–d. Position 4x48 was selected because this residue is close to the hotspot residue 3x41. Position 7x31 was selected because this residue is part of a polar interaction network at the opening of the orthosteric site and interacts with EL2.

Supplementary Table 3. Cryo-EM data collection, refinement, and validation statistics.

	**β_2R-M16 (V117S/E122L/T123N/N312V)-dopamine-miniG$_{siN}$-G$\beta_1\gamma_2$-Nb35-scFv16 complex (EMDB-63431) (PDB: 9LW5)**	**D_1R-M74 (K81G/E85I/S107V/A147V V317N/D314I/W321Y)-adrenaline-miniG$_{siN}$-G$\beta_1\gamma_2$-Nb35 complex (EMDB-63440) (PDB: 9LWC)**
**Data collection and processing**		
Magnification	81000	81000
Voltage (kV)	300	300
Electron exposure (e–/Å^2)	50	50
Defocus range (μm)	1.10-1.80	1.10-1.80
Pixel size (Å)	1.0825	1.0825
Symmetry imposed	C1	C1
Initial particle images (no.)	1,420,939	1,861,929
Final particle images (no.)	230,232	231,507
Map resolution (Å)	2.86	2.84
FSC threshold	0.143	0.143
**Refinement**		
Initial model used (PDB code)	3SN6, 7KH0	7F0T
Model resolution (Å)	2.86	2.84
FSC threshold	0.143	0.143
Map sharpening *B* factor (Å^2)	-112.6	-84.8
Model composition		
Non-hydrogen atoms	9418	6238
Protein residues	1249	863
Ligands	1	1
B factors (Å^2)		
Protein	62.95	42.03
Ligand	69.79	20.00
R.m.s. deviations		
Bond lengths (Å)	0.003(0)	0.004(0)
Bond angles (°)	0.511(0)	0.671(0)
Validation		
MolProbity score	1.38	2.32
Clashscore	5.33	8.05
Poor rotamers (%)	0.00	5.71
Ramachandran plot		
Favored (%)	97.55	95.62
Allowed (%)	2.37	4.38
Disallowed (%)	0.08	0.00

Supplementary Table 4. Setup of MD and FEP calculations.

System	Box dimensions/ Sphere radius	Total number of atoms	Total number of water molecules	Total number of lipid molecules (POPC)	Salt concentration
Brute force MD					
β_2R-WT-inactive	7.40924 x 7.40924 x 9.54376 nm	54325	10302	140	0.15 M NaCl
β_2R-adrenaline-M16	7.41913 x 7.41913 x 9.73517 nm	55802	10802	140	0.15 M NaCl
β_2R-adrenaline-WT	7.47434 x 7.47434 x 9.53061 nm	55325	10643	140	0.15 M NaCl
β_2R-dopamine-M16	7.42422 x 7.42422 x 9.73249 nm	55735	10781	140	0.15 M NaCl
β_2R-dopamine-M24	7.41075 x 7.41075 x 9.85755 nm	56107	10904	140	0.15 M NaCl
β_2R-dopamine-M4	7.47509 x 7.47509 x 9.60041 nm	55480	10697	140	0.15 M NaCl
β_2R-dopamine-WT	7.44392 x 7.44392 x 9.65198 nm	55606	10738	140	0.15 M NaCl
D_1R-adrenaline-M14	7.65076 x 7.65076 x 12.77566 nm	76851	17142	150	0.15 M NaCl
D_1R-adrenaline-M74	7.65674 x 7.65674 x 12.74276 nm	76874	17148	150	0.15 M NaCl
D_1R-adrenaline-M85	7.43749 x 7.43749 x 12.62284 nm	72099	16005	140	0.15 M NaCl
D_1R-adrenaline-WT	7.69664 x 7.69664 x 12.59313 nm	76847	17140	150	0.15 M NaCl
D_1R-dopamine-WT	7.70648 x 7.70648 x 12.57116 nm	76834	17137	150	0.15 M NaCl

Enhanced sampling MD					
β_2R-apo-M16 (Enhanced sampling)	7.32065 x 7.32065 x 9.87758 nm	55783	10805	140	0.15 M NaCl
β_2R-apo-WT (Enhanced sampling)	8.14193 x 8.14193 x 9.16617 nm	55252	10628	140	0.15 M NaCl
MD/FEP					
β_2R-noradrenaline-WT	25 Å	9739	316	79	N/A
β_2R-noradrenaline-M1	25 Å	9718	316	79	N/A
β_2R-noradrenaline-M3	25 Å	9741	316	79	N/A
β_2R-noradrenaline-M4	25 Å	9736	316	79	N/A
D_1R-noradrenaline-WT	25 Å	13852	283	149	N/A
D_1R-noradrenaline-M1	25 Å	13857	283	149	N/A
D_1R-noradrenaline-M3	25 Å	13847	282	149	N/A
D_1R-noradrenaline-M4	25 Å	13852	282	149	N/A
