# Supplementary material for: Molecular mechanisms of native ligand selectivity in catecholamine G protein-coupled receptors
Source: Nat Commun. 2026 Apr 23;17:4112. doi: 10.1038/s41467-026-71361-8 (PMC13150006; doi:10.1038/s41467-026-71361-8)
Supplement: Supplementary file 2 — Description of Additional Supplementary Files [file 41467_2026_71361_MOESM2_ESM.pdf]

File name: Supplementary Data 1
Description: Pharmacology and expression data.

File name: Supplementary Data 2
Description: Initial and final coordinate files for MD simulations and FEP calculations.
