# Supplementary material for: Molecular mechanisms of native ligand selectivity in catecholamine G protein-coupled receptors
Source: Nat Commun. 2026 Apr 23;17:4112. doi: 10.1038/s41467-026-71361-8 (PMC13150006; doi:10.1038/s41467-026-71361-8)
Supplement: Supplementary file 5 — Reporting Summary [file 41467_2026_71361_MOESM5_ESM.pdf]

nature portfolio

Corresponding author(s): Xiangyu Liu, Dan Larhammar, Jens Carlsson

Last updated by author(s): 03/15/2026

Reporting Summary

Nature Portfolio wishes to improve the reproducibility of the work that we publish. This form provides structure for consistency and transparency in reporting. For further information on Nature Portfolio policies, see our Editorial Policies and the Editorial Policy Checklist.

Statistics

For all statistical analyses, confirm that the following items are present in the figure legend, table legend, main text, or Methods section.

n/a	Confirmed	
☐	☒	The exact sample size (*n*) for each experimental group/condition, given as a discrete number and unit of measurement
☐	☒	A statement on whether measurements were taken from distinct samples or whether the same sample was measured repeatedly
☐	☒	The statistical test(s) used AND whether they are one- or two-sided<br>*Only common tests should be described solely by name; describe more complex techniques in the Methods section.*
☒	☐	A description of all covariates tested
☒	☐	A description of any assumptions or corrections, such as tests of normality and adjustment for multiple comparisons
☐	☒	A full description of the statistical parameters including central tendency (e.g. means) or other basic estimates (e.g. regression coefficient) AND variation (e.g. standard deviation) or associated estimates of uncertainty (e.g. confidence intervals)
☐	☒	For null hypothesis testing, the test statistic (e.g. *F*, *t*, *r*) with confidence intervals, effect sizes, degrees of freedom and *P* value noted<br>*Give P values as exact values whenever suitable.*
☒	☐	For Bayesian analysis, information on the choice of priors and Markov chain Monte Carlo settings
☒	☐	For hierarchical and complex designs, identification of the appropriate level for tests and full reporting of outcomes
☒	☐	Estimates of effect sizes (e.g. Cohen's *d*, Pearson's *r*), indicating how they were calculated

Our web collection on statistics for biologists contains articles on many of the points above.

Software and code

Policy information about availability of computer code

Data collection	In vitro biological experiments:<br>• Cyclic adenosine monophosphate (cAMP) assay: Plates were read on a FluoStar Omega plate Reader (BMG Labtech) and the fluorescence emission was read with two wavelengths (665nm and 620nm).<br>• Bioluminescence resonance energy transfer (BRET) G protein activation assay: Plates were read on a Tecan Spark multimode microplate reader (Tecan, Männedorf, Switzerland) equipped with filters for BRET2 (center wavelength/bandwidth: 400/70 nm for donor and 515/20 nm for acceptor) at room temperature.<br>• Cell surface expression: Flow cytometry was preformed using Guava® easyCyte™ (Merck Millipore), or BD Accuri™ C6 (BD Biosciences).<br><br>Structural biology experiments:<br>• Cryo-EM: Titan Krios G3i microscope (300 kV) with K3 direct electron detector and GIF Quantum 1967 energy filter (Thermo Fisher Scientific); Gatan Microscopy Suite® (GMS)-3.5; Data collection: Batch acquisition via AutoEMation.<br><br>Sequence bioinformatics:<br>• RefSeq at NCBI, GPCRdb, AlphaFold2 database and AlphaFold3.<br><br>Molecular modeling:<br>• Protein Data Bank (PDB).
Data analysis	• In vitro biological experiments: Graphpad Prism v9, Guava® InCyte software, BD Accuri™ C6 Plus Software, and Microsoft Excel v16.56.<br>• Sequence bioinformatics: UMAP, Jalview, Biopython, AlphaFold3 v3.0.0.

• Structural biology experiments: AutoEMation, cryoSPARC v4.5.1, PHENIX v1.20.1, ChimeraX v1.3, COOT v0.9.8.7.
• Molecular modeling: Python3, Maestro v2021, CHARMM-GUI, Q v5, GROMACS v2022.2, PRIME, LigPrep, ffld_server, Deeptime, GetContacts, SciPy, UCSF ChimeraX v.1.5, PyMOL v2.5.

For manuscripts utilizing custom algorithms or software that are central to the research but not yet described in published literature, software must be made available to editors and reviewers. We strongly encourage code deposition in a community repository (e.g. GitHub). See the Nature Portfolio guidelines for submitting code & software for further information.

Data

Policy information about availability of data

All manuscripts must include a data availability statement. This statement should provide the following information, where applicable:

- Accession codes, unique identifiers, or web links for publicly available datasets
- A description of any restrictions on data availability
- For clinical datasets or third party data, please ensure that the statement adheres to our policy

• Cryo-EM structures were deposited in the Protein Data Bank (https://www.rcsb.org), with the corresponding PDB accession codes: 9LWC and 9LW5.
• The source data is available from the corresponding authors upon reasonable request.

Research involving human participants, their data, or biological material

Policy information about studies with human participants or human data. See also policy information about sex, gender (identity/presentation), and sexual orientation and race, ethnicity and racism.

Reporting on sex and gender	N/A
Reporting on race, ethnicity, or other socially relevant groupings	N/A
Population characteristics	N/A
Recruitment	N/A
Ethics oversight	N/A

Note that full information on the approval of the study protocol must also be provided in the manuscript.

Field-specific reporting

Please select the one below that is the best fit for your research. If you are not sure, read the appropriate sections before making your selection.

☒ Life sciences ☐ Behavioural & social sciences ☐ Ecological, evolutionary & environmental sciences

For a reference copy of the document with all sections, see nature.com/documents/nr-reporting-summary-flat.pdf

Life sciences study design

All studies must disclose on these points even when the disclosure is negative.

Sample size	• In vitro assays: Sample size was not predetermined. For cAMP assays, at least three independent experiments were performed after initial screening, with each of the concentrations in duplicates or triplicates. For BRET experiments, ≥3 independent experiments were carried out for estimates of drug potency and efficacy, which we have found to be sufficient to account for technical (e.g., well loading) and biological (e.g., protein expression) variability (Avet et al., 2022). Sample size was chosen to allow for clear discrimination of effect (or lack thereof) given between-sample variance.
Data exclusions	No data exclusion
Replication	• In vitro experiments were replicated in n independent runs as described in the methods section, figure legends or tables.
Randomization	• No randomization was performed.
Blinding	• cAMP assays, BRET assays: No blinding was performed.

Behavioural & social sciences study design

All studies must disclose on these points even when the disclosure is negative.

Study description	N/A
Research sample	N/A
Sampling strategy	N/A
Data collection	N/A
Timing	N/A
Data exclusions	N/A
Non-participation	N/A
Randomization	N/A

Ecological, evolutionary & environmental sciences study design

All studies must disclose on these points even when the disclosure is negative.

Study description	N/A
Research sample	N/A
Sampling strategy	N/A
Data collection	N/A
Timing and spatial scale	N/A
Data exclusions	N/A
Reproducibility	N/A
Randomization	N/A
Blinding	N/A

Did the study involve field work? ☐ Yes ☒ No

Reporting for specific materials, systems and methods

We require information from authors about some types of materials, experimental systems and methods used in many studies. Here, indicate whether each material, system or method listed is relevant to your study. If you are not sure if a list item applies to your research, read the appropriate section before selecting a response.

Materials & experimental systems

n/a	Involved in the study	
☐	☒	Antibodies
☐	☒	Eukaryotic cell lines
☒	☐	Palaeontology and archaeology
☒	☐	Animals and other organisms
☒	☐	Clinical data
☒	☐	Dual use research of concern
☒	☐	Plants

Methods

n/a	Involved in the study	
☒	☐	ChIP-seq
☒	☐	Flow cytometry
☒	☐	MRI-based neuroimaging

Antibodies

Antibodies used	Fluorescein-conjugated anti-HA-antibody.
Validation	The former antibody has been validated by GenScript (cat. no. A01621).

Eukaryotic cell lines

Policy information about cell lines and Sex and Gender in Research

Cell line source(s)	• CHOk1 cells were obtained from ATCC.<br>• HEK239A cells were obtained from ATCC.<br>• EXPI293F cells were obtained from Thermo Fisher Scientific.
Authentication	• HEK293 cells were authenticated by the supplier by STR profiling.<br>• None of the used cell lines were authenticated by us.<br>• Expression of wild-type or mutants after transfection was authenticated by antiHA immunofluorescence staining of the N-terminal HA-tag in flow cytometry.
Mycoplasma contamination	• Cells were routinely tested for mycoplasma contamination (MycoAlert Mycoplasma Detection Kit). Cells did not test positive for mycoplasma over the course of the experiments.
Commonly misidentified lines<br>(See ICLAC register)	• No commonly misidentified cell lines were used in this study.

Palaeontology and Archaeology

Specimen provenance	N/A
Specimen deposition	N/A
Dating methods	N/A

☐ Tick this box to confirm that the raw and calibrated dates are available in the paper or in Supplementary Information.

Ethics oversight	N/A

Note that full information on the approval of the study protocol must also be provided in the manuscript.

Clinical data

Policy information about clinical studies

All manuscripts should comply with the ICMJE guidelines for publication of clinical research and a completed CONSORT checklist must be included with all submissions.

Clinical trial registration	N/A
Study protocol	N/A
Data collection	N/A
Outcomes	N/A

Dual use research of concern

Policy information about dual use research of concern

Hazards

Could the accidental, deliberate or reckless misuse of agents or technologies generated in the work, or the application of information presented in the manuscript, pose a threat to:

No	Yes	
☒	☐	Public health
☒	☐	National security
☒	☐	Crops and/or livestock
☒	☐	Ecosystems
☒	☐	Any other significant area

Experiments of concern

Does the work involve any of these experiments of concern:

No	Yes	
☒	☐	Demonstrate how to render a vaccine ineffective
☒	☐	Confer resistance to therapeutically useful antibiotics or antiviral agents
☒	☐	Enhance the virulence of a pathogen or render a nonpathogen virulent
☒	☐	Increase transmissibility of a pathogen
☒	☐	Alter the host range of a pathogen
☒	☐	Enable evasion of diagnostic/detection modalities
☒	☐	Enable the weaponization of a biological agent or toxin
☒	☐	Any other potentially harmful combination of experiments and agents

Plants

Seed stocks	N/A
Novel plant genotypes	N/A
Authentication	N/A

ChIP-seq

Data deposition

☐ Confirm that both raw and final processed data have been deposited in a public database such as GEO.

☐ Confirm that you have deposited or provided access to graph files (e.g. BED files) for the called peaks.

Data access links<br>*May remain private before publication.*	N/A
Files in database submission	N/A
Genome browser session<br>(e.g. UCSC)	N/A

Methodology

Replicates	N/A
Sequencing depth	N/A
Antibodies	N/A
Peak calling parameters	N/A
Data quality	N/A
Software	N/A

Flow Cytometry

Plots

Confirm that:

☐ The axis labels state the marker and fluorochrome used (e.g. CD4-FITC).

☐ The axis scales are clearly visible. Include numbers along axes only for bottom left plot of group (a 'group' is an analysis of identical markers).

☐ All plots are contour plots with outliers or pseudocolor plots.

☐ A numerical value for number of cells or percentage (with statistics) is provided.

Methodology

Sample preparation	CHO-K1 cells were transfected 24 h prior the sample preparation. The cells were collected and then washed twice with PBS followed by fixation by 4% PFA in PBS (15 minutes in room temperature, occasional mixing). The cells were washed twice with PBS, and suspended in blocking buffer 1% BSA in PBS + 0.1% Tween 20, followed by 30 min incubation at room temperature. Cells were then incubated with the fluorescein-conjugated anti-HA-antibody (GenScript, cat. no. A01621), diluted in the blocking buffer at 2 ug/ml, and the suspension was incubated 1 h on a shaker in darkness. The cell suspension was washed three times with PBS, and the fluorescence (λex=488 nm, λem=525/30 or 533/30 nm) was measured in flow cytometry.
Instrument	Guava® easyCyte™ (Merck Millipore), or BD Accuri™ C6 (BD Biosciences).
Software	Guava® InCyte software, or BD Accuri™ C6 Plus Software.
Cell population abundance	The starting population was > 70% of the total events, and the final sorted population was 0.01% - 33.2% of the single cells in the starting population.
Gating strategy	Gating as follows:<br>Starting population: FCS-A/SSC-A<br>Single cells: FCS-A/FCS-H, linear range<br>FITC-positive cells: FCS-A/FITC-A<br>Mock cells (negative control, used for comparison for FITC-positive cells) always included in experiments.

☐ Tick this box to confirm that a figure exemplifying the gating strategy is provided in the Supplementary Information.

Magnetic resonance imaging

Experimental design

Design type	N/A
Design specifications	N/A
Behavioral performance measures	N/A

Acquisition

Imaging type(s)	N/A
Field strength	N/A
Sequence & imaging parameters	N/A
Area of acquisition	N/A

Diffusion MRI ☐ Used ☒ Not used

Preprocessing

Preprocessing software	N/A
Normalization	N/A
Normalization template	N/A

Noise and artifact removal | N/A

Volume censoring | N/A

Statistical modeling & inference

Model type and settings | N/A

Effect(s) tested | N/A

Specify type of analysis: ☐ Whole brain ☐ ROI-based ☐ Both

Statistic type for inference (See Eklund et al. 2016) | N/A

Correction | N/A

Models & analysis

n/a	Involved in the study
☒	☐ Functional and/or effective connectivity
☒	☐ Graph analysis
☒	☐ Multivariate modeling or predictive analysis
