# Supplementary material for: Molecular mechanisms of native ligand selectivity in catecholamine G protein-coupled receptors
Source: Nat Commun. 2026 Apr 23;17:4112. doi: 10.1038/s41467-026-71361-8 (PMC13150006; doi:10.1038/s41467-026-71361-8)
Supplement: Supplementary file 6 — Transparent Peer Review file [file 41467_2026_71361_MOESM6_ESM.pdf]

nature portfolio

Peer Review File

Molecular mechanisms of native ligand selectivity in catecholamine G protein-coupled receptors

Corresponding Author: Professor Jens Carlsson

This file contains all reviewer reports in order by version, followed by all author rebuttals in order by version.

Version 0:

Reviewer comments:

Reviewer #1

(Remarks to the Author)
Remarks to the Authors

This study by Kahlous, Rinne, Zhang, et al. elucidates the molecular mechanisms governing GPCR selectivity through an integrative approach combining sequence analysis, structural biology, and pharmacology. Using β2 and D1 receptors, the authors successfully swapped ligand selectivity by introducing mutations at both orthosteric and allosteric sites. Notably, both types of mutations were required to alter selectivity, a mechanism conserved within the same GPCR subfamily but not transferable across other catecholamine receptor families. These mutation hotspots were identified through sequence alignments, and receptor functionalities (selectivity and potency) were further characterized using pharmacological assays. The authors also supported their findings with cryo-EM structures of mutated β2 and D1 receptors (with dopamine for β2 and adrenaline for D1). Structural analyses, complemented by AlphaFold and molecular dynamics (MD) simulations, corroborated these findings and provided insights into how mutations at allosteric sites, distant from the orthosteric site, together with orthosteric mutations, induce conformational changes that support the observed pharmacological activity. The authors' exploration of the evolutionary implications for GPCR selectivity is particularly compelling.

I recommend publication after addressing few issues outlined below.

Comments

1. Unclear sentence: "N312V, V117S, and F193S at positions 3x36, 7x38, and 45x52, respectively, M1-3".
Did the authors intend to write "V117S, F193S, and N312V at positions 3x36, 45x52, and 7x38, respectively, M1-3"?
It would be helpful to clarify the order and numbering of mutations and positions for accuracy.

2. Inconsistency in pEC50 values: there are discrepancies between pEC50 values reported in the manuscript and those in "filedata1", particularly for logEC50 values. For example:
Manuscript -> "The combination of the N312V and V117S mutations (M4) resulted in 7- and 9-fold selectivity for dopamine over adrenaline and noradrenaline, respectively. In this case, adrenaline (pEC50 = 5.13) and noradrenaline (pEC50 = 4.96) showed a 4700- and 120-fold loss of potency, respectively, whereas the potency of dopamine (pEC50 = 5.95) was comparable to that obtained at the wild-type β2R (Figure 3A)."

Filedata1->
β2 M4 logEC50 [DA] = -5.86
β2 M4 logEC50 [A] = -4.88
β2 M4 logEC50 [NA] = -4.91

If logEC50 and pEC50 values are not equivalent, I recommend including additional tables in the manuscript with pEC50 values (and fold changes) to ensure consistency with supplementary data. It would be appreciated the alignment of all values across the manuscript and supplementary information.

3. Unclear sentence: “in the case of the β2R, and the selectivity profile of the D1R mutant was not fully switched because noradrenaline remained more potent than dopamine”
Did the authors mean “in the case of the β2R, and the selectivity profile of the D1R mutant was not fully switched because dopamine remained more potent than noradrenaline”?

4. Cryo-EM analysis
Although the cryo-EM structures are of high quality with good resolution, have the authors considered re-extracting particles with a larger box size after removing junk particles via Heterogeneous Refinement (before final non-uniform refinement)? Re-extraction after 3D alignments could improve particle centering, and higher sampling would enhance the achievable Nyquist frequency (resolution limit).
Additionally, some structural features in the “map_sharp.mrc” files exhibit fragmented density. Would electron density appear more continuous in the “map.mrc” files? Including an orientation diagnostic analysis (e.g., SCF score and plots from an Orientation Diagnostic job) could strengthen the analysis. To further optimize the binding site analysis (including the ligand density), the authors would benefit in considering “rebalance orientations” and “subsetting particles by statistics” jobs. Removing low-quality or uninformative particles via Rebalance or Subset Particles by Statistics could enhance reconstruction quality.

Reviewer #2

(Remarks to the Author)
In the manuscript “Molecular mechanisms of ligand selectivity by catecholamine G protein-coupled receptors”, the authors provide insight into the close (evolutionary) relationship between catecholamine receptors, specifically the D1 dopamine and β2 adrenergic receptors. Combining signaling studies, computational and bioinformatic analyses, and cryoEM, the study uncovers critical hotspots in the receptors that determine ligand selectivity. The reported findings are intriguing and highlight how even residues that do not directly interact with the orthosteric ligands contribute to pharmacological selectivity. Overall, the data is largely convincing but the study has some weaknesses that need to be addressed:

Major
• While the β2R-M16 structure is of pretty good quality and definitely supports the conclusions, the D1R-M74 is rather poor and insufficient to back up the proposed orthosteric ligand binding pose. The density for adrenaline is rather ambiguous, which is evidenced by a strong clash between the D3.32 sidechain and adrenaline’s hydroxyl group attached to the ethylamine moiety of the ligand. Even the sidechain of N292 appears to be too close to one of the catecholamine hydroxyls. The proposed binding pose of adrenaline in relationship to the aspartate is therefore unlikely and either the sidechain, the ligand, or both need to be adjusted. This is also reflected in a comparatively high clashscore of nearly 12 for the structure. This structure definitely needs more work before publication.

Minor
• The authors show expression levels for effectively all mutants discussed in this manuscript, which is something not everyone does. That being said, it raises questions about the interpretation of max efficacies for some of the mutants based on total cellular cAMP levels, as some mutants have significantly reduced or altered expression levels. For instance, β2R-M3 and β2R-M4 are both reported with considerably reduced efficacies in Figure 3A, but their expression levels in Figure S4 appear to be reduced by at least as much. That would indicate that these mutants potentially do not affect receptor efficacy per se, but on page 10 the authors state that M3 does reduce potency and efficacy. The expression levels therefore need to be factored into the analysis as it pertains to effects of mutants on total receptor signaling output. That being said, I do think that this is not a concerning issue since potencies appear largely the driver of selectivity in this study.

• There are also other smaller issues related to the structure. The reported model B-factors (while not really that important in cryoEM) of the β2R-M16 structure are almost certainly incorrect as they are below 10 for both protein and ligand. Ligand density in Figure S12 is indicated as “Ligand” rather than the name of the compound, and there is no label in Figure S11. The processing schematic in both S11 and S12 is confusing since the authors appear to gain particles during 3D classification, i.e. the particle stack following 2D classification or an initial 3D reconstruction is smaller than the final stack size.

• It is rather curious that the M16 mutation set appears to increase basal activity in b2AR and b1AR, but not b3AR. I wonder if the authors could comment on this and why they think these mutations increase basal activity in the first place.

• The authors discuss b2AR and D1DR structures on pages 7 and 8, but the references to this work is missing in this section.

• Figure 4 is very overwhelming, and I wonder if this can be split across two figures to make it more palatable for readers. Panels A and B also lack dashed lines for the ionic interactions between the ligands and D3.32.

• The authors need to check the construct mutations in Table S1 as both structural constructs appear to list a S107V mutant, which is implausible.

• It is not explicitly stated whether the cAMP assays were performed in HEK or CHO cells.

Reviewer #3

(Remarks to the Author)
Summary

In this manuscript, Kahlous et al. performed an extensive investigation of the molecular determinants of (endogenous) ligand selectivity in catecholamine GPCRs, focusing on β2-adrenergic and D1 dopamine receptors. By combining sequence analysis, mutagenesis, functional assays, cryo-EM structures, and computational simulations, the authors propose that a small set of residues can govern selective ligand recognition and that exchanging these residues can swap receptor selectivity profiles.

While the study addresses an important question with broad implications for receptor biology and drug design, several conceptual, methodological, and interpretive issues need to be addressed before this manuscript can be considered for publication. In particular, the rationale for mutant selection, the interpretation of functional assays, and the link between structural/computational observations and pharmacological outcomes require further clarification. Additionally, key methodological details are missing, some conclusions are overstated or inconsistent with the data, and there are several points where the presentation could be improved for clarity and reproducibility.

Major comments

- The logic linking bioinformatics predictions to mutagenesis is unclear. If five hotspots were identified, what guided the selection of the additional 120 receptor mutants? It appears the experimental strategy was more exploratory than hypothesis-driven, which is valid but should be acknowledged. Present the narrative accordingly rather than implying a strictly rational design.

- Given the complexity of GPCR signaling (i.e., modalities differences, pathway biases, etc.), using functional assays to assess selectivity can confound interpretation, as signaling outputs may reflect changes in downstream pathway dynamics rather than true differences in ligand binding preferences. Furthermore, functional assays such as cAMP accumulation are susceptible to downstream amplification, complicating the interpretation of ligand selectivity. This likely explains the rather complex effects described in section “Binding site mutations reduce selectivity at the expense of efficacy and potency (P10-12)” and it further supported by the more modest selectivity profiles observed for M16 and M74 at the Gs recruitment assay, indicating that mutants are altering overall receptor signaling mechanisms rather than ligand recognition (and selectivity) motifs. Alternative approaches (e.g., binding affinity assays without overexpressed G proteins) should be used for the key mutants (including the single residue binding site mutants) to properly validate the claims on selectivity shifts.

- Several mutants display increased basal activity or altered signaling profiles (e.g., Figs 3C and 3E), potentially indicating changes in constitutive activity rather than true selectivity shifts. These observations should be explicitly addressed and controlled for.

- Some apparent selectivity "switches" are actually loss-of-function across ligands, which does not support the claim of reciprocal ligand selectivity or the direct role of those residue positions as selectivity hotspots. Also, since residue positions in M16 and M74 do not overlap (apart from 3x36 and 7x38) and “are not transferable”, they cannot be considered selectivity determinants, as these receptor variants are incomparable to one another and to their wild types.

- The conclusion that this work enables rational ligand design is not supported by the presented data. The distinction between determinants of endogenous agonist selectivity vs pharmacological ligand selectivity should be clarified. In this regard, the manuscript title is misleading.

- The phylogenetic analysis in Fig.1A could support a narrative where D1-like and β receptors share ancestral G protein coupling while diverging in ligand specificity—this should be discussed as it provides important context for the study’s main claim. Moreover, the manuscript implies that ligand selectivity evolved independently of G protein preference, but this should be discussed in more detail, given evidence that Gs-coupling emerged earlier and was retained in catecholamine receptor evolution (c.f. https://pubs.acs.org/doi/full/10.1021/jm0511031).

- The abstract’s reference to "rational protein engineering" is overstated; the work is more consistent with a semi-rational or empirical approach, particularly given the variety of mutants tested and the limited gain-of-function results.

- Statements such as “selectivity is governed by a few positions within the binding site” contradict experimental data showing either retention of native selectivity or loss of function, not a switch.

- There is no clear link between the section “Selectivity mechanisms in adrenergic, dopaminergic, and serotonergic GPCRs” and the rest of the manuscript. Is this web-based tool based on the evolutionary analysis or the residue positions actually swapped at the D1 and β2? Please, make this link clearer or remove this section, as it does not seem relevant to the present work.

- Why were MD/FEP simulations performed on in silico mutated WT structures rather than the experimental cryo-EM models of the mutants? This reduces confidence in the predicted energetic and structural changes.

- MD and FEP results (e.g., “mutations at positions 7x38 and 3x36 led to a swap of agonist binding modes between the

receptors" c.f. P21) do not align consistently with pharmacological data (e.g., for V117S and N312V), and inferences about ligand amine positioning are not directly assessed. These contradictions need explanation.

- The use of a chimeric β2 structure in an intermediate/inactive-like state is problematic (PDB 4LDO). A better strategy would be to revert mutations in the new cryo-EM structures and simulate these.

- The selection and processing of sequence data for UMAP need to be better justified. Why were 97 sequences used instead of canonical human variants? Which other sequences are these?

- Clarify how the one-hot encoding was performed for UMAP, and justify why PCA was not used instead, since the plot of PCA loadings would provide interpretable dimensions and may highlight subtype differences more clearly.

- Provide supporting data (e.g., BLOSUM62 scores) for the identification of mutational hotspots. Clarify which of these residues were actually mutated, and explain discrepancies.

- AlphaFold3 models offer little added value here; with cryo-EM structures available, focus on the experimental data and simulations.

Minor Comments

- Figure 3 is difficult to interpret. Improve labeling and color coding; include WT curves for reference and display more single mutant curves for clarity.
- Supplementary figures (e.g., S4, S5, S16) lack clear labeling and do not match the text descriptions. Indicate residue identities and transformation directions clearly.
- Page 11: "descried" should be "described."
- Check significant figures and precision of pEC50 values across the manuscript.
- Ensure all receptor subtypes are clearly labeled in figures and graphs (e.g., Figure 3).
- Figure 2 describes the research pipeline and is suitable for a grant application but not for a research paper (e.g., why is β-arrestin included if not assayed in the study?) Instead, use this space the present the results or to illustrate, for example, where the actually mutated residues are located in the receptors and how these residues compare to their counterparts in the other dopamine and adrenergic subtypes (sequence alignments of the 7+4 positions).
- Improve figure captions for S16 and S5 to explain which atom pairs/distances are being measured and the context for those results.
- Missing details on the protein preparation for modeling, including treatment of missing loops and missing side chains, removal of nanobodies/transducer proteins, reversion of thermostabilizing mutations, etc.
- Ensure that Figure 2 and S3/S4 consistently represent the same positions. Currently, highlighted residues and tested mutants do not correspond. Otherwise, explain the discrepancies.
- The definition of binding site residues is unclear and should be explicitly described in the Methods. List the residues.

Reviewer #4

(Remarks to the Author)
In their manuscript, Kahlous et al. study the molecular basis and evolution of receptor selectivity in aminergic receptors using a combination of signalling assays, bioinformatics, computational chemistry, and structural biology. They aimed to switch the selectivity profiles of D1 and β2R while retaining physiological logEC50 values to gain insights into the molecular mechanisms of ligand selectivity.

First, the authors determined the potency and efficacy of adrenaline, noradrenaline, and dopamine at the β2R and D1 receptors using an HTRF-based cAMP assay. They further show that β2R and D1 receptors are phylogenetically more closely related to each other than β2R to the α-adrenergic receptors and D1 to the D2 receptor. This is noteworthy because one would have expected a different result given the pharmacological profiles of the receptors. It would be interesting to know why these results disagree with phylogenetic trees obtained elsewhere (e.g. GPCRdb.org).

A set of residues termed 'selectivity hotspots' was then identified using a combination of sequence alignments and BLOSUM62 scoring, reducing the number of positions differing between D1 and β2R, which need to be primarily considered for residue exchange. Thus, the number of positions was reduced to seven 'selectivity hotspot' positions. Interestingly, some of these residues do not interact with the ligand.

The authors then found that binding site mutations alone could not swap the selectivity profiles of the two receptors. Instead, mutations outside the ligand binding sites were required. In addition, the cAMP response of the mutants with swapped selectivity profiles was tested for histamine, serotonin and acetylcholine, confirming that the mutations did not lead to promiscuity in ligand binding. The selectivity mechanism differed between receptors, as the β2R mutations could not be transferred to D1 and vice versa. Instead, no cAMP response was observed for the resulting constructs. While hotspot positions may differ between receptors, an extended analysis identified 3x36 and 7x38 as frequent hotspots.

This work will be very significant for researchers in the GPCR field aiming to understand molecular mechanisms of selectivity as well as the evolution of GPCR ligand binding and will also be of interest to researchers outside of the GPCR community. The developed web-based tool will help other researchers in their analyses of additional GPCRs.

The manuscript is easy to follow and the applied methods are very well suited for the study. Overall, I very much enjoyed reading the manuscript. I cannot comment on the structure determination, cryo-EM structures, and the computational chemistry methods.

Comments:
- Could the authors provide more details on the multi-species sequence alignments used for the identification of receptor selectivity hotspots? A supplementary table including conservation and BLOSUM62 scoring would be helpful. Please provide a rationale why seven positions were selected. Were these the only residues with >80% conservation and a BLOSUM62 score <0?
- Please provide a rationale for the inclusion/exclusion of neighbouring residues around the hotspots. Why did you choose these residues for residue exchange?
- 'This analysis reduced the number of differences in the alignment from ~100 to seven positions' – consider rephrasing, as the number of differences in the alignment stays the same, but the number of positions considered for further analysis is reduced.
- Please provide additional information on the normalisation of the signalling data to WT expression. How exactly was this normalisation performed? Was WT signalling measured for different expression levels?
- Will the code used in this manuscript be shared, e.g. on GitHub?
- In Figure 3B, a graph for WT β2R and D1R, an additional label specifying the mutant (e.g. M3 for the N312V mutant of β2R), and a matching size of the graphs would help compare the effect of mutations.
- 'Dose-response curve' and 'concentration-response curve' are used interchangeably in the manuscript. Consider using 'concentration-response curve' throughout.
- Consider stating the error of the cAMP pEC50 determination in the main text
- Abbreviations should be explained when they are used for the first time (please double check for PDL and PEI)
- Please specify the type of PEI used (linear vs branched, manufacturer)
- Please detail the expression conditions in the 'Structural biology experiments' section. How much DNA and PEI were used? Which type of PEI was used and where was it purchased?
- In the 'Structural biology experiments' section, the source/manufacturer for Expi293F cells, PEI, DDM, LMNG, M1-FLAG, CHS are missing. Which media were used? Were the cells lysed before solubilisation and if yes, how? What was the concentration of the detergents? The buffer used for solubilisation is missing. How was the detergent exchanged? How much of which buffer was used? Which SEC column was used? What was the final concentration of the receptor constructs after concentration? What was the concentration of the ligands? Please state the methods for the purification of Gβγ, Nb35 and scFv16 or cite the relevant literature.
- Please double check the following sentence: 'Free energy perturbation calculations were then performed using the software Q72 **using and** its OPLS all-atom force field version'

Reviewer #5

(Remarks to the Author)
Kalhlous et al. studies target-selectivity of catecholamines in important drug targets, human b2 adrenergic and D1 dopamine receptors. The manuscript addresses a long-standing question in the field of how highly similar small molecules activate their corresponding receptors with high selectivity. The authors identify a small set of residues responsible for ligand selectivity. These residues were not only in the orthosteric pocket but also outside this site. The authors show that by exchanging 4-7 amino acids between the b2 adrenergic and D1 dopamine receptors, the selectivity profile between them is swapped. Kahlous et al then follow up by solving the 3D structure of the mutant receptors with cryo-EM and analyzing the activation motifs of class A GPCRs with MD simulations. Employing a range of experimental and computational methods, the authors provide molecular insights into the selectivity of two drug targets with high pharmacological relevance. The result is highly useful and relevant for the field, since it directly contributes to the development of safer therapeutics that can be achieved via designing selective orthosteric ligands. Therefore, I recommend this manuscript for publication at Nature Communications. However, the following major and minor points should be addressed before publication:

Major Remarks:
1. Throughout the manuscript, the term "selectivity" is used without any clarification. The authors should clarify
A) Which selectivity is calculated – Is it potency selectivity?
B) Which potency values were used for these calculations? This is indeed very vague in the manuscript, as the authors have used two different assays, G protein recruitment and cAMP assays, to determine the potency and efficacy of the receptor. Therefore, it is crucial to mention which potency values were used.
2. Figure 2 and S3. It is not clear if the authors intentionally focused on more or less the upper half of the receptors to identify the residues involved in determining selectivity, or what is shown in these figures is the result of a global analysis.
3. Figure S4. Did the authors consider any cell surface expression threshold to decide whether or not to consider a mutation? For instance, M9 at S4.B shows a >70% reduction in the cell surface. Was this mutation considered regardless?
4. Figure 3. While all the CRCs start from the zero point of the plot, in the C part –cMAP response, the curve starts at ~30%. The authors should explain the reason. Were the cAMP responses normalized to the vehicle response? If so, why in the C part, the curve does not start from 0?
5. To have a deeper understanding of the molecular mechanism that governs the selectivity, the authors should run MD simulations for the receptors with mutations in the binding pocket and the outside ones separately and compare the results with the current system, which has both site mutations. These would allow for a better understanding of the combinatorial role that these two sites play and the process that leads to the rescue of potency and efficacy, while maintaining the selectivity.
6. Kahlous et al propose two distinct mechanisms contributing to switching receptor selectivity. They propose that in the

b2R, TM3/TM4/TM5 interface connects the ligand binding site to microswitches that control conformational changes and mutations that increase the receptor response to agonist binding, resulting in potencies closer to physiological concentrations. The second mechanism, i.e., for D1R, they hypothesize that residues in the TM2-TM7 interface, which surround the orthosteric ligand binding pocket, are critical for the switch in the selectivity profile of D1R. To prove their hypothesis on the allosteric effect of the second region on the potency and efficacy rescue in the orthosteric pocket, the authors should run network analysis for the WT and mutant systems (orthosteric pocket only, second region only, and both sites together) to examine whether the residue movements in these areas are correlated. If the mechanism is conserved, as claimed by the authors, this is expected to be reflected in this analysis.

7. In the discussion section, authors write that "Unexpectedly, mutations in the binding site primarily led to a loss of response to the native agonist, rather than switching the selectivity profiles and increasing the potency of the new ligand.". Indeed, there are numerous studies in the literature that describe the detrimental effect of mutations in the orthosteric pocket of class A GPCRs on affinity, potency, and efficacy in different cell-based assays. This is actually quite expected, as mutations in this site change the profile of the pocket geometrically and chemically, influencing the binding of a given ligand. Authors should argue why this was unexpected to them.

Minor Remarks:

1. Figure 4. The colors picked for the b2-adrenergic receptor in complex with adrenaline and dopamine are very close, hindering pose comparison. I recommend using more distinct colors.

2. Figure S14 and S15. The authors are encouraged to follow a consistent labelling. It would be better to add "Cryo-EM" to the label "D1R-WT-dopamine" in S14, similar to S15.

Version 1:

Reviewer comments:

Reviewer #1

(Remarks to the Author)
My concerns have been addressed. Congratulations to the authors.

(Remarks on code availability)

Reviewer #2

(Remarks to the Author)
All my requests have been satisfyingly addressed, and the quality of the D1R-M74 structure is much improved. I recommend this manuscript for immediate publication.

(Remarks on code availability)
N/A

Reviewer #3

(Remarks to the Author)
I thank the authors for the extensive work done in response to my comments. They've clearly put great effort into addressing the points I raised, both in terms of new analyses and clarifying the presentation of the study. I appreciate the thoughtful and thorough responses, and I'm satisfied that all concerns have been adequately addressed. I recommend the manuscript for publication.

(Remarks on code availability)

Reviewer #4

(Remarks to the Author)
I have reviewed the author's responses and changes to the manuscript. The authors have addressed my comments and questions. I do not have any additional comments or suggestions.

(Remarks on code availability)

Reviewer #5

(Remarks to the Author)

Dear Editor,

I have carefully evaluated the revised manuscript by Kahlous et al. and the authors’ responses to my comments. All of my major and minor points have been adequately addressed, and the revisions have strengthened the manuscript.

I am satisfied with the current version and support its publication in the journal.

Sincerely,
Aida Shahraki

(Remarks on code availability)

Open Access This Peer Review File is licensed under a Creative Commons Attribution 4.0 International License, which permits use, sharing, adaptation, distribution and reproduction in any medium or format, as long as you give appropriate credit to the original author(s) and the source, provide a link to the Creative Commons license, and indicate if changes were made.
In cases where reviewers are anonymous, credit should be given to 'Anonymous Referee' and the source.
The images or other third party material in this Peer Review File are included in the article’s Creative Commons license, unless indicated otherwise in a credit line to the material. If material is not included in the article’s Creative Commons license and your intended use is not permitted by statutory regulation or exceeds the permitted use, you will need to obtain permission directly from the copyright holder.
To view a copy of this license, visit https://creativecommons.org/licenses/by/4.0/

December 18th, 2025

We want to thank the five referees for taking the time to review our manuscript (*"Molecular mechanisms of ligand selectivity by catecholamine G protein-coupled receptors"*, Manuscript: NCOMMS-25-52261-T) and the many constructive suggestions. We have carefully considered all the reviewers' comments and performed additional experiments and calculations, resulting in a substantially stronger manuscript. Please find below point-by-point responses to the referees with their comments in ***Blue***. Relevant changes to the manuscript have been marked in yellow to facilitate the review process.

Reviewer #1

The first reviewer appreciated our work: ***"The authors' exploration of the evolutionary implications for GPCR selectivity is particularly compelling"***. The reviewer recommended publication after addressing a few minor comments:

1. Reviewer: ***"Unclear sentence: "N312V, V117S, and F193S at positions 3x36, 7x38, and 45x52, respectively, M1-3". Did the authors intend to write "V117S, F193S, and N312V at positions 3x36, 45x52, and 7x38, respectively, M1-3"? It would be helpful to clarify the order and numbering of mutations and positions for accuracy."***

We thank the reviewer for identifying this inconsistency, which we have corrected in the revised manuscript (page 11). The order and numbering of mutants is available in Supplementary Datafile 1, which now is mentioned on page 10.

2. Reviewer: ***"Inconsistency in pEC50 values: there are discrepancies between pEC50 values reported in the manuscript and those in "filedata1", particularly for logEC50 values.***

For example:
Manuscript -> "The combination of the N312V and V117S mutations (M4) resulted in 7- and 9-fold selectivity for dopamine over adrenaline and noradrenaline, respectively. In this case, adrenaline (pEC50 = 5.13) and noradrenaline (pEC50 = 4.96) showed a 4700- and 120-fold loss of potency, respectively, whereas the potency of dopamine (pEC50 = 5.95) was comparable to that obtained at the wild-type β2R (Figure 3A).

Filedata1->
β2 M4 logEC50 [DA] = -5.86
β2 M4 logEC50 [A] = -4.88
β2 M4 logEC50 [NA] = -4.91

If logEC50 and pEC50 values are not equivalent, I recommend including additional tables in the manuscript with pEC50 values (and fold changes) to ensure consistency with supplementary data. It would be appreciated the alignment of all values across the manuscript and supplementary information."

We thank the reviewer for identifying these inconsistencies between the main manuscript and supplementary information. We have carefully checked all the data and the numbers have been updated in the revised manuscript. We did not highlight these changes in the revised manuscript.

3. Reviewer: ***"Unclear sentence: "in the case of the β2R, and the selectivity profile of the D1R mutant was not fully switched because noradrenaline remained more potent than dopamine" Did the authors mean "in the case of the β2R, and the selectivity profile of the D1R mutant was not fully switched because dopamine remained more potent than noradrenaline"?"***

We thank the reviewer for identifying this error and we have corrected this sentence in the revised manuscript (page 13).

4. Reviewer: ***"Although the cryo-EM structures are of high quality with good resolution, have the authors considered re-extracting particles with a larger box size after removing junk particles via Heterogeneous Refinement (before final non-uniform refinement)? Re-extraction after 3D alignments could improve particle centering, and higher sampling would enhance the achievable Nyquist frequency (resolution limit). Additionally, some structural features in the "map_sharp.mrc" files exhibit fragmented density. Would electron density appear more continuous in the "map.mrc" files? Including an orientation diagnostic analysis (e.g., SCF score and plots from an Orientation Diagnostic job) could strengthen the analysis. To further optimize the binding site analysis (including the ligand density), the authors would benefit in considering "rebalance orientations" and "subsetting particles by statistics" jobs. Removing low-quality or uninformative particles via Rebalance or Subset Particles by Statistics could enhance reconstruction quality."***

We thank the reviewer for these suggestions. We have performed an extensive reprocessing of the cryo-EM data for the D_1R (M74)–adrenaline–miniG$_{siN}$–G$\beta_1\gamma_2$–Nb35 complex to improve cryo-EM density map quality. Following the reviewer's suggestion, the box size was increased from 256 px to 320 px, and multiple rounds of *ab initio* reconstruction and heterogeneous refinement were conducted to remove junk particles. Additionally, the 'Rebalance Orientations' tool was applied to mitigate effects of preferred orientation. These steps significantly improved the final reconstruction, yielding a cryo-EM density map with more uniform angular distribution and enhanced density quality—particularly in the ligand-binding pocket—which supports a more reliable structural interpretation (See Figure R1 below). We have updated the manuscript with these new results and provide new maps and validation reports.

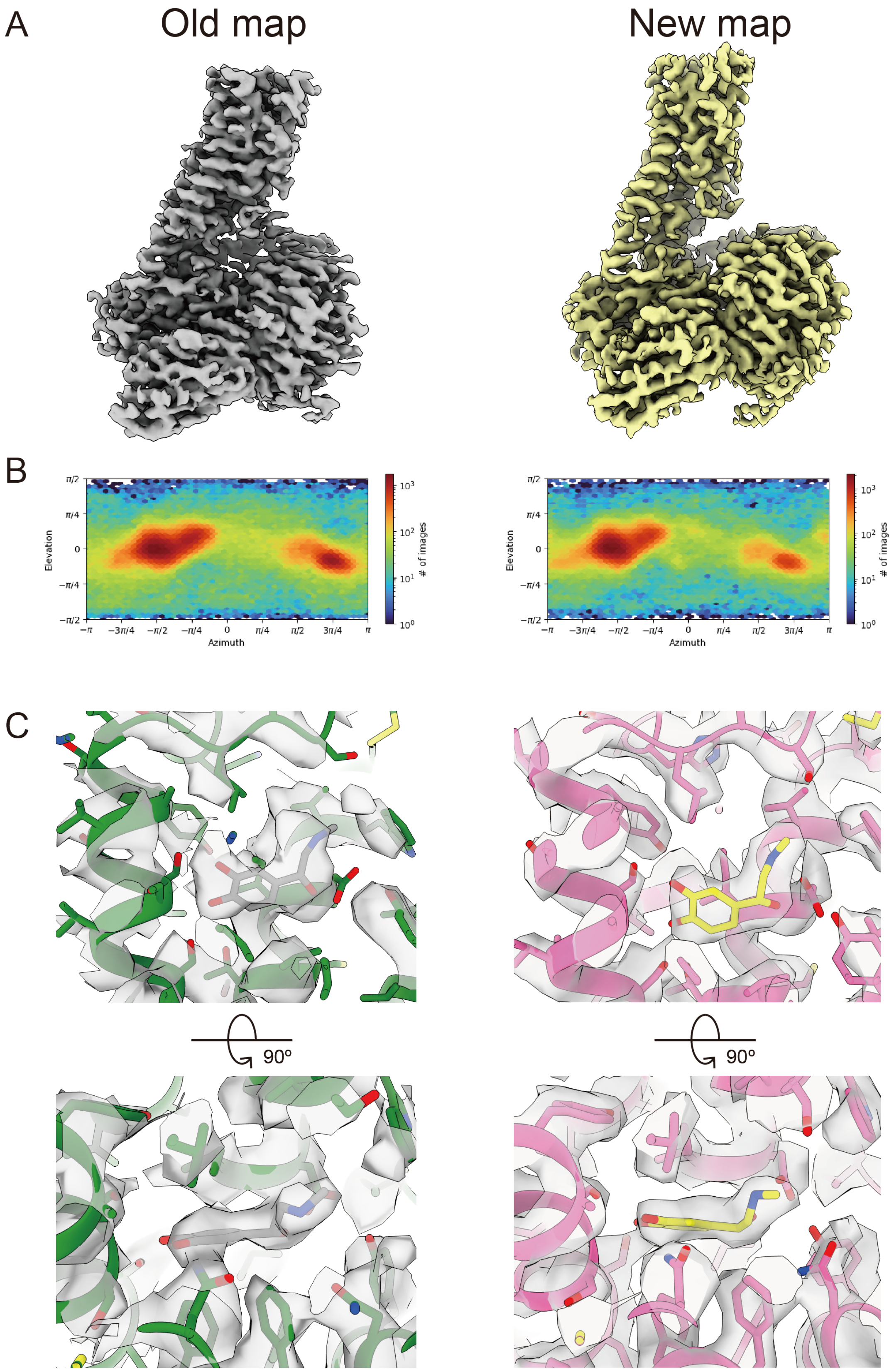

Figure R1. Reprocessed cryo-EM data. Overall cryo-EM density map (A), viewing direction distribution (B) and cryo-EM density map of the orthosteric binding pocket (C) of D_1R (M74) bound to adrenaline in previous and revised manuscript.

Reviewer #2

The second reviewer appreciated the key results of our work: ***"The reported findings are intriguing and highlight how even residues that do not directly interact with the orthosteric ligands contribute to pharmacological selectivity"***. The reviewer also found that ***"the data is largely convincing"***. The reviewer requested additional refinement of the cryo-EM structure and also had a few minor comments, which we address below:

Major comments

1. Reviewer: ***"While the β2R-M16 structure is of pretty good quality and definitely supports the conclusions, the D1R-M74 is rather poor and insufficient to back up the proposed orthosteric ligand binding pose. The density for adrenaline is rather ambiguous, which is evidenced by a strong clash between the D3.32 sidechain and adrenaline's hydroxyl group attached to the ethylamine moiety of the ligand. Even the sidechain of N292 appears to be too close to one of the catecholamine hydroxyls. The proposed binding pose of adrenaline in relationship to the aspartate is therefore unlikely and either the sidechain, the ligand, or both need to be adjusted. This is also reflected in a comparatively high clashscore of nearly 12 for the structure. This structure definitely needs more work before publication."***

We thank the reviewer for the suggestion. We have reprocessed the cryo-EM data for D_1R (M74)–adrenaline–miniG$_{siN}$–G$\beta_1\gamma_2$–Nb35 complex, which has resulted in a significantly improved cryo-EM density map (as shown in Figure R1). The new map provides clear and continuous density for the adrenaline ligand, enabling a more accurate and reliable model building. The clash score has decreased from 12 to 8. Importantly, the steric conflicts for key residues, including D103$^{3.32}$ and N292^{6x55}, have been resolved in the current model, which now depicts a more physiologically plausible binding pocket. We have updated the manuscript with these new results and provide new maps and new validation reports.

Minor comments

2. Reviewer: ***"The authors show expression levels for effectively all mutants discussed in this manuscript, which is something not everyone does. That being said, it raises questions about the interpretation of max efficacies for some of the mutants based on total cellular cAMP levels, as some mutants have significantly reduced or altered expression levels. For instance, β2R-M3 and β2R-M4 are both reported with considerably reduced efficacies in Figure 3A, but their expression levels in Figure S4 appear to be reduced by at least as much. That would indicate that these mutants potentially do not affect receptor efficacy per se, but on page 10 the authors state that M3 does reduce potency and efficacy. The expression levels therefore need to be factored into the analysis as it pertains to effects of mutants on total receptor signaling output. That being said, I do think that this is not a concerning issue since potencies appear largely the driver of selectivity in this study."***

We thank the reviewer for these important observations regarding the expression of mutant receptors and efficacy. We also agree with the reviewer that the driver of

selectivity is differences in potency, which would not be substantially affected by the variation in expression observed in our study. According to any receptor model with response amplification (see for instance Kenakin, Pharmacologic Analysis of Drug-Receptor Interaction, Raven Press, 1997), the theoretical maximum response (set by the downstream signalling cascades) is obtained when the agonist binding and receptor activation reach a certain threshold. If the receptor amount, the binding affinity or the intrinsic efficacy are too low, the response obtained is smaller than the theoretical maximum. The responses obtained for most receptor mutants/ligands follow this; *i.e.* none of the three agonists had different maximum response for any significant receptor mutant (Supplementary datafile 1). Thus, there is no reason to assume that the expression levels affect our conclusions. The inherent properties of the mutants are likely the main driver of the switched selectivity profiles, and the key mutants (β_2R-M16 and D_1R-M74) with switched selectivity profiles exhibit expression levels comparable to those of the wild-type receptors. In addition, we complemented the cAMP measurements, which can be subject to signal amplification, with G protein recruitment assays (minimal amplification), and the results from the two assays are consistent. In the revised manuscript, we have calculated the statistical significance for differences in E_{max} between ligands for each receptor mutant in supplementary datafile 1. We also note in the results section that the selectivity profiles of key mutants are not affected by expression levels (page 10), and that the results obtained for β_2R-M4 may be affected by reduced expression (page 11).

3. Reviewer: ***"There are also other smaller issues related to the structure. The reported model B-factors (while not really that important in cryoEM) of the β2R-M16 structure are almost certainly incorrect as they are below 10 for both protein and ligand. Ligand density in Figure S12 is indicated as "Ligand" rather than the name of the compound, and there is no label in Figure S11. The processing schematic in both S11 and S12 is confusing since the authors appear to gain particles during 3D classification, i.e. the particle stack following 2D classification or an initial 3D reconstruction is smaller than the final stack size."***

We thank the reviewer for the suggestion and apologize for the oversight in the initial manuscript. The relevant values in Table S3 have been corrected accordingly. In Figures S9 and S10, the ligands have been clearly labelled as dopamine or adrenaline, respectively. Since the initial model was generated using a subset of particles selected from 2D classification, which was subsequently used for heterogeneous refinement against the entire dataset, the number of particles obtained after refinement is larger than that used in the initial 3D reconstruction. To avoid potential confusion, we have removed the number of particles used for the initial 3D reconstruction from the revised figure.

4. Reviewer: ***"It is rather curious that the M16 mutation set appears to increase basal activity in b2AR and b1AR, but not b3AR. I wonder if the authors could comment on this and why they think these mutations increase basal activity in the first place."***

We agree that this is an interesting observation. The increased basal activity of the β_2R mutant reflects that the equilibrium between active and inactive receptor conformations is perturbed. We performed MD simulations to understand the molecular mechanism of activation for the wild-type and mutant β_2R. Calculations of

free energy landscapes describing activation show that the β_2R mutant M16 shifts the receptor conformation towards more active-like conformations, which is consistent with increased constitutive activity (Supplementary Figure S15). Comparison of the results for the WT, M4, and M24 clearly shows that it is the mutations in the TM3-TM4-TM5 interface that result in the basal activity of M16 (V117S/E122L/T123N/N312V), which is a combination of M4 (V117S/N312V) and M24 (E122L/T123N). As shown in Figure 6C, this is likely due to a more active-like hydrogen-bonding network involving N123^{3x42}, which is located in the TM2-TM3 interface. We describe these results on pages 14-15 and 22-24. The reason the β_1R and β_2R show more similar behaviour in the experiments (*e.g.*, selectivity and basal activity) is most likely their higher sequence similarity. In the TM region, the β_2R and β_1R share 66% sequence identity (TM region). In contrast, the sequence identity between β_2R and β_3R is lower in the TM region (55%, respectively). We have added these observations to the results (page 18).

5. Reviewer: ***"The authors discuss b2AR and D1DR structures on pages 7 and 8, but the references to this work is missing in this section."***

We added these references in the revised manuscript on page 8.

6. Reviewer: ***"Figure 4 is very overwhelming, and I wonder if this can be split across two figures to make it more palatable for readers. Panels A and B also lack dashed lines for the ionic interactions between the ligands and D3.32."***

As suggested by the reviewer, we have split the original figure into two separate figures and reduced the number of panels (Figure 5 and Figure 6). Figure 5 illustrates the effects of mutations within the binding site, while Figure 6 summarizes mutations outside the binding site and the MD simulation results. We have also added dashed lines in Figure 5A-B to indicate the ionic interaction between the ligands and residue D$^{3.32}$ (Page 20).

7. Reviewer: ***"The authors need to check the construct mutations in Table S1 as both structural constructs appear to list a S107V mutant, which is implausible."***

We thank the reviewer for identifying this error, which we have corrected in the revised supplementary information file (Table S3 on page 25).

8. Reviewer: ***"It is not explicitly stated whether the cAMP assays were performed in HEK or CHO cells."***

We appreciate the reviewer's suggestion to improve the clarity and the reproducibility of the cAMP assay methodology. We included the type of used cells (CHO-K1) in the revised manuscript (page 35).

Reviewer #3

The third reviewer wrote that our study ***"addresses an important question with broad implications for receptor biology and drug design"***. The reviewer requested further clarification regarding mutant selection, the interpretation of functional assays, and the link between structural/computational observations and pharmacological results. In the revised manuscript, we have addressed these concerns and also included the requested methodological details.

Major comments

1. Reviewer: ***"The logic linking bioinformatics predictions to mutagenesis is unclear. If five hotspots were identified, what guided the selection of the additional 120 receptor mutants? It appears the experimental strategy was more exploratory than hypothesis-driven, which is valid but should be acknowledged. Present the narrative accordingly rather than implying a strictly rational design."***

We thank the reviewer for this thoughtful comment. A total of 29 unique positions were mutated in our study, of which 11 were identified as hotspot residues by the sequence analysis. Of these, five were identified as particularly interesting (prioritized hotspot residues). The selection of the additional 24 positions was based on extensive analysis of the receptor structures, and several iterations of experimental testing were performed. Each of the 120 designed mutants was a result of a hypothesis originating from the analysis of both sequence and structural data, but we didn't strictly follow one strategy and explored several ideas during the study. In the new Supplementary Table S2, we present the rationale for selecting each the 29 evaluated positions. The mutated positions, which are all >80% sequence conserved in at least one receptor, can largely be divided into four categories:

(A) Hotspot residue: Conserved in both receptors with BLOSUM62 scores indicating a substantial difference between the receptors in this position.
(B) Hotspot region: Residue position located within 5 Å of a prioritized hotspot position, including the hotspot residue. These residues can interact with the hotspot position and thereby influence selectivity.
(C) Binding site region, first shell: Residue located within 4 Å of the endogenous ligand. These residues can affect selectivity through differences in interactions.
(D) Binding site region, second shell: Residue located within 4 Å of a first-shell binding site residue. These residues can affect selectivity by influencing the positioning of residues in direct contact with the ligands.

In addition to including the new Supplementary Table S2, we have revised the results section (pages 8-10) to describe the selection of mutants more clearly.

2. Reviewer: ***"Given the complexity of GPCR signaling (i.e., modalities differences, pathway biases, etc.), using functional assays to assess selectivity can confound interpretation, as signaling outputs may reflect changes in downstream pathway dynamics rather than true differences in ligand binding preferences. Furthermore, functional assays such as cAMP accumulation are susceptible to downstream amplification, complicating the interpretation of ligand selectivity. This likely explains the rather complex effects described in section "Binding site mutations reduce selectivity at the expense of efficacy and potency (P10-12)" and it further supported by the more modest selectivity profiles observed for M16 and M74 at the Gs recruitment assay, indicating that mutants are altering overall receptor signaling mechanisms rather than ligand recognition (and selectivity) motifs. Alternative approaches (e.g., binding affinity assays without overexpressed G proteins) should be used for the key mutants (including the single residue binding site mutants) to properly validate the claims on selectivity shifts."***

The aim of our study was to investigate how ligand selectivity profiles could be switched while maintaining the functional response, since signalling must be maintained during the evolutionary change of ligand preferences. As requested by the reviewer, we did attempt to perform binding assays during the project for a few key mutants. However, we found that the radioligands (*e.g.*, [^{3}H]-SCH-23390 and [^{3}H]-CGP-12177) showed poor or no binding to the mutants (*e.g.*, for β_2R-M4 and D_1R-M3), preventing characterization of ligand binding using this assay. This result can be explained by the fact that the labelled ligands are larger than adrenaline and dopamine, and are therefore more affected by the mutations. Moreover, saturation binding assays using the tritiated native ligands were not an option because dopamine and noradrenaline are unstable. Similar results from radioligand binding assays have been reported in several previous studies, *e.g.* for the β_2R mutation N312A (https://pubmed.ncbi.nlm.nih.gov/8101966/, Figure 1). In agreement with our results for β_2R-M4 and D_1R-M3, the authors were unable to detect radioligand binding to the N312A mutant. In addition, the N312A β_2R mutant (like our N312V mutant, which also replaces the asparagine with a hydrophobic residue) still responds to adrenaline, illustrating that radioligand binding may not be the most suitable assay for characterizing binding-site mutations. Due to these challenges, we instead decided to use the G protein recruitment as an orthogonal assay. In contrast to the cAMP experiments, the G protein recruitment assay exhibits minimal signal amplification. The reviewer correctly points out that the ligand selectivity of the mutants (β_2R-M16 and D_1R-M74, Figures 3C–D) in the G protein recruitment assay is modest. We agree with this observation, but also note that even for β_2R-WT, the potency differences in the Gs recruitment assay are considerably smaller than those observed in the cAMP assay (see Table R1, below). If this effect is taken into account, the cAMP and G protein recruitment assays are in good agreement. We have added these conclusions on page 16 of the revised manuscript.

Table R1. Comparison of selectivity profiles from cAMP and G protein recruitment assays.

Receptor	Assay	Selectivity: ADR vs DA (fold-difference)	Selectivity: NA vs DA (fold-difference)
β_2R-WT	cAMP	2500	63
	G_s	240	15
D_1R-WT	cAMP	30	18
	G_s	26	25

3. Reviewer: ***"Several mutants display increased basal activity or altered signaling profiles (e.g., Figs 3C and 3E), potentially indicating changes in constitutive activity rather than true selectivity shifts. These observations should be explicitly addressed and controlled for."***

We agree with the reviewer that the mutations lead to receptors with altered signaling properties, and that the changes in constitutive activity compared to the wild-type receptor are particularly interesting. As we are mutating residues involved in ligand recognition and receptor activation, this is not an unexpected result. In principle, we would not expect small changes in basal activity to affect selectivity. In the case of the β_2R, the mutations leading to basal activity are located in the TM3-TM4-TM5 interface (E122L/T123N). As an additional control of how these mutations affect selectivity, we evaluated β_2R-M24 (E122L/T123N) and compared to the results obtained for β_2R-WT. In β_2R-M24, basal activity increases but the selectivity profile characteristic of the wild-type receptor is maintained. A similar effect was observed when the E122L/T123N mutations are added to β_2R-M4, resulting in the receptor with switched selectivity profile (β_2R-M16). The changes in constitutive activity induced by the E122L/T123N mutant do hence not affect the observed selectivity profile. We have added this conclusion to pages 14-15 of the revised manuscript.

4. Reviewer: ***"Some apparent selectivity "switches" are actually loss-of-function across ligands, which does not support the claim of reciprocal ligand selectivity or the direct role of those residue positions as selectivity hotspots. Also, since residue positions in M16 and M74 do not overlap (apart from 3x36 and 7x38) and "are not transferable", they cannot be considered selectivity determinants, as these receptor variants are incomparable to one another and to their wild types."***

Unfortunately, we are having difficulty following the reviewer's reasoning in this comment. Our conclusion is that we may not have expressed our interpretations of the results clearly enough in the manuscript. Below, we address the reviewer's comments and describe the changes made to the manuscript.

First, it is correct that many mutations lead to a *"loss-of-function across ligands"*. However, we are not certain how this result connects to a *"claim of reciprocal ligand selectivity"*. The word *"reciprocal"* is not used in the manuscript. Possibly, the reviewer is referring to the idea explored in the paper that the same positions may determine selectivity in several receptors. What our data support is that several positions need to be exchanged to switch selectivity. Importantly, each individual mutation can lead to a loss-of-function: It is the combination of mutations that switches receptor selectivity. The main focus of our analysis has been the differences in potency between adrenaline, noradrenaline, and dopamine. Although the binding site mutations do not switch selectivity in most cases, there is typically a reduction of selectivity, supporting a role of these residues in determining ligand selectivity. We have added this conclusion to the manuscript on page 13.

Second, it is also correct that residue positions determining selectivity *"do not overlap"* and *"are not transferrable"* between subfamilies. However, we are not certain why this would lead the reviewer to conclude that these residue positions *"cannot be considered selectivity determinants"*. What our data support is that the same set of residues determines selectivity within a subfamily, but the mutations are not transferrable

between subfamilies. As evident from the many receptors and other protein classes that recognize the same chemical messenger, there is a large number of sequence combinations that lead to selectivity for a particular molecule. Our finding that different positions determine selectivity in different subfamilies is hence not an unexpected outcome of evolution. For more the closely related receptors (D_1-like dopamine and β adrenergic subtypes), the same positions could switch the selectivity, supporting a conserved mechanism for both ligand recognition and selectivity within subfamilies. In the revised manuscript, we have made changes to further clarify that the mutations switching selectivity are unique for each subfamily (*e.g.*, abstract, discussion, and the new Figure 4).

5. Reviewer: ***"The conclusion that this work enables rational ligand design is not supported by the presented data.***

Rational ligand design was mentioned in the abstract of the manuscript, and we have removed "rational" from this sentence (page 2). In the discussion, we describe how our results highlight strategies for drug design, *e.g.* in the design of selective or allosteric ligands (page 31).

6. Reviewer: ***The distinction between determinants of endogenous agonist selectivity vs pharmacological ligand selectivity should be clarified. In this regard, the manuscript title is misleading."***

In the revised manuscript, we have clarified that we focus on selectivity for chemical messengers in the last paragraph of the introduction (page 4) and first paragraph of the discussion (page 28). We also thank the reviewer for suggesting more specific title. We changed the title in revised manuscript to *"Molecular mechanisms of native ligand selectivity in G protein-coupled receptors"*.

7. Reviewer: ***"The phylogenetic analysis in Fig.1A could support a narrative where D1-like and β receptors share ancestral G protein coupling while diverging in ligand specificity—this should be discussed as it provides important context for the study's main claim. Moreover, the manuscript implies that ligand selectivity evolved independently of G protein preference, but this should be discussed in more detail, given evidence that Gs-coupling emerged earlier and was retained in catecholamine receptor evolution (c.f. https://pubs.acs.org/doi/full/10.1021/jm0511031)."***

We thank the reviewer for this important comment and are happy to clarify in the introduction. The schematic trees in Figure 1A summarize the receptor relationships based on either pharmacology or protein sequences. In the article cited by the reviewer, which analyses only the human sequences, the phylogenetic analysis was shown schematically and is largely equal to the right side tree in our Figure 1A (though our tree is more simplified). These relationships have been analyzed in more detail in several other studies, for instance reference 14 in our manuscript. As the G-protein coupling agrees with the protein phylogeny, the most parsimonious interpretation is that pharmacology (i.e., ligand selectivity) has changed during evolution. The reviewer is therefore right that G_s-coupling likely emerged earlier than ligand selectivity. In the revised manuscript, the important observation regarding G protein coupling has been

added in the introduction (page 4), and we also cite the work by Xhaard *et al*. mentioned by the reviewer (reference 13).

8. Reviewer: ***"The abstract's reference to "rational protein engineering" is overstated; the work is more consistent with a semi-rational or empirical approach, particularly given the variety of mutants tested and the limited gain-of-function results."***

We agree with the reviewer and have removed "rational" from the abstract.

9. Reviewer: ***"Statements such as "selectivity is governed by a few positions within the binding site" contradict experimental data showing either retention of native selectivity or loss of function, not a switch."***

We thank the reviewer for this comment. We have removed the sentence mentioned by the reviewer in the revised manuscript (page 26).

10. Reviewer: ***"There is no clear link between the section "Selectivity mechanisms in adrenergic, dopaminergic, and serotonergic GPCRs" and the rest of the manuscript. Is this web-based tool based on the evolutionary analysis or the residue positions actually swapped at the D1 and β2? Please, make this link clearer or remove this section, as it does not seem relevant to the present work."***

We thank the reviewer for this feedback. The web-based tool automates the same analysis that we performed for the β-adrenergic and D_1-like receptors to identify selectivity hotspots (pages 7-10). After presenting our work at conferences, we were approached by several research groups interested in comparing other pairs of receptors, and the web tool was developed to facilitate these analyses. The examples included also indicate that the molecular mechanisms we discovered for the β-adrenergic and D_1-like receptors are relevant for other receptor pairs. We have rewritten the first sentences of the paragraph to clarify how the tool is linked to the rest of our work (page 26), and we also moved this section to the end of the results section to improve the flow of the manuscript.

11. Reviewer: ***"Why were MD/FEP simulations performed on in silico mutated WT structures rather than the experimental cryo-EM models of the mutants? This reduces confidence in the predicted energetic and structural changes."***

The MD/FEP calculations were performed early in the project with the goal to guide the design of mutants, prior to obtaining the cryo-EM structures of the mutant receptors. At that point, the only available option for modelling of receptor variants was *in silico* mutagenesis of the wild-type experimental structures. In addition, the new cryo-EM structures only represent one ($β_2$R:3x36/7x38) of the six ($β_2$R: 3x36, 7x38, 3x36/7x38, D_1R: 3x36, 7x38, 3x36/7x38) mutants considered in the MD/FEP calculations. If we had initiated the calculations from the two new cryo-EM structures, we would also have needed to generate several mutants with *in silico* mutagenesis. In such cases, our experience is that initiating all the simulations based on a single carefully prepared structure is advantageous. We have added additional information in the methods section to clarify how we prepared the mutated receptor variants (page 44).

12. Reviewer: ***"MD and FEP results (e.g., "mutations at positions 7x38 and 3x36 led to a swap of agonist binding modes between the receptors" c.f. P21) do not align consistently with pharmacological data (e.g., for V117S and N312V), and inferences about ligand amine positioning are not directly assessed. These contradictions need explanation."***

As requested by the reviewer, we have analyzed the agreement between the MD simulations, FEP calculations, and pharmacological data. We focused in particular on the 7x38 and 3x36 mutations introduced in β_2R, which are specifically mentioned by the reviewer. The pharmacological data for the V117S/N312V shows a large loss of selectivity: From 50-fold selectivity for noradrenaline over dopamine to a 9-fold selectivity for dopamine over noradrenaline. The FEP calculations also predict a large loss of selectivity: From a 23-fold selectivity for noradrenaline over dopamine to 2-fold selectivity for dopamine over noradrenaline. There is hence a qualitative agreement between the FEP results and pharmacological data. However, the V117S/N312V mutation did show selectivity for dopamine (9-fold) in the pharmacological assays whereas our FEP calculations essentially predict that there is no selectivity (2-fold for dopamine), which we agree is an important difference. The results for the D_1R and corresponding mutants are comparable, and we provide a summary of the results for the 7x38/3x36 mutations below (Table R2). We have introduced this more detailed analysis of the V117S/N312V mutation on page 13 of the results.

Table R2. Comparison of experimental and calculated selectivity for noradrenaline (NA) and dopamine (DA) data for the 3x36/7x38 mutation.

	**Experimental selectivity**	**Calculated selectivity**
β_2R-WT	50-fold for NA	23-fold for NA
M4 (V117S/N312V)	9-fold for DA	2-fold for DA
D_1R-WT	30-fold for DA	17-fold for DA
M4 (S107V/V317N)	3-fold for DA	5-fold for DA

The reviewer's second question concerns how the mutations influence the ligand binding modes, in particular the amine positioning. In this case, we can compare the binding modes observed in MD simulations to β_2R and D_1R structures determined experimentally (PDB accession codes: 4LDO and 7LJD). In the case of β_2R-M16, we compared to simulations of β_2R-WT and D_1R-WT in complex with dopamine. For β_2R-M16 and D_1R-WT, the amine nitrogen was positioned closest to that observed in the D_1R experimental structure. In contrast, in the β_2R-WT simulation, the amine nitrogen was closer to the position observed in the β_2R experimental structure. In the case of D_1R-M74, we compared to simulations of β_2R-WT and D_1R-WT in complex with adrenaline. For D_1R-M74 and β_2R-WT, the amine nitrogen was positioned closest to that observed in the β_2R-WT experimental structure. In contrast, in the D_1R-WT simulation, the amine nitrogen was closer to the position observed in the D_1R experimental structure. In summary, these simulations support that the β_2R-M16 and D_1R-M74 mutants lead to a change of the ligand amine positioning, and that there is a swap of binding modes compared to the WT receptors. We have added these results in the new Supplementary Figure S13 and to page 21 of the revised manuscript.

13. Reviewer: ***"The use of a chimeric β2 structure in an intermediate/inactive-like state is problematic (PDB 4LDO). A better strategy would be to revert mutations in the new cryo-EM structures and simulate these."***

We thank the reviewer for raising this important point. The choice of the 4LDO structure reflects how the study was carried out and is based on the best practices of free energy calculations. The purpose of free energy calculations was to predict the effect of the mutations. For that reason, we could not have used the new cryo-EM structures because these were determined several years after these simulations were prepared. At the time this research project was initiated, the β_2R structure we used (PDB accession code: 4LDO) was the only one available with adrenaline bound. As our work focused on the endogenous ligands, we considered this structure to be the most suitable starting point for our MD simulations. This choice is in line with best practices of binding free energy calculations, which *"require at least one well-resolved cocrystal structure with one representative of the series of interest*" (10.1021/acs.jcim.0c00900).

The reviewer is also concerned that the structure may not represent an active conformational state. To assess if 4LDO represents an active state, we aligned this structure to the β_2R–G protein complex structure (PDB accession code: 3SN6). 4LDO is highly similar to the β_2R–G protein complex structure (PDB accession code: 3SN6), with an overall Cα RMSD of 0.56 Å for the receptor. Below, we show a structural alignment of the binding sites and the side chains in contact with adrenaline are located in very similar positions (Figure R2). The 3SN6 and 4LDO structures were both determined by Brian Kobilka's team. In agreement with our analysis of the receptor and binding site structure, the authors also state that 4LDO is an active structure (from abstract): *"Using directed evolution, we engineered a high-affinity camelid antibody fragment that stabilizes the active state of the β_2R, and used this to obtain crystal structures of the activated receptor".* To summarize, the 4LDO structure represents and active-like state and was the most suitable choice for our simulations because adrenaline is bound. We have added this information to the methods section of the manuscript (page 43).

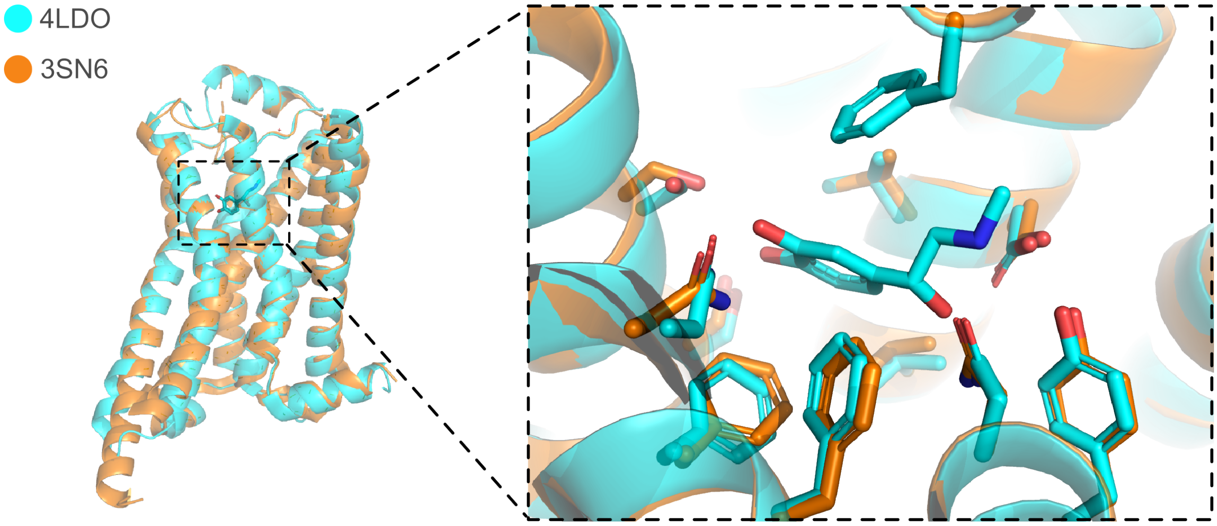

Figure R2. Alignment of the 4LDO (cyan) and 3SN6 structures (orange). Adrenaline and the residues in contact with this ligand are shown as sticks.

14. Reviewer: ***"The selection and processing of sequence data for UMAP need to be better justified. Why were 97 sequences used instead of canonical human variants? Which other sequences are these?"***

We thank the reviewer for this comment. The UniProt reviewed sequences used in the UMAP analysis represent 16 different species, which are all major mammals. Compared to using only human variants of the receptors, sequences from different species will be more diverse and hence provide more information regarding which residues are key for determining ligand selectivity. The clustering of subfamilies in the UMAP analysis shows that we are capturing sequence differences independent of species, which is consistent with the idea that the mechanism of selectivity is conserved across species. We have included information about the species used in our analysis and updated the number of sequences (83 instead of 97) in the methods section (pages 41-42).

15. Reviewer: ***"Clarify how the one-hot encoding was performed for UMAP, and justify why PCA was not used instead, since the plot of PCA loadings would provide interpretable dimensions and may highlight subtype differences more clearly."***

We also agree with the reviewer that PCA can provide more interpretable results. However, in this part of the manuscript, our focus was to complement the phylogenetic analysis with a dimensionality reduction method to visualize differences and similarities among catecholamine receptors. The UMAP analysis is consistent with the phylogenetic analysis of the full sequences, demonstrating that the binding sites of β_2R and D_1R are very similar. For these purposes, both PCA and UMAP are appropriate tools, and it would be redundant to complement the phylogenetic and UMAP analyses with a third method.

We thank the reviewer for pointing that our description if the UMAP analysis was not clear. In the revised manuscript, we decided to use a single approach for the one-hot encoding, reducing the number of panels from four to two in Supplementary Figure S1. One-hot encoding was performed by converting each residue in the multiple sequence alignment into a binary vector of length 21, representing the 20 standard amino acids plus the gap character "–". We have included this information in the methods section (page 41-42).

16. Reviewer: ***"Provide supporting data (e.g., BLOSUM62 scores) for the identification of mutational hotspots. Clarify which of these residues were actually mutated, and explain discrepancies."***

In the revised manuscript, we have introduced Supplementary Table S1, which summarizes the BLOSUM62 scores and criteria used for identification of the three hotspot regions. If the first step, we analyzed residues with 80% sequence conservation with BLOSUM62 scores indicating a substantial difference between the receptors (score < 0). In order to make the analysis process clearer to the reader, we now include all the 11 positions that fulfill this criterion in the Supplementary Table S1. In a second step, we analyzed the β_2R and D_1R structures extensively and assigned lower priority to six positions that were primarily surface-exposed and therefore unlikely to affect ligand binding, or that were close to the G protein binding site, where mutations could interfere with the signaling assays. The remaining hotspot positions

were either interacting directly with the endogenous ligands (positions 3x36, 45x52, and 7x38) or located in two other distinct regions involved in interhelical interactions (positions 2x60 and 3x41).

As shown in the new Supplementary Table S2, which summarizes all mutated positions, the five prioritized hotspot positions were mutated for both the β_2R and D_1R. For example, hotspot positions 3x36 and 7x38 were present in 65 and 109 of the 120 evaluated mutant receptors. Mutations in hotspot positions 1x54 and 6x46 were also explored in a few mutants for the D_1R, but with substantially lower priority (7 and 1 mutants, respectively). If we sum the frequencies in Supplementary Table S2 for the five prioritized hotspots, they were selected for testing at 261 occasions, and the corresponding number for the remaining six (deprioritized) hotspots were is only eight. We hence didn't strictly focus on the five prioritized hotspots, but identification of these regions strongly influenced the design of mutants. In addition to introducing the two new Supplementary Tables S1 and S2, we have also rewritten the results text to make the identification of hotspot positions and selection of mutations clearer to the reader (pages 7-10).

17. Reviewer: ***"AlphaFold3 models offer little added value here; with cryo-EM structures available, focus on the experimental data and simulations."***

We agree with the reviewer that the analysis of the cryo-EM structures and MD simulations should be the primary focus. However, we find it noteworthy that the AlphaFold3 models display similar conformational changes and ligand-binding modes. We now mention these results briefly at the end of the section describing the cryo-EM structures (page 25).

Minor comments

18. Reviewer: ***"Figure 3 is difficult to interpret. Improve labeling and color coding; include WT curves for reference and display more single mutant curves for clarity."***

As suggested by the reviewer, we have added additional single-mutant curves to Figure 3 and improved the layout (including labeling and color coding). We also tried to simplify interpretation of the figures by including the wild-type curves. Due to the increased size of the revised figure, we have moved panels D and E to a new Figure 4.

19. Reviewer: ***"Supplementary figures (e.g., S4, S5, S16) lack clear labeling and do not match the text descriptions. Indicate residue identities and transformation directions clearly."***

We thank the reviewer for pointing out that these figures were not clear. We have improved the labeling in each of the three mentioned figures (S4, S5 and S14 [previously S16]) in revised supplementary information.

20. Reviewer: ***"Page 11: "descried" should be "described." "***

We thank the reviewer identifying this error, which we corrected in the revised manuscript (page 45).

21. Reviewer: ***"Check significant figures and precision of pEC50 values across the manuscript."***

In the revised manuscript, we use three significant figures for all pEC_{50} values, and have also included uncertainties for each value (two significant figures).

22. Reviewer: ***"Ensure all receptor subtypes are clearly labeled in figures and graphs (e.g., Figure 3)."***

We thank the reviewer for this important suggestion, we doubled checked labels in all figures in revised manuscript and supplementary information files.

23. Reviewer: ***"Figure 2 describes the research pipeline and is suitable for a grant application but not for a research paper (e.g., why is β-arrestin included if not assayed in the study?) Instead, use this space the present the results or to illustrate, for example, where the actually mutated residues are located in the receptors and how these residues compare to their counterparts in the other dopamine and adrenergic subtypes (sequence alignments of the 7+4 positions)."***

We thank the reviewer for these suggestions. As requested, we have removed the signalling pathways and illustrations of the different experiments performed in our study (panel D). We have also moved panel C to Figure 4, which complements the experiments assessing transferability of the mutations. In addition, the snake plot now illustrates where mutated residues are located for each receptor.

24. Reviewer: ***"Improve figure captions for S16 and S5 to explain which atom pairs/distances are being measured and the context for those results."***

We thank the reviewer for these suggestions. We have improved the images and legends of Figures S5 and S14 (previously S16) in the revised Supplementary Information.

25. Reviewer: ***"Missing details on the protein preparation for modeling, including treatment of missing loops and missing side chains, removal of nanobodies/transducer proteins, reversion of thermostabilizing mutations, etc."***

We thank the reviewer for the suggestion. We have added additional details on protein preparation in the revised manuscript, including treatment of missing loops, side chains, thermostabilizing mutations, nanobody/transducer proteins (pages 43-44).

26. Reviewer: ***"Ensure that Figure 2 and S3/S4 consistently represent the same positions. Currently, highlighted residues and tested mutants do not correspond. Otherwise, explain the discrepancies."***

In the revised manuscript, we show the same set of residues in Figure 2 and S3 (the positions that were mutated in our study).

27. Reviewer: ***"The definition of binding site residues is unclear and should be explicitly described in the Methods. List the residues."***

We thank the reviewer for this comment. Two different definitions of the binding site were used in our analysis. In the comparison of only the β_2R and D_1R, we defined the binding site positions as the residues with atoms within 4 Å of the endogenous agonist in the experimentally determined structures (PDB accession codes: 4LDO and 7LJD, respectively). We show these residues in Figure 1B (Positions: 3x28, 3x32, 3x33, 3x36, 45x52, 45x52+2, 5x43, 5x461, 6x51, 6x52, 6x55, 7x38, 7x42) and have also added the set to the methods section in the revised manuscript (pages 41-42). In the analysis of the adrenergic, dopaminergic, and serotonergic receptors, we used a more general definition of the binding site of aminergic GPCRs based on reference 17 (amine and major binding pocket: 3x32, 3x36, 6x48, 6x51, 7x38, 7x42, 3x33, 3x37, 3x40, 4x56, 4x57, 5x39, 5x40, 5x43, 5x44, 5x461, 5x47, 6x44, 6x52, 6x55, and 6x56). As requested by the reviewer, we have also added these positions in the methods section (pages 41-42).

Reviewer #4

The fourth reviewer ***"very much enjoyed reading the manuscript"*** and wrote that our work ***"will be very significant for researchers in the GPCR field aiming to understand molecular mechanisms of selectivity as well as the evolution of GPCR ligand binding and will also be of interest to researchers outside of the GPCR community".*** In the revised manuscript, we have addressed all of the reviewer's comments, and our detailed responses are provided below.

1. Reviewer: ***"They further show that β2R and D1 receptors are phylogenetically more closely related to each other than β2R to the α-adrenergic receptors and D1 to the D2 receptor. This is noteworthy because one would have expected a different result given the pharmacological profiles of the receptors. It would be interesting to know why these results disagree with phylogenetic trees obtained elsewhere (e.g. GPCRdb.org)."***

We appreciate the reviewer's observation and agree that the discrepancy between the phylogenetic and pharmacological classifications is intriguing. As requested by the reviewer, we generated a phylogenetic tree using GPCRdb (https://gpcrdb.org/) and compared the results with those shown in Figure 1A of our manuscript. As shown below (Figure R3), our phylogenetic tree (Figure 1A) is consistent with the GPCRdb results. It should also be noted that the phylogenetic relationships among the catecholamine receptors have been described previously (*e.g.* in reference 14, Figure 1). In the revised manuscript, we have added a citation in the legend of Figure 1A to clarify that this observation is based on earlier work.

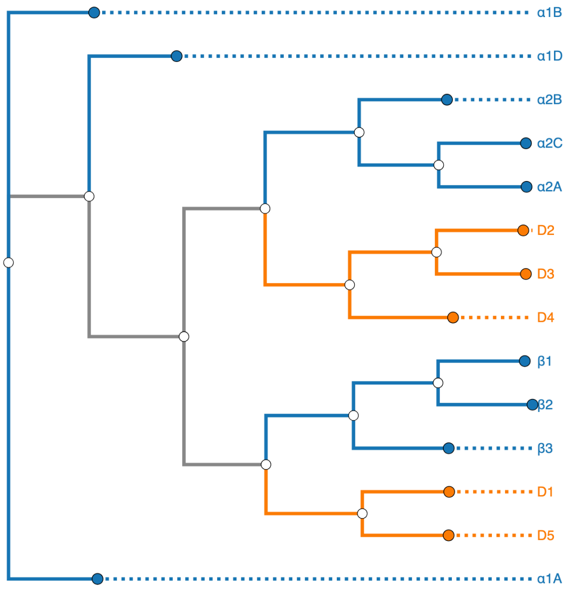

Figure R3. Phylogenetic tree for catecholamine receptors obtained from GPCRdb. The tree was generated based on the TM region of the human catecholamine receptors using the default phylogenetic trees setting. The classification of the receptors is the same as that shown in Figure 1A of the manuscript.

2. Reviewer: ***"Could the authors provide more details on the multi-species sequence alignments used for the identification of receptor selectivity hotspots? A supplementary table including conservation and BLOSUM62 scoring would be helpful. Please provide a rationale why seven positions were selected. Were these the only residues with >80% conservation and a BLOSUM62 score <0?"***

As requested by the reviewer, we have included Supplementary Table S1, which summarizes the sequence conservation, BLOSUM62 scores, and the rationale for identifying selectivity hotspot regions. A total of 11 residues had >80% conservation and negative BLOSUM62 scores. We then inspected these hotspot positions in the experimental β_2R and D_1R structures and deprioritized six positions that were either surface-exposed and therefore unlikely to affect ligand binding, or that were close to the G protein binding site, where mutations could interfere with the signaling assays (Supplementary Table S1). This resulted in prioritization of five hotspot residues, which were located in three different regions of the receptors. In addition to adding Supplementary Table S1, we have also revised the results text to clarify to the reader how we identified selectivity hotspots.

3. Reviewer: ***"Please provide a rationale for the inclusion/exclusion of neighbouring residues around the hotspots. Why did you choose these residues for residue exchange?"***

We thank the reviewer for highlighting this important point. In the revised manuscript, we have included Supplementary Table S2, which lists the rationale for mutating the 29 unique positions considered in our study. All the mutated positions are highly conserved (>80%) in at least one subfamily and different between the receptors. There are in total eight positions that are neighbouring a hotspot residue (2x64, 3x42, 45x52+2, 4x49, 4x52, 7x34, 7x35, and 7x43). These were selected based on analysis of experimentally determined structures of the β_2R and D_1R:

• Two positions are in direct contact with the ligand: 45x52+2 and 7x42.
• Two positions are involved in an interhelical interaction network that the hotspot residue 2x60 is part of: 2x64 and 7x35.
• One position is involved in interactions with a binding site residue: 7x34.
• Two positions close to hotspot residue 3x41 are located in interfaces between helices: 3x42 and 4x49.
• One position interacts directly with the hotspot residue 3x41 side chain (4x52).

We have rewritten the section in the manuscript focusing on selection of residue positions for mutagenesis (pages 7-9) and the detailed description of the selection is added in the footnotes of Supplementary Table S2.

4. Reviewer: ***"This analysis reduced the number of differences in the alignment from ~100 to seven positions' – consider rephrasing, as the number of differences in the alignment stays the same, but the number of positions considered for further analysis is reduced."***

We agree with the reviewer and have removed this sentence in the revised manuscript.

5. Reviewer: ***"Please provide additional information on the normalisation of the signalling data to WT expression. How exactly was this normalisation performed? Was WT signalling measured for different expression levels?"***

We thank the reviewer for noting that the normalization method was not clearly described in the manuscript. Actual cAMP concentrations were normalized to wild-type D_1R or β_2R maximum response to the cognate ligand (100%) before averaging. A local maximum response control was also included in each experiment for each plate. Receptor expression was determined in experiments separate from the functional assays, and we did not evaluate signalling at different expression levels. As ligand selectivity of the wild-type and mutant receptors was consistent in both the cAMP and the less-amplified G protein recruitment assays, the expression level is unlikely to affect the key results of the work. We have added the missing information to the methods section of the revised manuscript (page 35).

6. Reviewer: ***"Will the code used in this manuscript be shared, e.g. on GitHub?"***

We are pleased that the reviewer is interested in our code. In addition to providing the webserver, we will make the code publicly available on GitHub once the manuscript has been accepted for publication. In the revised manuscript, the code/software is provided in a zip file with a readme.txt file for installing and running the software. We also provide example data and expected output.

7. Reviewer: ***"In Figure 3B, a graph for WT β2R and D1R, an additional label specifying the mutant (e.g. M3 for the N312V mutant of β2R), and a matching size of the graphs would help compare the effect of mutations."***

We appreciated the reviewer's suggestions and have revised Figure 3 accordingly. We have included the wild type curves (Figure 3A and Figure 3B), added mutant labels, and unified the sizes of the curves.

8. Reviewer: ***"'Dose-response curve' and 'concentration-response curve' are used interchangeably in the manuscript. Consider using 'concentration-response curve' throughout."***

We thank the reviewer for highlighting this point. In the revised manuscript, we use the term "concentration-response curve" as requested by the reviewer.

9. Reviewer: ***"Consider stating the error of the cAMP pEC50 determination in the main text"***

We thank the reviewer for this suggestion and have included the error (SEM) for each pEC_{50} in the manuscript. We did not highlight these changes in the revised manuscript.

10. Reviewer: ***"Abbreviations should be explained when they are used for the first time (please double check for PDL and PEI)"***

We agree with the reviewer and have provided explanations for all the used abbreviations. We did not highlight these changes in the revised manuscript.

11. Reviewer: ***"Please specify the type of PEI used (linear vs branched, manufacturer)"***

As requested by the reviewer, we have included the information regarding the type of used PEI in the revised manuscript (page 37).

12. Reviewer: ***"Please detail the expression conditions in the 'Structural biology experiments' section. How much DNA and PEI were used? Which type of PEI was used and where was it purchased?"***

As requested by the reviewer, more detailed information regarding the DNA and PEI has been included in the revised manuscript.

13. Reviewer: ***"In the 'Structural biology experiments' section, the source/manufacturer for Expi293F cells, PEI, DDM, LMNG, M1-FLAG, CHS are missing. Which media were used? Were the cells lysed before solubilisation and if yes, how? What was the concentration of the detergents? The buffer used for solubilisation is missing. How was the detergent exchanged? How much of which buffer was used? Which SEC column was used? What was the final concentration of the receptor constructs after concentration? What was the concentration of the ligands? Please state the methods for the purification of Gβγ, Nb35 and scFv16 or cite the relevant literature."***

We thank the reviewer for this suggestion, and we have provided a more detailed description of the "Expression and purification of the proteins" in the "Structural biology experiments" section in the revised manuscript (page 37-40).

14. Reviewer: ***"Please double check the following sentence: 'Free energy perturbation calculations were then performed using the software Q72 using and its OPLS all-atom force field version'"***

We thank the reviewer for this comment, we have corrected the sentence in the revised manuscript (page 44).

Reviewer #5

The fifth reviewer wrote that our work addresses ***"a long-standing question"*** and ***"is highly useful and relevant for the field"***. The reviewer recommends publication in Nature Communications after addressing the following points:

Major comments

1. Reviewer: ***"Throughout the manuscript, the term "selectivity" is used without any clarification. The authors should clarify***
A) Which selectivity is calculated – Is it potency selectivity?
B) Which potency values were used for these calculations? This is indeed very vague in the manuscript, as the authors have used two different assays, G protein recruitment and cAMP assays, to determine the potency and efficacy of the receptor. Therefore, it is crucial to mention which potency values were used."

We thank the reviewer for pointing out that our definition of selectivity was not clear. In our study, "selectivity" refers specifically to potency selectivity from either cAMP or G protein recruitment assays. We primarily used cAMP assays and complemented with recruitment experiments for the most relevant receptor mutants. In the revised manuscript, we explain how we assess selectivity of wild-type and mutant receptors on pages 6 and 9, respectively.

2. Reviewer: ***"Figure 2 and S3. It is not clear if the authors intentionally focused on more or less the upper half of the receptors to identify the residues involved in determining selectivity, or what is shown in these figures is the result of a global analysis."***

In the identification of selectivity hotspots, we excluded residues that were close to the G protein binding site, which could interfere with the signalling assays and are less likely to influence ligand selectivity. This choice did lead to more focus on the residues in the upper-half of the receptors, as shown in the new version of Figure S3. However, a few positions (*e.g.*, 3x41 and 3x42) are located on the lower half of the receptor. In the revised manuscript, we have included a more detailed results section (pages 7-10) and Supplementary Table S1 to clarify how the selectivity hotspots were identified.

3. Reviewer: ***"Figure S4. Did the authors consider any cell surface expression threshold to decide whether or not to consider a mutation? For instance, M9 at S4.B shows a >70% reduction in the cell surface. Was this mutation considered regardless?"***

We agree with the reviewer that characterization of cell surface expression is important, and we performed immunostaining analysis of a large number of receptor mutants during the study. In the revised manuscript, we have analysed the results for all the mutants discussed in the manuscript, and the expression levels are >40% of the wild-type. For comparison, a recent Science paper focusing on evaluation of β_2R mutants (ref. 31, https://www.science.org/doi/epdf/10.1126/science.adh1859) used an expression threshold of 25% of wild-type. D_1R-M9 (expression level = 28%) is not discussed in the manuscript and the results for this mutant do not influence any of the conclusion of the manuscript. Notably, the key mutants with switched selectivity profiles (β_2R-M16 and D_1R-M74) exhibit expression levels comparable to those of the wild-type receptors (108% and 118%, respectively). In the revised manuscript, we only include surface expression levels for the key mutants that are discussed in the manuscript in Supplementary Figure S4, and the remaining are shown in Supplementary Datafile 1. We also mention the expression levels for the key mutants on page 10 of the revised manuscript.

4. Reviewer: ***"Figure 3. While all the CRCs start from the zero point of the plot, in the C part –cMAP response, the curve starts at ~30%. The authors should explain the reason. Were the cAMP responses normalized to the vehicle response? If so, why in the C part, the curve does not start from 0?"***

We thank the reviewer for these relevant questions. The actual cAMP concentrations were normalized to the maximum responses of wild-type D_1R or β_2R to their cognate ligand (100%) before averaging. We did not normalize to vehicle to be able to detect effects on the basal activity. Most mutant receptors exhibited very low basal activity, which may appear as 0% in the figures. However, as noted by the reviewer, a few mutants also displayed a marked increase in constitutive activity. In the revised manuscript, we have clarified this in the relevant figure legends (Figure 3 and 4, and Supplementary Figure S6) and methods section (page 35).

5. Reviewer: ***"To have a deeper understanding of the molecular mechanism that governs the selectivity, the authors should run MD simulations for the receptors with mutations in the binding pocket and the outside ones separately and compare the results with the current system, which has both site mutations. These would allow for a better understanding of the combinatorial role that these two sites play and the process that leads to the rescue of potency and efficacy, while maintaining the selectivity."***

As suggested by the reviewer, we have performed additional simulations of β_2R and D_1R mutants to gain additional insights into the molecular mechanisms of receptor activation and selectivity. To complement the simulations of the β_2R-M16 and D_1R-M74 that have all the key mutations, we extended our analysis to include receptors with mutations either within the binding pocket (β_2R-M4: N312V/V117S and D_1R-M14: V317N/S107V/W321Y) or outside the binding pocket (β_2R-M24: E122L/T123N and D_1R-M85: K81G/E85I/D314I/A147V).

For the β_2R, we identified that mutations in the TM4-TM4-TM5 interface lead to increased potencies and higher basal activity, which may be related to stronger hydrogen bonding between T/N123 and S74 observed in simulations of β_2R-M16 (Figure 6). Comparisons of β_2R-WT to β_2R-M24 and β_2R-M4 to β_2R-M16 show that the addition of the mutations in the TM3-TM4-TM5 interface consistently leads to the increase of hydrogen bonding frequency in the simulations, a result that is consistent experimental data showing an increase of basal activity for both β_2R-M16 and β_2R-M24 but not β_2R-M4. We have added these new results to the results section of the revised manuscript (page 24) and as Supplementary Figure S16A.

For the D_1R, we have added an analysis of the W318^{7x39} side chain rotamer for the receptors with mutations either within (D_1R-M14) and outside (D_1R-M85) the binding site. In this case, we observed that the D_1R-M74 mutant led to a side chain rotamer distribution that was more similar to that observed for the β_2R-WT compared to the D_1R-WT. The rotamer distributions of D_1R-M14 and D_1R-M85 were essentially the same as that of the D_1R-WT whereas combining the mutations within and outside the binding site (D_1R-M74) led to a large shift towards the β_2R-WT distribution, indicating a synergistic effect. We have added these new results to the results section of the revised manuscript (page 25) and as Supplementary Figure S16B.

6. Reviewer: ***"Kahlous et al propose two distinct mechanisms contributing to switching receptor selectivity. They propose that in the b2R, TM3/TM4/TM5 interface connects the ligand binding site to microswitches that control conformational changes and mutations that increase the receptor response to agonist binding, resulting in potencies closer to physiological concentrations. The second mechanism, i.e., for D1R, they hypothesize that residues in the TM2-TM7 interface, which surround the orthosteric ligand binding pocket, are critical for the switch in the selectivity profile of D1R. To prove their hypothesis on the allosteric effect of the second region on the potency and efficacy rescue in the orthosteric pocket, the authors should run network analysis for the WT and mutant systems (orthosteric pocket only, second region only, and both sites together) to examine whether the residue movements in these areas are correlated. If the mechanism is conserved, as claimed by the authors, this is expected to be reflected in this analysis."***

We thank the reviewer for this suggestion. In the revised manuscript, we have performed network analysis based on MD simulations of wild-type and mutant receptors to assess if there is allosteric communication between the regions within and outside the binding site.

For the D_1R, we performed network analysis using simulations of the wildtype, D_1R-M74 (mutations within and outside the binding site), D_1R-M14 (mutations within the binding site), and D_1R-M85 (mutations outside the binding site). We used the networks to assess how each of the mutated residues outside the binding site (positions 2x60, 2x64, and 7x35) influence the binding site residue closest to the 2x60 hotspot (7x42), a residue that was mutated in the receptor with switched selectivity. The calculated communication pathways showed that residues 2x60, 2x64, and 7x35 are part of two communities (i.e., groups of residues with highly correlated motions) that connect the residues within and outside the binding site. These results complement the data shown

in Figure 6D-E, which shows this interaction network contributes to stabilizing the W318^{7x39} and Y321^{7x42} side chains of the D_1R mutant in conformations characteristic of the wild-type β_2R, thereby favouring the binding of noradrenaline and adrenaline over dopamine. In the case of the β_2R, we also performed network analysis for wild-type and relevant mutant receptors (M4, M24, and M16). We then analyzed the communication pathways between the binding site residue S207^{5x461}, which is part of the TM5 bulge microswitch, and the mutated positions 3x41 and 3x42 in the TM3-TM4-TM5 hotspot region. The network analysis showed that S207^{5x461} is connected to the residues in the hotspot region (3x41 and 3x42) via residue and I121^{3x40}. Interestingly, I121^{3x40} is part of the PIF motif, an important microswitch for receptor activation that connects the agonist pocket to the G protein binding site. We have added the results from network analysis on pages 24-25 of the revised manuscript and in Supplementary Figure S17.

7. Reviewer: ***"In the discussion section, authors write that "Unexpectedly, mutations in the binding site primarily led to a loss of response to the native agonist, rather than switching the selectivity profiles and increasing the potency of the new ligand.". Indeed, there are numerous studies in the literature that describe the detrimental effect of mutations in the orthosteric pocket of class A GPCRs on affinity, potency, and efficacy in different cell-based assays. This is actually quite expected, as mutations in this site change the profile of the pocket geometrically and chemically, influencing the binding of a given ligand. Authors should argue why this was unexpected to them."***

We thank the reviewer for this comment and agree that many studies have demonstrated a loss of potency due to binding site mutations. We have revised the sentence mentioned by the reviewer and removed the word "unexpectedly" (page 28).

Minor comments

8. Reviewer: ***"Figure 4. The colors picked for the b2-adrenergic receptor in complex with adrenaline and dopamine are very close, hindering pose comparison. I recommend using more distinct colors."***

We thank the reviewer for pointing this out. In the revised manuscript, we have changed the colour used to depict β_2R in complex with dopamine (Figures 5 and 6, previously Figure 4) and enlarged the images. These changes facilitate comparison of the ligand poses.

9. Reviewer: ***"Figure S14 and S15. The authors are encouraged to follow a consistent labelling. It would be better to add "Cryo-EM" to the label "D1R-WT-dopamine" in S14, similar to S15."***

As suggested by the reviewer, we use consistent labelling in Supplementary Figure S12 (previously S14) and S18 (previously S15) of the revised manuscript.
